# Animal contact as a source of human non-typhoidal salmonellosis

**DOI:** 10.1186/1297-9716-42-34

**Published:** 2011-02-14

**Authors:** Karin Hoelzer, Andrea Isabel Moreno Switt, Martin Wiedmann

**Affiliations:** 1 Department of Food Science, 410 Stocking Hall, Cornell University, Ithaca, NY 14853, USA

## Abstract

Non-typhoidal *Salmonella *represents an important human and animal pathogen world-wide. Most human salmonellosis cases are foodborne, but each year infections are also acquired through direct or indirect animal contact in homes, veterinary clinics, zoological gardens, farm environments or other public, professional or private settings. Clinically affected animals may exhibit a higher prevalence of shedding than apparently healthy animals, but both can shed *Salmonella *over long periods of time. In addition, environmental contamination and indirect transmission through contaminated food and water may complicate control efforts. The public health risk varies by animal species, age group, husbandry practice and health status, and certain human subpopulations are at a heightened risk of infection due to biological or behavioral risk factors. Some serotypes such as *Salmonella *Dublin are adapted to individual host species, while others, for instance *Salmonella *Typhimurium, readily infect a broad range of host species, but the potential implications for human health are currently unclear. Basic hygiene practices and the implementation of scientifically based management strategies can efficiently mitigate the risks associated with animal contacts. However, the general public is frequently unaware of the specific disease risks involved, and high-risk behaviors are common. Here we describe the epidemiology and serotype distribution of *Salmonella *in a variety of host species. In addition, we review our current understanding of the public health risks associated with different types of contacts between humans and animals in public, professional or private settings, and, where appropriate, discuss potential risk mitigation strategies.

## 1. Public Health and Economic Costs Associated with *Salmonella*

*Salmonella *is an important foodborne pathogen world-wide. A recent study estimated that approx. 93.8 (95% Confidence Interval: 61.8-163.6) million human cases of gastroenteritis and 155 000 (95% Confidence Interval: 39 000 - 303 000) deaths occur due to *Salmonella *infection around the world each year [1]. In the USA alone, *Salmonella *causes an estimated 1.4 million human cases, 15 000 hospitalizations and more than 400 deaths annually [2,3]. However, only a fraction of cases is reported, and in the USA, only an estimated 1-5% of cases are laboratory confirmed and reported to the Centers for Disease Control and Prevention (CDC) [4]. In 2006, the national case rate in the USA equaled 13.6 reported cases per 100 000 population per year [4]. Rates varied considerably by geographic region, with estimates particularly high in the Mid-Atlantic and New England States. This heterogeneity is likely in part due to differences in reporting. Differences in salmonellosis case rates between geographically and socio-economically similar USA states have been documented, with rates differing by as much as 200% between neighboring states [4]. Similarly, of the 168 929 human cases reported in the European Union (EU) during 2005, 31% stemmed from Germany even though less than 20% of the EU's population resides in Germany, again suggesting reporting differences [5,6].

In 1999, non-typhoidal *Salmonella *infections in the USA were estimated to contribute 10% of foodborne human illnesses, 26% of hospitalizations, and 31% of deaths attributable to infections by known foodborne pathogens, thereby ranking first among all bacterial foodborne pathogens in hospitalizations and deaths and second after *Campylobacter *in the number of illnesses [3]. In 2009, *Salmonella *was the most commonly reported bacteriological agent of human foodborne disease in the USA, causing approx. 44% of confirmed foodborne bacterial infections [7]. More than 20% of human clinical *Salmonella *isolates in the USA are obtained from children under the age of 5 years, emphasizing the great importance of this age group [8,9]. Approx. 1% of *Salmonella *cases are thought to require hospitalization [10]. However, due to the high prevalence of *Salmonella *infections, the Economic Research Service of the United States Department of Agriculture (USDA) [11] estimated the annual cost inflicted on the USA economy to equal approx. 2.5 billion US dollar, thereby clearly exceeding the annual economic cost attributable to human infections by *Escherichia coli *($460 million) or *Listeria monocytogenes *($2 billion) [12].

## 2. *Salmonella *Epidemiology and Transmission Dynamics

*Salmonella *serotypes can be divided into host restricted, host specific, and generalists serotypes, with important implications for epidemiology and public health [13]. Host specific serotypes, for instance serotypes Paratyphi A, Gallinarum biovars Gallinarum and Pullorum, or Typhi only cause disease in one host species [13,14]. In contrast, host restricted serotypes are predominantly associated with one host species, but can cause disease in other species as well [13]. *Salmonella *Dublin, for example, is adapted to cattle but infections in small ruminants, pigs and humans have also been documented, and serotype Choleraesuis is adapted to swine but has also been isolated from a range of other host species [15,16]. Generalist serotypes such as *Salmonella *Typhimurium commonly cause disease in a broad range of hosts, even though a narrow host range has been described for certain subtypes, for instance Typhimurium subtypes DT2 and DT99, which appear to be adapted to pigeons [17]. Serotypes with broad and narrow host range seem to differ in clinical manifestation even though other factors such as host species, age, and concomitant disease affect the clinical manifestation as well (see [17] for a comprehensive review). Infections with generalist serotypes are often characterized by high morbidity but low mortality, and gastro-intestinal symptoms are the predominant clinical manifestation [13,17]. On the contrary, infections with host adapted or restricted serotypes, such as Choleraesuis, Abortusequi, Gallinarum biovar Gallinarum, or Gallinarum biovar Pullorum, are typically characterized by low morbidity and high mortality, and systemic disease is common [13,17].

*Salmonella *serotypes clearly seem to differ in their pathogenic potential for humans and serotype distributions often vary vastly between human and animal populations as well as among different animal populations in the same geographic area (see Additional file 1: Table S1). For instance, approximately 40% of all known *Salmonella *serotypes are predominantly associated with reptiles or amphibians, yet less than 1% of human salmonellosis cases are caused by these reptile-associated serotypes. The molecular determinants of serotype-specific host range differences have so far largely remained elusive. However, serotype-specific differences in virulence have been characterized in some cases. For instance, in competition experiments with *Salmonella *Typhimurium, reptile-associated *Salmonella *Arizonae and Diarizonae showed a significantly reduced ability to colonize and persist in the intestine of BALB/c mice, clearly suggesting virulence differences [18].

Majowicz et al. [1] recently estimated that approx. 80.3 of 93.8 million human *Salmonella*-related gastroenteritis cases that are estimated to occur globally each year are foodborne, thus representing approx. 86% of human salmonellosis cases. Another study based on formal elicitation of expert opinion estimated that approx. 55% (range 32-88%) of human *Salmonella *cases are foodborne, 14% (range 3-26%) are travel-related, 13% (range 0-29%) are acquired through environmental sources, 9% (range 0-19%) occur due to direct human-to-human transmission and 9% (range 0-19%) are attributable to direct animal contact [5,19]. Yet another study, based on surveillance data, estimated that 95% of non-typhoidal human *Salmonella *cases in the USA are foodborne, emphasizing the complexity and controversy of the subject matter [3]. Food products derived from the animal species discussed in this review can also serve as sources of human infection, and such products have been implicated in numerous human outbreaks. However, as this review focuses on animal-acquired infection, foodborne infections will not be further discussed here. Several excellent reviews of foodborne salmonellosis have been published in recent years, and the reader is referred to these publications for further details regarding infections associated with the consumption of meat, eggs, dairy products, vegetables, reptile products, and other foods (see for instance [10,20-29]).

The comparison of *Salmonella *outbreak and surveillance data across geographic regions or from different time periods represents a considerable challenge. The advent of novel molecular subtyping methods has noticeably improved discriminatory power, with important impacts on sensitivity and specificity [30]. The impacts of differences in public health infrastructure, disease surveillance sampling plans, public health legislation etc. are difficult to measure, but differences in apparent prevalence between states or countries are clearly noticeable. Case and outbreak definitions are also variable. For clarity, we will henceforth define "case" to refer to any instance where strong epidemiological or molecular evidence suggests an animal source of human infection, while trying to point out instances where such conclusions are solely based on serotype data or circumstantial epidemiological links. Furthermore, we will define an outbreak as an event where two or more human cases are presumably linked to the same source. We acknowledge that the epidemiological definition of an outbreak differs from this definition, but due to the large amount of underreporting and the problem of attributing cases with broad host-range serotypes to animal sources we chose this definition.

As some serotypes are strongly associated with specific animal species and some case definitions utilize serotype data to define for instance reptile-acquired cases (see for instance [31,32]), serotype-dependent differences in case detection are likely. Similarly, unusual exposures, such as those attributable to newly acquired or exotic pets, visits to animal exhibits, or contacts with clinically sick animals, are more likely to be recalled than routine exposures (see for instance [33-36]). In addition, small outbreaks are probably less likely to be reported in the peer-reviewed literature. Animal acquired infections are therefore probably strongly underreported, and the data is potentially biased towards larger outbreaks, uncommon serotypes, certain animal species, and unusual exposures.

## 3. Mammals as Source of Human Infection

### 3.1. Mammalian livestock species and *Salmonella*

#### 3.1.1. The global distribution and economic importance of mammalian livestock

The estimated number of mammals farmed for agricultural purposes around the world exceeds 4 billion animals [37]. In 2006, approx. 20% of the world's population, equaling approx. 1.3 of 6.55 billion people, were employed in the livestock sector, and livestock accounted for approx. 40% of global agricultural output [38]. An estimated 1 billion pigs and 2 billion small ruminants are farmed worldwide [39]. The global cattle population is believed to equal approx. 1.3 billion animals, and 181 million buffaloes as well as approx. 24.7 million camels are farmed for commercial purposes around the world [37]. The relative and absolute abundance of livestock differs considerably by country and geographic area. For instance, the USA is the world's largest producer of beef and the third-largest producer of pork, while large numbers of cattle and pigs are also farmed in the EU [37]. Australia is a leading producer of wool and sheep meat, and India is an important producer of goat, buffalo and cow milk and a leading producer of goat and buffalo meat. Africa as well as parts of Western Asia are major producers of camel and goat products [37].

#### 3.1.2. Cattle and *Salmonella*

##### 3.1.2.1. The clinical and economic importance of *Salmonella *infections among cattle

Abortions attributable to *Salmonella *infection are possible but rare in cattle, thus economic losses in cattle operations are primarily due to increased mortality, performance losses, and direct and indirect costs associated with treatment and infection control (see [40] for a review of the topic). Mortality rates attributable to *Salmonella *infection are particularly high in young animals, which also generally require the greatest amount of treatment. Significant weight losses in calves due to *Salmonella *infection have been reported in numerous studies, even though surviving calves seem to regain the weight after recovery (see [40]). In addition, *Salmonella *infection often leads to increased feed costs due to diminished feed conversion [40].

Clinically, *Salmonella *infection in cattle is typically manifested as watery or bloody diarrhea, and often associated with fever, depression, anorexia, dehydration and endotoxemia. Less common clinical manifestations include abortion and respiratory disease, and mortality rates can be high. Particularly in adult animals *Salmonella *frequently causes subclinical disease, and is known to persist on infected farms for months or years [41-44]. Individual animals shed *Salmonella *intermittently, over variable periods of time, and infections with host adapted serotypes such as *Salmonella *Dublin may potentially result more frequently in the development of asymptomatic shedders than infections with broad host-range serotypes [45]. One recent study estimated the median duration of shedding in dairy cattle to equal 50 days, with a maximum duration of 391 days, and the results appear comparable to previous reports [46,47]. However, the duration of *Salmonella *persistence in herds exceeds maximum shedding durations observed for individual animals, and is believed to be largely attributable to endemic *Salmonella *infections within the herd [47]. Several studies report isolating *Salmonella *at high rates from farm environments, a likely important *Salmonella *reservoir [43,47-50].

*Salmonella *within- and between-herd prevalence estimates vary considerably, with between-herd point prevalence estimates for cattle operations ranging from 2-42% and within-herd estimates for these operations ranging from 0-37% [47,51-57]. In addition, herds with clinically sick animals are generally characterized by higher within-herd prevalence than herds where clinical salmonellosis is absent, and serotype distribution may differ between herds with and without clinical cases [58-63]. Large herd size represents an important risk factor for salmonellosis, and the risk of *Salmonella *shedding seems to vary by production system, housing type, general hygiene level, management type and animal age, although the results reported in the literature have been somewhat contradictory [64]. Calves, heifers, and periparturient cows generally appear to be at a particular risk of infection, and one study found heifers and periparturient cows to be the most likely cattle to become asymptomatic carriers [47,65,66].

The distribution of *Salmonella *serotypes among cattle varies greatly over time, and differs among geographic regions, age groups, clinical manifestation, and production systems. The United States National Veterinary Service Laboratory (NVSL), for instance, reported that serotypes Typhimurium, Newport, Orion, Montevideo, and Agona were the serotypes most frequently isolated from clinically sick cattle in 2005 and 2006, while serotypes Cerro, Kentucky, Anatum, Newport, Montevideo, and Orion were the serotypes most frequently isolated from clinically healthy cattle in the same time period [67]. In 2007, serotypes Cerro, Kentucky, Montevideo, Muenster, Meleagridis, Mbandaka and Newport were the serotypes most commonly isolated from healthy dairy cattle in the USA, while serotypes Montevideo, Meleagridis, Cerro, Mbandaka, Typhimurium, Anatum, Give, Kentucky, Muenchen and Senftenberg had been the serotypes most commonly isolated from healthy USA dairy cattle in 1996 [68]. In comparison, serotypes Montevideo, I 6,7:k:-, Braenderup, Meleagridis, Newport and I 3,10:-:1, were the serotypes most commonly isolated from USA beef cattle in 2007/2008, and serotypes Typhimurium, Anatum, Dublin, Montevideo, and Newport were the serotypes most commonly isolated from USA beef herds in 1999 [69,70]. The implications of these differing serotype distributions for human health, however, are currently difficult to assess.

##### 3.1.2.2 The public health importance of *Salmonella *infection among cattle

Cattle play a paramount role as source of foodborne infection, and a considerable number of serotypes frequently isolated from humans have been isolated from sick or clinically healthy cattle (Additional file 1: Table S1). Some human cases have also been linked to direct cattle exposure (Table 1). For instance, in 2002 and 2004, human *Salmonella *Newport outbreaks in Michigan were linked to cattle contact in a public setting; in 2001, *Salmonella *Newport was transmitted to humans during a farm visit, even though the consumption of contaminated raw milk may have been a contributing factor; and in 2000, serotype Typhimurium was transmitted to children at a farm camp. In several instances, attribution of human cases to cattle exposure is further complicated by simultaneous consumption of raw milk or cheese from the same farm, as illustrated by recent outbreaks of *Salmonella *Newport and Dublin (Table 1). Nail biting, contact with manure, thumb sucking, eating, or having soiled hands and shoes have been identified as risk factors for animal-acquired *E. coli *infections, and a similar role for *Salmonella *appears likely [71]. Much less is known about the *Salmonella *risk posed to humans by indirect animal contacts, especially through environmental contamination. Further studies, especially on spatial clustering of human cases around livestock premises, are needed to assess the indirect risks posed by livestock operations.

**Table 1:** Documented reports of *Salmonella *transmissions from mammals to humans available in the peer-reviewed literature or otherwise published by public health agencies

Outbreak source	Year	*Salmonella *serotype	Type of contact	Human cases	Country	Reference
**Livestock**						
Cattle	2005^a^	Stanley	occupational (dead calf delivery); pustular dermatitis	1	UK	[36]
Cattle	2004	Newport	public setting	6	USA	[35]
Cattle	2003	Newport	public setting	3	USA	[35]
Cattle	2002	Newport	public setting	6	USA	[35]
Cattle	2001	Newport	farm visit; potentially raw milk consumption	4	USA	[35]
Cattle	2000	Typhimurium	farm day camp	1	USA	[35,304]
Cattle	1998	Typhimurium	household or farm environment	1	USA	[305]
Cattle	1990	Virchow	occupational (dead calf delivery); dermatitis	2	Netherlands	[306]
Cattle	1983	Newport	farm environment, nocosomial, feed-borne	1	USA	[307]
Cattle	1979	Dublin	farm environment, nocosomial, potentially raw milk	n/a	USA	[307]
Cattle	1976	Heidelberg	farm environment, secondary perinatal &nocosomial	n/a	USA	[307]
Cattle	1975	Dublin	occupational (dead calf delivery); pustular dermatitis; 3 cases	3	UK	[308]
Cattle	1973	Saintpaul	occupational (dead calf delivery); folliculitis	3	Canada	[309]
Cattle	1973	Typhimurium	farm environment, animal feed pot. source	n/a	USA	[307]
Cattle	1972	Typhimurium	occupational, farm environment	n/a	USA	[307]
Cattle	1969	Dublin	occupational (dead calf delivery); pustular dermatitis	1	UK	[308]
Cattle	1965	Typhimurium	farm environment, cow and newborn calf	2	Canada	[310]
Cattle	1948	Typhimurium	farm environment, household, well water	7	Canada	[311]
Cattle/Pigs	2001	Typhimurium	farm or household (contaminated clothes)	1	Netherlands	[37]
Pigs	2005	Typhimurium	public setting; potentially environmental	19	USA	[35]
Sheep	1998-2003^b^	Brandenburg	occupational, household, prob. secondary dogs	n/a	New Zealand	[97]
Sheep/Cattle	1991-1993	Typhimurium	occupational, household, farm environment	9	UK	[73]
Sheep	1975	Typhimurium	occupational, farm environment, secondary dog infected	1	UK	[72]
Livestock	2000	Typhimurium	petting zoo, animal source unclear	18	US	[303]
Livestock	1991	Typhimurium	science fair, animal source unclear	5	US	[303]
						
**Equines**						
Horse	2001	Newport	state fair, horse clinically sick	2	USA	[35]
Horse	1995/1996	Typhimurium	occupational, veterinary hospital, secondary ruminants	2	USA	[145,312]
Horse	1976	Typhimurium	occupational, veterinary hospital, secondary dog	1	USA	[313]
Horse	1967/1968	Typhimurium	occupational, veterinary hospital, complex epidemiology	2 - 14*	UK	[314]
Horse	1936	Abortusequi	occupational, gynecological exam, developed abscess	1	Japan	[315]
						
**Canines & Felines**						
Cat	1999	Typhimurium	occupational, veterinary clinic	10	USA	[316]
Cat	1999	Typhimurium	household, secondary daycare contact, shelter cats	7	USA	[316]
Cat	1999	Typhimurium	occupational, veterinary clinic, secondary environmental	3	USA	[316]
Cat/Dog	2003	Typhimurium	occupational, veterinary clinic, household infections	7	USA	[317]
Cat/wild birds	1999	Typhimurium	household, prob. complex transmissions	n/a	Sweden	[318]
Cat/Dog	1973	Typhimurium	household, dog and cat breeder, common food source	4	Canada	[319]
Dog	1974	Enteritidis	household	1	USA	[148]
Dog	1952	Paratyphi B	household	1	UK	[320]
Dog	1938	Glostrup	household, pot. common food source	6	Denmark	[321,322]
Dog	1937	Paratyphi B^1^	household	6	Norway	[322,323]
Dog	1938	Paratyphi B	household, caused abortion in bitch	4	Sweden	[322,324]
**Pet food & Treats**						
Dry pet food	2006-2008	Schwarzengrund	household	70	USA	[325]
Pet treats	2004/2005	Thompson	household	9	USA & Canada	[326]
Pet treats	2002	Newport	household	5	Canada	[327]
Pet treats	1999	Infantis	household, dogs potential shedders	12	Canada	[328]
						
**Rodents**						
Guinea pig	2000	Oranienburg	household, guinea pig soft-tissue abscess and died	1	USA	[329]
Guinea pig	1967	Enteritidis	breeding colony in household	3	Canada	[330]
Rodents	2005/2006	Typhimurium	classroom or household, snakes fed frozen rodents	7 - 21*	USA	[331]
Rodents	2003/2004	Typhimurium	household, sick pet rodents, secondary household	15 - 28*	USA	[332]
						
**Non-traditional mammalian pets/wildlife**						
Hedgehogs	2002	Typhimurium	household, potentially eggs	6	Australia	[333]
Hedgehogs	2000/2001	Typhimurium	unclear, wild animals, potentially contaminated produce	37	Norway	[192]
Hedgehogs	1996	Typhimurium	unclear, wild animals, potentially contaminated produce, 2 outbreaks	28 - 65*	Norway	[192]
Hedgehogs	1995-1997	Tiliene	household, pet hedgehogs, multiple outbreaks, interspecies transmission	9	Canada	[334]
Hedgehogs	1994/1995^c^	Typhimurium	household, pet hedgehog	1	Canada	[196]
Hedgehogs	1994	Tiliene	household, pet hedgehog, indirect contact, breeding herd in household	1	USA	[335]
Sugar glider	1995	Tiliene	household	1	Canada	[334]
Wallaby	2003	Enteritidis	farm environment, traveling petting zoo	17	USA	[35]

^1 ^identical to serotype Abortuscanis

^a ^estimated time of outbreak, exact time not specified; ^b ^estimated time period for prolonged nationwide outbreak; ^c ^exact time of outbreak not specified more precisely; * exact number of cases unclear; n/a exact number of cases not specified

In conclusion, the studies summarized above show that direct cattle contacts represent a potential human health risk. Clinically sick animals probably pose the greatest risk to humans because they are more likely to shed *Salmonella*, and at higher concentration, than apparently healthy animals. However, even asymptomatic carriers can shed *Salmonella *for long periods of time, and increased stress, as often experienced during exhibitions, represents an important risk factor for shedding. In addition, herds with clinical signs of *Salmonella *have higher within-herd prevalence than those without clinical signs, *Salmonella *prevalence is correlated with animal age as well as management-related factors, and environmental contamination can play an important epidemiologic role. Clinically affected herds and certain management systems may therefore pose an increased risk to the public. Several additional management practices may mitigate the human health risk associated with cattle contacts, for instance strict enforcement of good hygiene practices, the prevention of contact with manure, or targeted education of vulnerable human subpopulations. Several *Salmonella *infections have been attributed to occupational livestock contact (see for instance [36,72,73]). Surprisingly, a considerable number of cutaneous infections among veterinarians have been reported as results of obstetric manipulations, reinforcing the need for good hygiene practices and adequate protective equipment (Table 1). However, quantitative estimates of the occupational risks are scarce. One study reported *Salmonella*-specific antibodies in 60% of poultry workers and nearly 10% of workers in meat-packaging plants in Russia, with highest prevalence among those handling sick poultry or pathological material (or consuming raw meat sausages) [74]. Similarly, occupational transmission of *Salmonella *Typhimurium to a slaughterhouse employee has been reported by Molbak et al. [75]. Another study found nearly 9% of poultry workers and 6% of duck workers were *Salmonella *carriers, with intermittent clinical symptoms [76]. Conversely, another study of *Salmonella *Muenster reported no occupational transmission in an affected dairy herd, but the study relied exclusively on self-reporting of clinical disease in farm personnel, and sample size as well as observational period were limited [77]. Future studies are therefore clearly needed to understand the magnitude and specific nature of the risks associated with occupational exposure.

#### 3.1.3. Small ruminants and *Salmonella*

##### 3.1.3.1. The clinical and economic importance of *Salmonella *infections among small ruminants

The severity and clinical manifestation of *Salmonella *infection in small ruminants differs by age group and serotype [78]. Acute enteric salmonellosis is common in adult sheep, leading to fever, anorexia, depression, and diarrhea, while septicemia is common in young animals [79,80]. However, asymptomatic carriage, chronic gastro-enteritis, and abortion have also been described [80,81]. Late term abortion, mortality in ewes, and high calf mortality can lead to extensive economic losses in sheep operations, making *Salmonella *abortion one of the economically most important diseases of small ruminants [40]. Abortion due to infection with serotypes such as Typhimurium or Dublin has been reported, but abortion is most frequently caused by *Salmonella *Abortusovis, an ovine-adapted serotype that also occasionally infects goats, and abortion generally occurs in the last weeks before parturition [78,82-84]. Infections of ewes with serotype Abortusovis can also lead to stillbirth, metritis, placental retention, or peritonitis, and infected ewes may present with fever, anorexia, and depression prior to abortion [85]. Mortality rates in ewes are highly variable, and mortality is often associated with the occurrence of secondary diseases such as placental retention. Neonatal mortality in affected herds is usually high. Lambs carried to term frequently die of septicemia [78]. While neonates often die within hours of birth, lambs can in some cases survive for weeks, and in these instances disease is often manifested as polyarthritis, pneumonia, and severe diarrhea. *Salmonell*a Abortusovis infection in non-pregnant ewes and rams appears to be predominantly asymptomatic and venereal infection have been described in some instances [86].

The prevalence of *Salmonella *among small ruminants seems to vary considerably between serotypes, herds, and geographic regions. Large outbreaks of *Salmonella *Abortusovis among sheep have been reported repeatedly, for instance in Switzerland, where infections with serotype Abortusovis seem to have contributed up to 70% of lambing losses between 2003 and 2007 [87]. The prevalence of serotype Abortusovis within herds can be high, and within-herd prevalence estimates of between 20 and 50% have been reported [82,87]. Animals that survive infection with *Salmonella *Abortusovis usually develop robust immunity, so that infections tend to be associated with primigravide animals [82]. *Salmonella *Diarizonae is the causative agent of winter dysentery, a disease of sheep that is also associated with abortion and stillbirth. Serotype Diarizonae represents another common, sheep-adapted serotype. The prevalence of serotype Diarizonae in Norwegian sheep herds, for instance, has been estimated at approx. 12%, with within-herd prevalence in the range of 0-45%, even though the samples were collected at the abattoir and increased stress may have contributed to the high observed prevalence [88]. Other studies have also reported a high prevalence of various *Salmonella *serotypes including for instance serotypes Typhimurium, Anatum, or Saintpaul, in goats and sheep at slaughterhouses in different countries, with prevalence estimates generally in the range of 17-60%, even though a considerably lower prevalence among slaughtered goats and sheep in India and Ethiopia has been reported in some studies [81,89-93]. The prevalence of *Salmonella *among healthy goats and sheep on farms generally appears to be considerably lower, with reported prevalence estimates often in the range of 0-4% [94,95]. However, environmental contamination on farms is potentially high, and Edrington et al. [96], for instance, reported isolating *Salmonella *from 50% of wool samples but only 7% of fecal samples collected from feedlot sheep in the USA, indicating a potentially important epidemiological role of environmental reservoirs.

#### 3.3.2. The human public health importance of *Salmonella *infections among small ruminants

A limited number of zoonotic transmissions from sheep to humans have been reported in the literature, mostly associated with occupational exposures (Table 1). For instance, occupational sheep exposure was found to be significantly associated with human *Salmonella *Brandenburg infections in New Zealand [97]. In addition, human outbreaks have repeatedly been linked to occupational contacts on farms in the UK, reiterating the potential public health importance of sheep and goat contacts [73].

In conclusion, *Salmonella *infections represent an economic and potential public health risk on sheep and goat farms, but considerable differences between the serotypes exist. Infections with serotype Abortusovis are responsible for large economic losses, but carry little health hazards for humans. However, several other serotypes such as Typhimurium can cause similar clinical disease in ewes, with potentially important implications for human health. Good hygiene practices and personal protective clothing are crucial to prevent occupational infections, especially during lambing or obstetrical intervention. Secondary transmissions to family members after occupational exposure have also been documented, reinforcing the importance of good hygiene practices on farms to reduce the human health risk [73]. Animals with clinical signs of gastro-intestinal disease or septicemia may pose the highest risk for humans, but asymptomatic shedding at relatively high prevalence has been documented at slaughter, indicating that clinically healthy animals may also pose a considerable risk. *Salmonella *carriage among healthy animals on farms appears to be relatively rare, but environmental contamination likely contributes to the infection risk. In conclusion, contacts with small ruminants pose a potential health risk to occupationally exposed subpopulations as well as the general public, but the risk depends strongly on the serotype involved.

### 3.4. *Salmonella *and pigs

#### 3.4.1. The clinical and economic importance of *Salmonella *infections among pigs

A variety of clinical manifestations have been observed in *Salmonella *infected pigs, ranging from asymptomatic to peracute disease. Infections with generalist serotypes such as Typhimurium usually cause mild or no disease, and infected animals may shed *Salmonella *for considerable periods of time. For instance, piglets experimentally inoculated with *Salmonella *Typhimurium developed mild gastro-intestinal disease and were found to shed bacteria in their feces for several days [98]; however, systemic disease and mortality associated with broad host-range serotypes has also been reported [99]. In contrast, infection with host adapted serotype Choleraesuis generally causes severe systemic disease with high mortality (see [99] for a recent review). All age groups are susceptible to *Salmonella *infection, but disease is most commonly observed among weaned pigs more than eight weeks of age, and asymptomatic carriers are thought to represent the most important source of *Salmonella *introduction onto pig farms. A variety of clinical manifestations have been documented among *Salmonella*-infected pigs, including enteritis, septicemia, pneumonia, meningitis, and arthritis. Fever, diarrhea, inappetence, depression, respiratory distress, lameness, edema, and hypoxia in the extremities are common symptoms in clinically sick pigs, and mortality rates in such instances are high. Schofield [100], for instance, reported salmonellosis outbreaks among pigs manifested as ataxia, fever, depression, diarrhea, and necrotic enteritis, which resulted in approx. 17% mortality.

**Figure 1:**
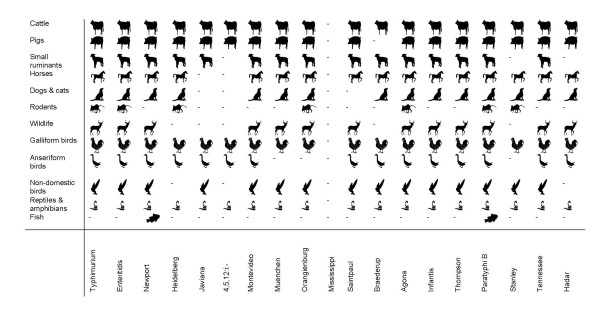
**Distribution of the 20 most common human *Salmonella *serotypes **[7]**among animals, based on US data from 2006**. *Salmonella *Typhi was excluded from this analysis as it represents a host-restricted serotype adapted to humans and non-human primates.

*Salmonella *prevalence estimates for pig farms seem to differ considerably by production and management type, with average between-herd estimates in the USA equaling 53% in 2006 and exceeding 80% for some farrow-to-finish production systems, while within-herd estimates range from 3.5 to 28% [58-63]. High *Salmonella *prevalence on pig breeding farms and considerably lower prevalence on replacement gilt development farms have been described in one study [58]. However, another study reported high prevalence among replacement gilts and finishing gilts, suggesting variability between herds and studies [60]. Surprisingly, Davies et al. [101] reported a higher *Salmonella *prevalence in all-in-all-out than continuous flow management systems, and distinct *Salmonella *serotype populations in breeding herds, nursery and finishing herds from the same farrow-to-finish system have been reported [101]. Breeding herds or nurseries therefore seem to represent epidemiologically relatively unimportant sources of infection in finishing herds, and environmental contamination may play an important role in maintaining endemic infections. In fact, Dahl et al. [102] demonstrated that *Salmonella *free finishing herds can be produced from endemically infected herds if pigs are strategically moved to clean stalls as they move through the farrow-to-finish system. Reducing the prevalence of *Salmonella *is particularly important because *Salmonella *prevalence at slaughter tends to be considerably higher than on farm [64]. Indeed, one study reported 7-fold higher *Salmonella *prevalence in pigs sampled at the abattoir than in animals from the same herds sampled on farm, indicating an important effect of stress or other transportation-related factors [103]. In addition to host adapted serotype Choleraesuis, serotypes Typhimurium, Derby, Agona and Anatum are frequently isolated from sick and clinically healthy pigs, indicating a potential risk for human health (Figure 1, Additional file 1: Table S1).

##### 3.1.4.2. The human public health importance of *Salmonella *infections among pigs

On few occasions, likely zoonotic transmissions of *Salmonella *from pigs to humans have been described (Table 1). For instance, in 2005, a Typhimurium outbreak among humans in Wisconsin was linked to indirect pig contact in a public setting [34]. Similarly, in 2001 occupational exposure to pigs likely led to human infection, even though in this case the possibility of a transmission from calves could not be conclusively eliminated [36].

In conclusion, *Salmonella *represents an occupational hazard for those working with pigs, especially since asymptomatic carriage of broad host-range serotypes appears to be relatively common. Environmental reservoirs appear to play an important role in maintaining endemic infections, and contaminated clothing has been implicated in the transmission of *Salmonella *from pigs or calves to the son of a farmer, indicating the paramount importance of good hygiene practices [36]. Stress probably represents a major reason for the increased *Salmonella *prevalence among pigs at slaughter relative to that observed on farms, and a similarly increased prevalence of shedding during exhibitions or at other public venues appears likely. Contact with pigs on farms, at the slaughterhouse, or in the scope of public exhibitions therefore likely represents a risk to occupationally exposed population subgroups and the general population.

### 3.2. Companion animals and *Salmonella*

#### 3.2.1. The changing role of companion animals in the 20^th ^century

The keeping of animals as pets has a long tradition, but historically, companion animals were foremost held for labor [104,105]. Dogs served as guard dogs, hunting companions, or were used for herding, while cats were kept to catch rodents. Dogs and cats were rarely kept in homes, even pet animals [104]. During the 19^th ^century engine-powered machines replaced horses as sources of labor. Simultaneously, the public's attitude towards animals changed, manifested in the foundation of animal welfare organizations such as the British Royal Society for the Prevention of Cruelty to Animals (RSPCA) in 1824 [104,105]. The number of pet animals held for companionship increased drastically after World War II [104]. Currently, an estimated 63% of households in the USA own at least one pet; approximately 83.2 million households own dogs or cats, and roughly 4.3 million households own horses [106]. Similarly, in the United Kingdom, an estimated 26% of households own cats and 31% of households own dogs, amounting to approximately 10.3 million cats and 10.5 million dogs [85]. Today, dogs and cats primarily live indoors, share living spaces with their owners, and assume integral roles as companions, family members, or service animals [104,107]. In one recent survey 95% of USA dog owners reported petting their animals, 67% reported playing with them, and 30% reported sharing their beds with their dogs [108]. Companion animals are also increasingly used in therapeutic settings, for instance in psychotherapy, or to support AIDS patients, children with disabilities, orthopedic and cardiac patients, Alzheimer patients, or the elderly [109-114]. The potential risks associated with such contacts, particularly for young children or immune-compromised patients, are difficult to quantify. In some parts of the world, companion animals still fulfill functional roles as source of food or labor, and may be allowed to roam around freely [115].

#### 3.2.2. *Salmonella *infections in horses and humans

##### 3.2.2.1. The clinical and economic importance of *Salmonella *infections among horses

Salmonellosis is an important disease of horses. Equine mortality rates vary depending on host age, predisposing factors and potentially the *Salmonella *serotype involved [116]. Mortalities as high as 40 to 60% have been reported, but in general, mortality appears to be considerably lower [117,118]. In most cases, animals present with profuse, watery and malodorous diarrhea, frequently associated with abdominal pain and endotoxemia. Fever, dehydration and depression are common, and in severe cases these symptoms are accompanied by colic, gastric reflux, cardiovascular shock or coagulopathies. However, the severity of disease can vary considerably and, in animals of the same age group, may range from severe to asymptomatic [119]. Both peracute and chronic forms of disease are common, and convalescent carriers may shed *Salmonella *for months, but a carrier state does not appear to develop in all instances [118,120,121]. Disease may also manifest without gastrointestinal signs. Some serotypes appear to result more frequently in systemic disease than others, but the underlying mechanisms are still incompletely understood [122]. Respiratory forms are comparably frequent, and systemic forms of infection are commonly associated with arthritis, osteomyelitis, or soft-tissue abscesses [123,124]. Foals, pregnant mares, and immune compromised horses are at a heightened risk of infection and, among foals, *Salmonella*-associated meningoencephalitis has been described [125,126]. Abortions due to *Salmonella *cause important economic losses on stud farms [127-129].

Numerous studies have focused on horses in equine hospitals, with apparent prevalence estimates ranging from 1.8 to 18%; Anderson and Lee, however, report isolating *Salmonella *from 26.6% of slaughter horses [125,130-135]. The prevalence among healthy horses on farms or in riding schools appears to be considerably lower, in the range of 1 to 2% [117,131,135,136]. Asymptomatic carriers shed *Salmonella *intermittently. Increased shedding has been associated with antibiotic treatment and stressful situations such as transportation, horse competitions, co-morbid disease, or surgery [125,133,137-140]. High population density is thought to be another predisposing factor. The epidemiological significance of environmental contamination remains difficult to assess, but good environmental hygiene practices have been efficient in controlling hospital outbreaks [132,141-143]. Numerous serotypes have been isolated from clinically healthy or sick horses, and a considerable number of outbreaks involving a variety of medically important serotypes have occurred in large animal hospitals (Table 1, Additional file 1: Table S1).

##### 3.2.2.2. The public health importance of *Salmonella *infection among horses

Zoonotic transmission in large animal veterinary hospitals and private veterinary clinics is thought to occur frequently, even though only a small number of human cases associated with such transmissions have been documented (Table 1) [144,145]. *Salmonella *transmission to humans at a state fair has also been reported (Table 1) [34].

In conclusion, horse contacts clearly represent a risk to humans. However, clinically healthy horses in riding schools or on farms, especially if held under optimal conditions, seem to pose a comparably low risk. The risk at competitions, state fairs or other public venues might be considerably higher due to increased stress, whereas pregnant mares, foals and hospitalized horses clearly represent a high risk. *Salmonella*-infected horses often present with no or atypical clinical symptoms, emphasizing the need for strict quarantine, environmental contamination control, and good hygiene practices. Due to the high *Salmonella *prevalence, high risk population subgroups may choose to refrain from entering equine hospitals or stud farms, or take particular precautionary measures.

#### 3.2.3. *Salmonella *infections in dogs, cats and humans

##### 3.2.3.1. The veterinary importance of *Salmonella *infections among dogs and cats

A considerable number of *Salmonella *serotypes have been isolated from domestic dogs and cats around the world (Figure 1, Additional file 1: Table S1). The majority of infections are asymptomatic [146]. However, gastrointestinal disease manifested as enterocolitis and endotoxemia can occur and is often associated with fever, vomiting, anorexia, dehydration and depression [147-149]. Abortion, stillbirth, meningoencephalitis, respiratory distress and conjunctivitis have also been described [150,151]. *Salmonella *prevalence among dogs and cats appears variable and probably depends on a variety of factors. One study analyzed the apparent *Salmonella *prevalence among greyhounds on race tracks and found 43.5% of dogs were shedders, while another study described the apparent prevalence among racing Alaskan sled dogs at approx. 60%, and the prevalence among stray dogs is likely equally high [147,152-154]. In general, however, the rate of shedding is thought to be much lower, and a number of studies on non-racing client-owned dogs and client-owned cats report shedding rates in the range of 1-5% [155-160].

Ingestion of contaminated food is thought to be the predominant risk factor. *Salmonella *has been isolated at high frequency from raw dog food on greyhound race tracks, and asymptomatic carriers developed after experimental oral inoculation, with shedding observed for periods of up to four weeks [161,162]. Dogs fed raw food diets appear to be at particular risk. Finley et al. [163] report that, in the absence of clinical signs, 50% of dogs fed contaminated raw food diets shed *Salmonella *in their feces, while none of the control dogs fed *Salmonella*-free diets shed *Salmonella*. In another, longitudinal study, Joffe et al. [164] isolated *Salmonella *at least once from the feces of 80% of client-owned dogs fed a common bone and raw food (BARF) diet. Surprisingly, *Salmonella *was also isolated on one or more occasions from 30% of client-owned controls, which were fed commercial dog food. Some serotypes such as *Salmonella *Typhimurium, Heidelberg, and Kentucky appear to be predominantly isolated from dogs fed raw food diet, and one study estimated the odds of shedding *Salmonella *to be approx. 23 times greater for dogs fed raw food diets than commercial diets [165]. Asymptomatic carriers shed *Salmonella *intermittently, and longitudinal studies provide evidence for multiple coinfections during relatively short time periods [166].

##### 3.2.3.2. The public health risk associated with *Salmonella *infection among dogs and cats

A number of medically important serotypes for humans have been isolated from domestic dogs and cats (Figure 1, Additional file 1: Table S1), and several studies have reported the isolation of multidrug-resistant isolates (see [167] for a comprehensive review of the topic). Human *Salmonella *cases have been attributed to contact with infected dogs or cats at home or in veterinary clinics (Table 1). For instance, in 1938 the family dog was implicated as source of a human *Salmonella *Glostrup outbreak and in 1952 a human case of Paratyphi B was linked to direct dog contacts at home. In 1999 human outbreaks of *Salmonella *Typhimurium in Idaho and Washington were linked to contact with clinically ill kittens in veterinary clinics, and a human outbreak in Minnesota was linked to contact with cats from a shelter. A 2003 outbreak of *Salmonella *Typhimurium among humans in New York was also linked to a small animal veterinary clinic, but the index animal was not clearly identified. A recent case-control study of childhood salmonellosis in Michigan identified cat exposure, as well as reptile contacts, as risk factor for *Salmonella *infection, emphasizing the potential risk [168]. The risk posed to humans by indirect contact, for instance with excrement, is currently not clear. Infected humans also represent a possible source of infection for their animals. In addition, animal-to-animal spread occurs readily and has been clearly documented during a *Salmonella *outbreak in a military dog kennel [169]. In this instance, the index case acquired *Salmonella *through feed.

Together, these data indicate that contacts with dogs and cats in homes, veterinary clinics and shelters clearly represent potential threats to human health. Raw food diets are associated with a significantly higher prevalence of *Salmonella *than other pet food diets, and since asymptomatic shedding is common it appears that animals on such diets might be suspected of *Salmonella *shedding regardless of clinical symptoms. Especially if some household members are at a heightened risk of infection, or if animals are introduce into therapeutic settings, other feed types may be preferable. *Salmonella *represents a clear occupational hazard. Good hygiene practices, environmental infection control, strict quarantine procedures, personal protective equipment, and other biosecurity measures are therefore crucial to reduce the risk wherever dogs or cats are kept in large groups or subjected to high levels of stress.

#### 3.2.4. Commercial pet food and treats as sources of infection

*Salmonella *contamination in pet foods and treats varies considerably by food type. Commercial raw food diets, representing combinations of raw meat, vegetables, grain and eggs or fruit, are available fresh or frozen in a large number of pet stores and veterinary clinics, and a recent Canadian study estimated *Salmonella *prevalence in these feeds to equal approx. 21% [170]. Several *Salmonella-*related recalls of raw foods have been reported in recent years, for instance of frozen cat food in the USA in 2007. Contamination rates in dry or canned foods are thought to be considerably lower, and to our knowledge *Salmonella *has not been isolated from canned dog food, but the number of available studies is very limited [8,9]. Dry dog food has recently been linked to a large human salmonellosis outbreak, and dog and cat vitamins have been recalled due to *Salmonella *contamination, but prevalence data is currently scarce [9,171]. Dried pigs ears and a variety of other dried animal parts are commercially available as dog treats. Contamination rates in these commodities appear to be high. For instance, in 2001 a Canadian study reported isolating *Salmonella *from 50% of pig ears and other animal-derived pet treats, and a 2003 study found 41% of animal derived pet treats sold commercially in the USA contaminated with *Salmonella *[172,173]. Isolates included *Salmonella *Typhimurium, Heidelberg, Anatum, Infantis, and Derby, and a considerable number of samples contained more than one serotype (Additional file 1: Table S1). Voluntary preventive measures implemented by the pet treat industry appear to have led to a considerable reduction in contamination rates, but a disease risk remains [174]. For instance, in December 2009, pig ears and beef hoof products were recalled in the USA because of a potential *Salmonella *contamination.

Exposure to commercial pet food and animal derived pet treats has also repeatedly led to human outbreaks (Table 1). For instance, in 2007 the CDC identified a multi-state outbreak of *Salmonella *Schwarzengrund linked to commercial dry pet foods, which affected dogs and their owners in 18 states of the USA and led to a nation-wide product recall. In 1999, pig ear treats contaminated with *Salmonella *Infantis led to a human outbreak of salmonellosis in Canada, and in 2002, pet treats contaminated with *Salmonella *Newport were responsible for human *Salmonella *infections in Canada. *Salmonella *outbreaks among humans have also been linked to rodents commercially sold as pet food, but these are more appropriately described in the section regarding rodents.

In conclusion, pet feeds represent a direct and indirect threat to human and animal health. The choice of pet food and treats can considerably influence the *Salmonella *risk for animals and humans, and might be of particular importance if high-risk human population subgroups are exposed at home or in therapy settings, or if animals are exposed to stressful situation such as in kennels, veterinary clinics or shelters. However, good hygiene practices such as hand washing before and after feeding, appropriate cleaning of bowls and contact surfaces, and adequate storage can probably decrease the direct risk for humans considerably.

### 3.3. Rodents, rabbits and *Salmonella*

#### 3.3.1. The clinical and environmental importance of *Salmonella *infections among rodents and rabbits

*Salmonella *has repeatedly been isolated from wild mice and rats, which represent important reservoir hosts on farms and in food production environments (Additional file 1: Table S1). Prevalence estimates for wild or captive rodents are relatively scarce, variable among geographic regions, and the numbers of studies as well as the prevalence seem to have decreased over time. In general, *Salmonella *shedding rate estimates are in the range of 1 to 15% [175-179]. *Salmonella *prevalence among captive rodents is low, and environmental reservoirs may play a paramount epidemiologic role [180]. *Salmonella *has also been isolated from pet rabbits, indicating a potential risk associated with this animal species [181]. However, the available prevalence data, especially for pet rabbits, is currently very scarce. *Salmonella *might to be fairly common in intensive rabbit meat production systems, with one study reporting that as many as 30% of intensive rabbit farms in Italy were positive for *Salmonella *[182]. However, a low prevalence of *Salmonella *among rabbit carcasses in Spanish slaughterhouses has also been reported, indicating that *Salmonella *prevalence among commercially farmed rabbits is probably variable [183].

*Salmonella *infection can cause severe disease in rabbits, which is sometimes associated with high mortality [182]. Clinical symptoms include enteritis, metritis and abortion, but striking differences in pathogenic potential seem to exist among different *Salmonella *serotypes [182]. In contrast, the majority of infections in mice and rats are asymptomatic. However, clinical disease among rodents has also been described, for instance during large outbreaks among laboratory rodents, which were associated with high mortality rates (Additional file 1: Table S1). Indeed, *Salmonella *Typhimurium and Enteritidis have been widely used as rodenticide in the first half of the 20^th ^century and continue to be used in some countries despite the public health risk [184,185]. Systemic disease appears to be the most common clinical manifestation in mice and rats, and mortality is predominantly attributable to septicemia [186]. Pathogenicity is age and host strain dependent, with the highest mortality rates observed in young animals under three weeks of age [187]. A number of serotypes with importance for humans have been isolated from wild, laboratory or pet rodents (Figure 1, Additional file 1: Table S1). Clinical *Salmonella *outbreaks among guinea pigs and hamsters, associated with conjunctivitis and soft tissue abscesses, have also been described [188,189].

#### 3.3.2. The public health importance of *Salmonella *infection among rodents and rabbits

In recent years, human outbreaks have repeatedly been associated with captive rodents sold in the USA as snake feed or pets (Table 1). For instance, in 2004 a large multi-state outbreak of *Salmonella *Typhimurium among humans was associated with hamsters, rats and mice sold in pet shops across the USA. Similarly, in 2005 and 2006, a multi-state outbreak of *Salmonella *Typhimurium among humans was linked to frozen rodents sold as commercial snake feed. Sporadic human cases have also been linked to rodent contact, for instance a human case of *Salmonella *Oranienburg attributable to contact with a clinically sick guinea pig. In conclusion, rodent contacts represent a direct and indirect threat to human health. Wild rodents can serve as source of human infection by contaminating feeds, food, water or the environment, and they can conceivably infect dogs, cats or other animals if ingested. Salmonellosis represents an occupational hazard for exterminators, rodent breeders, and others that professionally handle rodents. Rodents often show no or atypical signs of salmonellosis, thus clinical symptoms in animals are of limited diagnostic value. To our knowledge, data on zoonotic transmissions from rabbits is not available at the point of writing, likely at least in part due to underreporting of human salmonellosis cases. Contacts with sick or clinically healthy rodents or rabbits can potentially lead to human exposures, and the enforcement of good hygiene practices is important to minimize the risk for humans.

### 3.4. The role of non-traditional mammalian pets and wildlife

Non-traditional pets are captive-bred or wild-caught, endogenous or exotic animals held as pets that have not reached wide-spread popularity among pet owners and are therefore not commonly bred for human companionship [190]. Apart from reptiles, amphibians, and fish, non-traditional pets include a variety of mammalian species such as non-human primates, African pygmy hedgehogs, ferrets, prairie dogs, and sugar gliders [190,191]. Lack of husbandry expertise often results in stress, malnutrition or abandonment, and bites or scratches pose a considerable risk to pet owners [191].

#### 3.4.1. Exotic pets, wildlife and *Salmonella*

Little is known about the prevalence, pathogenicity, and distribution of *Salmonella *among non-traditional mammalian pets or their wild relatives, but *Salmonella *has been isolated from a large number of wild mammals or their feces, including opossums, squirrels, woodchucks, raccoons, foxes, mink, cougars, tigers, wild boars, hippopotami, rhinoceroses, seals and whales (Figure 1, Additional file 1: Table S1). Serotypes with particular importance for human health have also been isolated from white-tailed deer feces in Nebraska, even though *Salmonella *prevalence in this species is believed to be quite low (Additional file 1: Table S1). One Norwegian study of *Salmonella *in feral hedgehogs reported prevalence estimates between 0 and 41%, which varied considerably by geographic area [192]. High prevalence corresponded to human outbreaks in the same region, and isolates with identical PFGE patterns were isolated from wild hedgehogs and humans, potentially indicating an epidemiological link, which is also supported by an independent Danish study [193]. *Salmonella *prevalence among captive hedgehogs, sugar gliders and other non-traditional pets is currently unknown. Clinical disease associated with *Salmonella *infection has been described in sugar gliders and hedgehogs, but a large number of cases are believed to be asymptomatic [194,195].

Several human cases and outbreaks of serotypes Typhimurium and Tielene have been linked to pet hedgehog contacts (Table 1), and *Salmonella *Enteritidis and Sofia have also been isolated from hedgehogs kept as pets (Additional file 1: Table S1). *Salmonella *Tielene represents a very rare serotype. Human cases are strongly associated with hedgehog exposure, and children appear to be at heightened risk of infection [195]. *Salmonella *Tielene was first isolated in the USA in 1994 during a human outbreak associated with an African pygmy hedgehog breeding colony [195]. In Canada the geographic distribution of human Tielene cases starkly resembles that of pet hedgehogs, emphasizing the epidemiological role of this pet species [196]. Human Tielene outbreaks have also been linked to sugar glider exposure, indicating a role of this exotic pet in *Salmonella *Tielene epidemiology [196].

In conclusion, wildlife and exotic pets clearly represent potential sources of human infection, but relevant data is so far scarce. *Salmonella *can cause disease in these animals, but asymptomatic carriers appear to be common and likely also pose a considerable infection risk. Good hygiene practices and measures that reduce stress, such as adequate housing, nutrition and care, can likely reduce the risks associated with captive animals.

## 4. Avian Sources of Human Salmonellosis

### 4.1. Overview of *Salmonella *infections in birds

World-wide, birds are held for meat or egg production, companionship, sports, scientific or educational purposes. In 2007, an estimated 17.9 billion chickens, 1.1 billion ducks, 447 million turkeys and 343 million geese and guinea fowl were farmed world-wide [39]. Of these, approx. 9.4 billion chickens, 997 million ducks, 14 million turkeys and 307 million geese were farmed in Asia alone. In addition, a considerable number of animals are held as pets, with 6.4 million households in the USA alone owning pet birds [106].

### 4.2. *Salmonella *infections among galliform birds

Chickens, turkeys, quails, pheasants and other gamebirds are members of the order galliformes. *Salmonella *is common in galliform birds, and has been isolated at high rates from commercially reared chicken, turkeys, and other poultry (Figure 1, Additional file 1: Table S1). Apart from the associated foodborne risk, farms may represent a direct risk to public health, even though relevant studies are so far missing and high biosecurity standards in most commercial poultry productions probably minimize the risk.

The clinical symptoms associated with *Salmonella *infection vary considerably by age group and serotype [197]. Infections with generalist serotypes rarely cause clinical disease in galliform birds and most animals become asymptomatic carriers, even though severe clinical disease with high mortality has been observed in some instances, particularly during infections of young birds [198,199]. Infections with the host adapted serotype Gallinarum biovars Gallinarum and Pullorum, however, cause severe disease with high mortality and immense economic losses on chicken and turkey farms (see [200] for a review of the topic). *Salmonella *Gallinarum biovar Pullorum causes "Pullorum disease" in young animals, which is associated with septicemia and high mortality that can exceed 85% in some instances [197]. *Salmonella *Gallinarum biovar Pullorum infections of adult birds are generally mild or asymptomatic, even though decreases in fertility and egg production as well as increased mortality have been observed in some instances. Adult animals can develop a carrier state, and transovarian transmission is thought to be the primary rout of transmission to young birds, even though rodents and other vectors are also thought to play an important epidemiologic role (see for instance [201]). Clinical symptoms include anorexia, diarrhea, dehydration, decreased hatching, and high mortality. *Salmonella *Gallinarum biovar Gallinarum causes "fowl typhoid" in young and particularly adult birds [200]. Clinical symptoms are very similar to those observed during infections with biovar Pullorum, and economic losses during outbreaks can be very high. Both Gallinarum biovars Gallinarum and Pullorum are host restricted and therefore pose a negligible risk to human health. In contrast, infections with *Salmonella *Enteritidis are typically asymptomatic in adult birds but can cause systemic disease in young birds, and transovarian transmission of serotype Enteritidis has also been described [202]. Infections with *Salmonella *Enteritidis pose a considerable human health risk, and have been estimated to inflict costs of approx. 1 billion US dollar per year on the USA economy [203].

*Salmonella *prevalence varies considerably by poultry type, differs between serotypes and biovars, and intestinal carriage often appears to be lower than isolation rates from egg shells, dead birds, and environmental samples [204,205]. *Salmonella *prevalence in hatcheries is estimated between 0 and 17% for chickens, compared to approx. 25% for geese, and 20-60% for ducks [204,205]. *Salmonella *Gallinarum biovars Pullorum and Gallinarum have been eradicated in commercial poultry productions in the developed world, but are still important in backyard flocks as well as the developing world [197]. It is conceivable that serotype Enteritidis filled the ecologic niche left by the eradication of serotype Gallinarum biovar Gallinarum, since a considerable increase in Enteritidis prevalence co-incided with the eradication of biovar Gallinarum in the 1960s (see [206] for a review of the subject). In fact, mathematical modeling results have suggested a potential role of competitive exclusion between serotypes Enteritidis and Gallinarum biovar Gallinarum in poultry [207]. *Salmonella *Enteritidis, as well as serotypes Typhimurium, Kentucky, and Heidelberg, are commonly detected among clinically healthy as well as sick chickens and turkeys, indicating a potentially important risk for human health (Figure 1, Additional file 1: Table S1).

A number of people raise chickens and other poultry in their backyards for meat, egg production or as pets [208]. In addition to household exposure, human cases have been linked to poultry contact on farms, in agricultural feed stores, and at country fairs [209]. Young hatchlings pose a particularly high risk for humans, and remarkably often infect children (Additional file 2: Table S2). The number of human outbreaks increases strikingly around Easter, when chicken or duck hatchlings are especially popular pets. Such outbreaks have been documented every few years since the 1950s (see for example [209-211]). To reduce the risk associated with hobby farming, the sale of poultry for meat or egg production at feed stores is prohibited in all USA states [212]. In addition, the USA CDC and some state health departments work to increase public awareness and to promote customer education at the store level. Some USA states have passed additional regulations, such as legislations restricting the minimum age of animals at sale or the maximum number of animals purchased per person [212]. However, enforcement of these laws is difficult and legislation varies among states [213]. The USA CDC has published recommendations regarding pet poultry, including that no children under the age of 5 handle baby poultry [214].

In conclusion, the human health hazards posed by pet poultry, and young hatchlings in particular, are substantial, but fairly well understood. The laws and recommendations provided by the USA CDC and comparable institutions can successfully mitigate the risks. However, public awareness and collaboration between governmental agencies, related industries, special interest groups, and the veterinary community is crucial to assure sustainable results. The risks of direct transmission to humans that are associated with commercial poultry productions are less well understood. *Salmonella *certainly poses an occupational risk to farmers, veterinarians and slaughterhouse employees, but whether commercial poultry farming poses direct risks to the general population remains yet to be determined.

### 4.3. *Salmonella *infections among anseriform birds

Ducks, geese and swans belong to the order Anseriformes. The majority of *Salmonella *infections in ducks appear to be asymptomatic, but severe clinical disease with high mortality has also been described [215]. Clinical disease is predominantly observed in young animals, and seems to generally be associated with environmental or management stressors. Common symptoms include anorexia, depression, diarrhea, dehydration, ataxia, opistothotonus, arthritis, and synoviatis, and decreases in fertility and hatching have also been reported [215].

The prevalence of *Salmonella *shedding appears to be species and age group dependent. *Salmonella *shedding is comparably common among commercially raised ducks and geese, yet highly variable across age groups. *Salmonella *Typhimurium, for instance, has been isolated from 40% of hatchlings and 1% of older ducklings in Taiwan, even though clear host species specific differences were also detected [216]. The prevalence of *Salmonella *shedding among wild birds appears to be quite variable (see for instance [217] for a review). Mitchell and Ridgwell, for instance, reported isolating *Salmonella *from approx. 4% of bird droppings in London [218]. Conversely, Cizec et al. reported isolating *Salmonella *from 19% of wild gulls sampled in the Czech Republic [219]. However, lower prevalence estimates among gulls have also been reported in the literature, in the range of 3-13%, and considerable differences between bird species and age groups seem to exist [219-221].

A number of human outbreaks have been linked to duckling exposure, and often both ducklings and chicken hatchlings are involved in the same outbreak, indicating great similarities in transmission and epidemiology (Additional file 2: Table S2). Analogous to chicken farms, it remains yet to be determined whether commercial duck and geese farms represent a substantial direct risk to human health. Similarly, the potential role of wild ducks and geese for human health is still subject to debate and conclusive evidence for or against an important role has yet to be presented (see [217] for a review of the subject). In conclusion, the risk associated with pet ducklings is high, but relatively well understood and bears great similarities to that observed for young chicken. Commercially reared or wild anseriform birds might conceivably pose a substantial risk to human health, but more data is needed before the subject can be evaluated conclusively.

### 4.4. *Salmonella *infections among columbiform birds

Doves and pigeons belong to the order of columbiform birds. Upon *Salmonella *infection, most adult birds show no or mild signs of disease, but severe paratyphoid disease with high mortality has been reported among young birds [222]. Clinical manifestations are variable and include gastro-enteritis, growth retardation, anorexia, depression, fever, torticollis, opisthotonos, oophoritis or orchitis, arthrosynovitis, and abscesses. While a variety of serotypes have been isolated from pigeons and doves, *Salmonella *Typhimurium var. Copenhagen phage types 2 and 99 are the most commonly isolated subtypes [223]. Intriguingly, the Typhimurium isolates from pigeons differ biochemically and antigenically from other Typhimurium isolates, likely indicating host adaptation of these Typhimurium subtypes to pigeons [217,222,223].

*Salmonella *appears to be a relatively common pathogen among pigeons and doves, but the prevalence seems to differ by serotype and habitat (see [217] for a review of the subject). Petersen [224], for instance, compared *Salmonella *prevalence in wild pigeons from urban areas and dairy farms in Colorado, and detected *Salmonella *in approx. 8% of samples from dairy-exposed pigeons but not in samples from pigeons in urban areas. However, the isolation of various *Salmonella *serotypes from wild pigeons in urban areas in Japan has also been reported, indicating a potential risk for human health [225]. Endemic infections among domestic pigeons in lofts have been described, and prevalence estimates of 25-30% within individual lofts have been reported [217]. *Salmonella *Typhimurium var. Copenhagen phage types 2 and 99 seem to be the predominant serotypes among domestic pigeons, but other serotypes have also been isolated [217].

In conclusion, *Salmonella *appears to be relatively common among pigeons. However, most infections seem to be due to host adapted subtypes of Typhimurium, and therefore likely only pose a limited risk to humans. In fact, to our knowledge, no zoonotic transmission from pigeons to humans has been documented in the literature, even though underreporting of human cases likely contributed to this lack of observation. *Salmonella *can also be found in wild doves, probably mostly at low prevalence. Broad-spectrum serotypes have been isolated from wild pigeons and environmental contamination appears to represent an important risk factor for shedding in doves and pigeons, indicating that such birds may represent potentially important vectors on livestock premises and possibly carry some direct risk for humans.

### 4.5. *Salmonella *infections among passerine birds, psittacine birds, and other non-domesticated birds

Passerine birds are commonly referred to as songbirds, while psittacine birds include parrots, cockatoos and parakeets. Both passerine and psittacine species are not only important wild bird species, but also represent popular pets and are often kept in zoological exhibits. Numerous *Salmonella *serotypes have been isolated from a variety of captive birds held as pets (Figure 1, Additional file 1: Table S1). Acute and chronic infections have been reported, which range from asymptomatic to clinically severe and can manifest as diarrhea, anorexia, dehydration, depression, crop stasis, septicemia or osteomyelitis [226-228]. For instance, *Salmonella *has been isolated from pet shop and household birds in Trinidad, imported finches, lories and parakeets in Japan, a variety of captive birds imported into Britain, numerous psittacine species held in Brazil, psittacine pet birds in Texas, Tennessee and Kansas, and captive as well as free-ranging parrots in Bolivia (Additional file 1: Table S1). *Salmonella *outbreaks with high bird mortalities have been described in zoologic exhibits, captive bird colonies and falcon collections [229-231].

Captive birds can also pose a *Salmonella *risk to humans, even though only a very limited number of cases have been documented (Additional file 2: Table S2). For instance, parakeets were involved in the transmission of *Salmonella *Typhimurium to a human infant and a cat, even though the exact transmission routes were not clearly determined [232].

Asymptomatic *Salmonella *carriage in wild birds is thought to be high, and wild birds have repeatedly been implicated as vehicles on farms and in feed mills [233-236]. Around the world, a variety of serotypes, including those frequently isolated from humans, have been isolated from free-ranging songbirds, parrots and parakees, with clinical manifestations ranging from asymptomatic to peracute death (Figure 1, Additional file 1: Table S1). Stress increases the risk of shedding, and in songbirds salmonellosis commonly peaks in winter months, likely due to crowding and increased contact rates at bird feeders [237]. *Salmonella *prevalence among birds at feeders is commonly higher than in the general population, and epidemics have been reported repeatedly, for example during the winter of 1997/98, when an epidemic affected songbirds across the eastern parts of North America [237-239].

Wild songbirds have also been repeatedly implicated as source of human infection (Additional file 2: Table S2). For instance, during the winter of 2000, Typhimurium isolates with identical Pulsed Field Gel Electrophoresis (PFGE) patterns were associated with a *Salmonella *outbreak among wild birds as well as human cases in New Zealand, and an epidemiologic link, potentially due to contaminated water, appears plausible [239,240]. Evidence also suggests that a large number of human Typhimurium cases in Norway were associated with wild bird contacts [241]. Other non-domesticated birds also potentially pose a risk to human health. In 2001 an elementary school Typhimurium outbreak involving at least 40 human cases was linked to dissecting owl pellets collected from captive owls, and more recently another outbreak in Massachusetts was also linked to owl pellets [242].

In conclusion, contacts with wild or captive birds pose a possible threat to human health, even though many epidemiological details remain to be understood. Birds, bird droppings, pellets, and feathers, as well as contaminated water and environments represent a potential risk.

## 5. Reptiles, Amphibians and Fish as Sources of Human Infection

Contact with reptiles, amphibians or pet fish also represents an important source of *Salmonella *infection (Additional file 2: Table S3). Reptiles, amphibians and fish have become popular pets in many countries. For example, approx. 3% of households in the USA own one or more reptiles as pets, resulting in a total of approx. 7.3 million reptiles [106]. The number of pet turtles has doubled in recent years, and in the USA approx. 2 million turtles are now kept in over 1 million households. More than 400 000 USA households keep snakes and in excess of 700 000 households own lizards [106]. Pet fish are present in an estimated 15 million USA households, with approx. 0.8 million households owning salt water fish tanks [191]. An estimated 1.3 million reptiles and 203 million fish were imported legally into the USA in 2005 alone, and a considerable number of pets are imported illegally each year [191]. Imports include captive-bred as well as wild-caught animals and the exotic pet trade poses a potential public health threat [191].

### 5.1. Reptiles and *Salmonella*

#### 5.1.1. *Salmonella *prevalence and serotype diversity among reptiles

*Salmonella *occurs naturally in the gastrointestinal tract of many reptiles, is commonly shed by these animals, and around the world a large number of serotypes have been isolated from feral and captive reptiles as well as their eggs (Figure 1, Additional file 1: Table S1). Clinical disease, including septicemia, osteomyelitis, salpingitis, nephritis, dermatitis and abscesses, seems to be occasionally associated with *Salmonella *infection in snakes, turtles, and lizards, but the overwhelming majority of infections in reptiles are undoubtedly asymptomatic; clinical salmonellosis in reptiles is rare, appears to be associated with underlying disease or other stressors, and a causal relationship between *Salmonella *infection and disease is generally difficult to establish conclusively [243-245]. Whether *Salmonella *infection causes diarrhea in reptiles is still subject to debate, and might depend on a variety of factors including host species and ambient temperature during infection [246,247]. For instance, links between a case of necrotizing gastritis in a snake and *Salmonella *infection or between atrophic gastritis in a tortoise and *Salmonella *has been suggested, but so far reports are predominantly anecdotal (see for instance [248] for a review).

Prevalence estimates in free-ranging reptiles vary widely, and a few studies report the absence of *Salmonella *in their study populations [249,250]. Prevalence estimates in other studies range from 6 to 100% in turtles, from 30 to 76% in lizards and from 54 to 100% in snakes [251-257]. It has been estimated that as many as 90% of all captive reptiles carry *Salmonella*, including a large number of 'reptile-associated' as well as "broad host-range" serotypes [196,246]. Some studies have investigated the efficacy of antimicrobial treatments, sometimes combined with physical treatments, in reducing reptile *Salmonella *carriage, and initial laboratory experiments proved promising [258-260]. However, the routine treatment of reptiles, eggs, or pond water on commercial farms is complicated by farming conditions, intermittent *Salmonella *shedding, transovarian infections, coprophagy, and environmental reservoirs, and these treatments appear to be associated with a heightened risk of antibiotic resistance [261-264]. *Salmonella *has been isolated from commercially raised turtles, crocodiles, alligators, and iguanas, their meat, or the farm environment, and contamination levels appear to be substantial, with prevalence estimates for farmed turtles as high as 40% (Additional file 1: Table S1). The stress of transportation and the close physical contact during transport may further contribute to *Salmonella *shedding, especially among baby turtles and lizards.

#### 5.1.2. The risk associated with contacts to reptiles

Human salmonellosis attributable to reptile exposure was first documented in the 1940s, and a large number of case reports have since described zoonotic transmissions of *Salmonella *from reptiles to humans (Additional file 2: Table S3). The exact number of reptile-associated salmonellosis cases among humans is difficult to determine, but one study estimated that in the USA, reptile exposure contributes approx. 70 000 human cases each year [265]. This represents 6% of all sporadic human cases, and reptile-associated cases are estimated to contribute 11% of sporadic human cases in the population < 21 years of age [265]. In the European Union, apparent prevalence estimates vary considerably among member states and over time [266]. In Sweden, for instance, 339 human reptile associated cases have been reported between 1990 and 2000, equaling approx. 5% of reported human cases [266,267]. This number increased markedly in subsequent years until a public education campaign was launched in 1997 [267].

A large number of human salmonellosis cases have been linked to contact with turtles, terrapins, snakes, and lizards such as iguanas, bearded dragons, geckos, and chameleons (Additional file 2: Table S3). Reptile-associated *Salmonella *infections in humans tend to be more likely associated with systemic disease than foodborne infections. Especially among children, the elderly or pregnant women, septicemia, meningitis, arthritis, soft-tissue abscesses, osteomyelitis, pericarditis, myocarditis, peritonitis and urinary tract infections have been repeatedly described, leading to severe disease and comparably high mortality rates (Additional file 2: Table S3). However, frequently only a single person or household is affect, and many reported cases involve children and infants. The reasons for the high prevalence among infants and children are not clear and might include biological, immunological and behavioral determinants [268,269]. Few reptile owners are aware of the disease risk. In the USA, the CDC recently reported that only approx. 20% of interviewed human cases were aware of the link between reptiles and *Salmonella *[269]. Good hand hygiene, which has been shown to be a very effective measure to prevent infection, may therefore not be strictly enforced [270].

*Salmonella *survives over long time periods in the environment, and a number of human outbreaks have been attributed to indirect reptile contact (Additional file 2: Table S3). Reptile-associated salmonellosis occurs frequently in small children, which are rarely allowed direct contact with snakes or lizards, strongly suggesting indirect exposure routes (Additional file 2: Table S3). In fact, a case-control study found presence of reptiles in the home to be a highly significant risk factor for salmonellosis in infants < 1 year of age, strongly suggesting a predominant role of indirect transmission [271,272]. In other cases, the evidence is even more compelling. For instance, in 1996 a human *Salmonella *outbreak with at least 65 cases was linked to contact with a wooded barrier around a Komodo dragon habitat in the Colorado zoo [270]. In 1994, hospitalized infants were infected with *Salmonella *Kintambo [271]. One infant's family owned a lizard which shed Kintambo and the infant's mother reported diarrheal illness shortly before giving birth, potentially indicating prior infection. In 2001, *Salmonella *Nima was isolated from a sick infant and a boa at the school where the infant's father worked [273]. The father reported carefully washing his hands after handling the snake or its container, but frequently draped the snake around his arm and did not change his clothes before handling the infant. In 2004, *Salmonella *Typhimurium was isolated from an 80 year old woman and the bowl in which her daughter's turtle was kept [274]. The women had no direct contact with the turtle or its bowl, but the bowl was cleaned in the kitchen sink. Given the large number of indirect transmissions, the USA CDC recommends that households with young children (i.e. < 5 years of age) do not own reptiles and that reptiles are not introduced into school settings. Several organizations have published information materials and recommendations concerning pet reptiles. In the USA, these include for instance governmental organizations such as the CDC and FDA, as well as professional organizations such as the American Veterinary Medical Association, the Association of Reptile and Amphibian Veterinarians, and the National Association of State Public Health Veterinarians (NASPHV). However, despite intense efforts awareness has remained limited.

Given the high prevalence of *Salmonella *among feral alligators, crocodiles, turtles, and lizards, contamination of surface water, ponds and other natural surfaces also represents a potential public health concern, but quantitative risk estimates are currently not available [275-279]. At least two human cases have been linked to reptile-contaminated surface water. In this instance, pet turtles were allowed to swim in a non-chlorinated in-ground pool frequented by the two human case patients [269].

In conclusion, direct or indirect exposures to reptiles clearly represent a substantial risk to human health. Infants and young children are at a particularly high risk, and severe clinical manifestations seem common. A considerable fraction of cases occur due to indirect contacts. Moreover, the prevalence of *Salmonella *shedding among captive reptiles is high, and clinical symptoms are rare. Large parts of the general population may therefore be affected. Legislative efforts have achieved substantial successes in reducing the risk of reptile-acquired infection. However, regulations have to be integrated with public education to achieve maximum compliance. Governmental agencies and several stakeholder groups work to increase consciousness. However, despite long-standing efforts, awareness of the *Salmonella *risk has remained comparably low. A number of alternative exposure routes can also lead to reptile-associated infections, and should be taken into consideration as appropriate.

#### 5.1.3. *Salmonella - *related regulations of pet turtle commerce

Up to the 1970s, pet turtles represented a major source of salmonellosis in the USA, annually contributing an estimated 14 to 23% of salmonellosis cases among children [280,281]. This prompted numerous state governments to mandate certification of *Salmonella *free status for all locally sold pet turtles, and since 1972 the USA FDA required similar certifications for all pet turtles sold in interstate commerce [281]. When these measures proved ineffective, the FDA posed a nation-wide ban on the sale and distribution of turtle eggs and small turtles with shells less than 4 inches (10.2 cm) in diameter in 1975 [281]. This legislation markedly decreased the number of reptile-associated cases, and has been estimated to prevent approx. 100 000 cases of salmonellosis, predominantly among young children, each year [281]. However, the ban did not prevent the sale of young turtles in all cases. Turtles less than 4 inches were still allowed to be sold for scientific, educational and exhibitional purposes, for export, or for purposes other than business, and the ban did not include marine turtles. In addition, small turtles were still illegally sold in pet stores and at unregulated vendors such as flea markets, and small turtles with shell diameters below 4 inches were still implicated in a considerable number of reptile-associated salmonellosis cases after the sale ban was enacted in 1975 (see for example [269,282]). In 2007, Louisiana Congressmen proposed the "Domestic Pet Turtle Market Access Act of 2007" to the US Senate, which aimed to overrule the sales ban if the seller met certain regulations regarding licensing, sanitization and consumer information. The specified reasons included the fact that the sale of lizards, snakes, frogs and other amphibians and reptiles as pets is not regulated by the FDA, even though they are known to carry *Salmonella*, and that *Salmonella *treatment technologies have advanced since the ban was initiated in 1975. The bill was referred to the Senate committee on agriculture, nutrition and forestry, but had no action taken and never passed.

### 5.2. Amphibians and *Salmonella*

The prevalence of *Salmonella *among amphibians seems to vary considerably by host species, even though the number of studies that analyze *Salmonella *shedding by amphibians is quite limited. *Salmonella *has frequently been isolated from frogs and toads, in which *Salmonella *infection seems to be predominantly asymptomatic (see for instance [253,283,284]). The prevalence of *Salmonella *among salamanders and newts appears to be lower than among frogs and toads, but the available data is currently relatively limited [283].

Contacts with toads or frogs clearly pose a potential risk to humans. In 2009, for instance, an outbreak of *Salmonella *Typhimurium, which affected people in more than 30 USA states, was associated with African dwarf frogs (Additional file 2: Table S3) [285]. Implicated frogs were traced back to a breeder in California, and Typhimurium isolates with PFGE patterns matching the outbreak strain were isolated from several environmental samples taken in the implicated breeding facility. In 2001 an outbreak of *Salmonella *Javiana in Mississippi was epidemiologically linked to contact with frogs and toads, even though the *Salmonella *serotype was not isolated from amphibians sampled during the outbreak investigation [286]. In 2000, frogs were epidemiologically implicated as the source of water contamination at a construction site in Australia, but no microbiological investigations of the frogs were performed [287]. Moreover, a case-control study estimated that the odds of *Salmonella *serogroup B or D infection among people < 21 years of age were 2.9 (95% Confidence Interval: 1.5-5.8) times higher if amphibians were present in the household, again indicating a potential risk posed by these animal exposures [265].

### 5.3. Fish and *Salmonella*

Some studies have investigated the prevalence of *Salmonella *among wild, pet or farmed fish in different ecological niches, sometimes with somewhat conflicting results. Gaetner et al. [288], for instance, reported isolating *Salmonella *from 17-33% of fish sampled in the San Marcos river in Texas and Miruka et al. [289] isolated *Salmonella *from approx. 31% of fish samples collected in Lake Victoria, Kenya. Broughton and Walker [290], however, estimated the *Salmonella *prevalence among farmed fresh-water fish in China at approx. 5%, and estimates for fish in retail markets in India have ranged from 10-28% [291].

Clinical disease associated with *Salmonella *infected fish have been described, mainly manifested as septicemia [292]. Yet, fish can also shed *Salmonella *in the absence of clinical signs, and after experimental inoculation of silver carp, shedding has been observed for periods of up to 14 days [293]. Fish feed appears to represent a considerable source of *Salmonella *infection in commercial aquacultures, and frequent bacterial carriage in raw materials paired with insufficient heat treatment appears to be the major driver [294].

#### 5.3.1. The risk associated with contacts to fish

A variety of *Salmonella *serotypes have been isolated from free-ranging or captive fish (Additional file 1: Table S1), and *Salmonella *is common in wild and, probably to a lesser extent farmed, fish. Occupational infections have been linked to contacts with contaminated fish, and ornamental fish tanks have also been linked to human *Salmonella *outbreaks (Additional file 2: Table S3). An Australian study, for instance, reported that 82% of human cases during a *Salmonella *Java outbreak had been directly or indirectly exposed to exotic fish tanks, and the outbreak strain was detected in implicated tanks [295]. Surprisingly, clinical disease in fish was observed in a large number of tanks, indicating a high pathogenic potential in the fish [295]. In conclusion, exposure to pet, wild or farmed fish represents a potential risk to humans, but the data available regarding infections acquired through contact with fish is currently scarce.

## 6. Invertebrates and *Salmonella*

*Salmonella *has been isolated from a large number of insects and worms including ants, flies, cockroaches, mealworms and mosquitoes (Additional file 1: Table S1). However, almost nothing is known about the pathogenic potential of *Salmonella *in invertebrates. *Salmonella*-induced mortality in butterflies and *Caenorhabditis elegans *has been described, and it appears likely that *Salmonella *can induce disease in other invertebrates [296,297]. Whether *Salmonella *can survive metamorphosis is equally unclear and results might be partially serotype, environment, and host dependent (see [298] for a review of the topic). In general, *Salmonella *intestinal carriage decreases sharply during pupation, likely due to major changes in the gut environment, and competition with other gut microorganisms such as *Proteus *has been shown to effectively suppress *Salmonella *growth in flies [298]. The public health relevance of insects might therefore depend on the life stage, the breeding environment and the insect species, and some insects apparently represent more competent hosts than others [298]. Insects and worms have been proposed as disease vectors for *Salmonella *on farms, agricultural fields and in households; invertebrates have been associated with *Salmonella *transmissions between animal feeds; biting mites have been shown to efficiently transmit *Salmonella *to chickens, and house flies have been implicated as Typhoid fever vectors in military camps (Additional file 1: Table S1). Moreover, insects can represent reservoir hosts, and therefore may play pivotal roles in *Salmonella *persistence.

## 7. Animal Contact in Public Settings as Source of Human Infection

### 7.1. Human outbreaks linked to animal contacts in public settings

*Salmonella *has repeatedly been isolated from captive and domestic animals held in public settings. For instance, a Korean study sampling clinically health zoo animals reported isolating *Salmonella *from approx. 6% of animals, including 30% of reptiles, 7% of birds and 1% of mammals [299]. Other studies report the isolation of *Salmonella *Dublin from a clinically healthy tiger at a zoo in the UK, from large cats and rodents in a zoo in India, from rhinoceroses in an animal park in the USA, from a newborn moose and an iguana in a zoo in Canada, from approx. 6.5% of animals in a zoo in Trinidad and from two animals in Swiss petting zoos (Additional file 1: Table S1). *Salmonella *has also been isolated from environmental surfaces in zoological gardens [300]. The health threat is aggravated by risky behaviors. For instance, one recent study found that 87% of visitors in Tennessee petting zoos had contact with potentially contaminated surfaces, and 74% had direct animal contact, but only 38% used hand sanitizer, while 49% had hand-to-face contacts with 22% eating or drinking in animal contact areas [301].

Animal contact in public settings therefore represents another source of human infection, and direct or indirect animal contacts in petting zoos, zoological parks, country fairs, or other settings represent threats to public health [190]. As mentioned above, in 1996, for instance, 65 children became sick after visiting the Denver zoo, due to indirect contact with a Komodo dragon [270]. In 1991, a visit to a science center in Washington was associated with 5 human salmonellosis cases, in 2000, at least 18 people were affected by a *Salmonella *outbreak linked to a petting zoo in Ohio, in 2001, at least 19 salmonellosis cases were linked to animal contact at an agricultural exhibit in Alberta, Canada, in 2003, at least 17 human *Salmonella *cases were linked to contact with a wallaby in a public setting in Michigan, and in 2005, 19 human cases were linked to direct or indirect contact with pigs in a public setting in Wisconsin [34,302,303].

In conclusion, animal contacts in public settings represent a *Salmonella *risk to occupationally exposed subpopulations as well as the general public, but management practices can effectively reduce the risk. To minimize risks, several governmental agencies have passed legislations governing animal exhibitions, and governmental as well as professional organizations have published related recommendations.

### 7.2. Current legislation and recommendations: case study USA

Minimizing the risk of disease transmission in public settings is a major concern for governments and professional organizations around the world. As one example, recommendations and legislation in the USA will be described in the following passage. The USA National Association of State Public Health Veterinarians (NASPHV), in conjunction with the CDC, published recommendations to prevent disease outbreaks in public settings, which are regularly updated. The recommendations are addressed to governmental agencies, educational settings, exhibit managers, veterinarians, caregivers, and visitors at particular risk of infection. The main focuses are on disseminating information, enhancing oversight and outbreak investigations, encouraging good hygiene practices, improving facility design, implementing disease monitoring and prevention systems, and prohibiting high risk contacts. To further address the threat, the CDC, the Association for Professionals in Infection Control and Epidemiology, the Animal-assisted Interventions Working Group and the NASPHV published a number of additional recommendations and the Association for Zoos and Aquariums has developed a certification program. Depending on the animal species, animal exhibits may also be subject to USDA inspections under the Animal Welfare act, but human disease risks are not explicitly addressed in these inspections. In addition, some states have passed additional legislations. For instance, North Carolina requires all animal exhibits with public access to obtain a license, Pennsylvania mandates minimum standards for exhibit sanitation, and Virginia requires a permit for the exhibition of wild animals. The relative effectiveness of these diverse mitigation strategies largely remains to be determined.

## 8. Conclusions

In conclusion, contact with animals is responsible for a number of human salmonellosis cases each year. A number of transmissions occur in the home, but others are occupational or related to public exposures in zoological gardens, schools, or state fairs. Infected animals can present with a great variety of clinical symptoms, and risk factors for transmission to humans clearly differ by animal species, age groups, animal purpose, and geographic region. However, some commonalities are clearly evident. Stress, concomitant disease, and contaminated feed represent universal risk factors for animal infection. Conversely, public awareness and proper hygiene practices are efficient measures to mitigate risks. In fact, frequent hand washing alone could likely prevent a substantial number of human infections each year. However, awareness of the risks is low and the collaboration between governmental agencies, professional organizations and special interest groups is necessary to resolve the problem. In some instances, new legislations or public awareness campaigns have led to dramatic decreases in *Salmonella *incidence. Yet, much remains to be done to safeguard public health and many aspects of *Salmonella *epidemiology remain to be discovered. *Salmonella *serotypes differ in host range and distribution among host species, but our understanding of the molecular and evolutionary determinants of these host range differences is still limited and the public health implications are currently difficult to assess. In summary, the risks associated with animal contacts are diverse and much remains to be uncovered, but we already posses important clues to manage the risks.

## Competing interests

The authors declare that they have no competing interests.

## Authors' contributions

KH, MW and AIMS conceived and outlined the study. KH and AIMS reviewed the pertinent scientific literature and collated references and tables. KH drafted the manuscript. All authors have read and approved the final manuscript.

## Supplementary Material

Additional file 1 **Table S1**. Overview of *Salmonella *serotypes isolated from animals in different geographic regions. Click here for file

Additional file 2 **Table S2**. Documented reports of Salmonella transmission from birds to humans available in the peer-reviewed literature or otherwise published by public health agencies. **Table S3**. Documented reports of *Salmonella *transmission from reptiles, amphibians fish to humans available in the peer-reviewed literature or otherwise published by public health agencies. Click here for file
