# Supplementary material for: Animal contact as a source of human non-typhoidal salmonellosis
Source: Vet Res. 2011 Feb 14;42(1):34. doi: 10.1186/1297-9716-42-34 (PMC3052180; doi:10.1186/1297-9716-42-34)
Supplement: Additional file 1 — Table S1. Overview of Salmonella serotypes isolated from animals in different geographic regions. [file 1297-9716-42-34-S1.RTF]

 Table S1: Overview of Salmonella serotypes isolated from animals in different geographic regions.
Host Species	Salmonella serotype	Country/ Geographic Region1	Reference	
				
Livestock				
Cattle	3,15:e	NAM	[30]	
	4,5,12:b:-	SEA	[217]	
	4,5,12:i:-	NAM	[3]	
	6,7:-:1,5	NAM	[30]	
	Aberdeen	AU	[75]	
	Abortusbovis	EU	[42]	
	Adelaide	AU, SEA	[114, 119, 215]	
	Agama	AF, EU	[46, 59]	
	Agona	AU, EU, NAM,SEA	[3, 21, 26, 27, 30, 54, 59, 64, 111, 119, 130, 142, 151, 161, 215]	
	Ajiobo	EU	[59]	
	Alagbon	AF	[46, 131]	
	Albany	NAM	[111]	
	Anatum	AF, AU, EU, NAM, SEA
	[3, 4, 16, 21, 27, 30, 42, 49, 53, 54, 59, 74, 75, 111, 114, 119, 130, 145, 215, 217]	
	Arizona	NAM, SEA	[112, 119, 161, 162]	
 	Baiboukum	AF	[216]	
 	Bardo	NAM	[161]	
	Barranquilla	NAM	[27, 111]	
	Bareilly	NAM, SA	[3, 152]	
	Bergen	NAM	[27]	
	Berta	EU	[59]	
	Binza	EU	[59]	
	Blockley	SEA	[119]	
	Bovismorbificans	AF, AU, EU, SEA	[42, 114, 119, 145, 215]	
 	Bradford	AF	[145]	
 	Braenderup	NAM	[54, 130, 161]	
 	Brancaster	AF	[145]	
 	Brandenburg	AU, NAM	[43, 161]	
	Bredeney	EU, NAM	[48, 53]	
	Brunei	SEA	[165]	
	Bury	AF	[46]	
	Carrau	NAM	[54]	
	Cerro	NAM	[16, 21, 27, 49, 53, 54, 130, 214]	
	Chandans	AF	[46]	
	Charity	AU	[215]	
	Chester	AU	[114, 215]	
 	Chile	AF	[216]	
	Chingola	SEA	[119]	
	Choleraesuis	NAM	[30]	
	Coquilhatville	AF	[46]	
	Cubana	NAM, SEA	[27, 30, 54, 111, 119]	
	Derby	AF, EU, NAM, SEA	[3, 59, 112, 114, 145, 161, 217]	
	Denver	NAM	[28]	
	Drypool	NAM	[54]	
	Dublin	AF, AU, EA, EU, NAM, SEA	[1, 5, 11, 21, 42, 46, 54, 59, 69, 91, 111, 112, 118, 119, 142, 145, 151, 161, 162, 189, 212, 215, 216]	
	Durbanville	AF	[145]	
	Elizabethville	AF	[46]	
 	Emek	SEA, WA	[119, 154]	
	Entebbe	AF	[131]	
	Enteritidis	AF, EA, EU, SA, SEA	[5, 59, 69, 138, 152, 161]	
 	Essen	AF	[145]	
	Fresno	NAM	[130]	
	Give	AU, NAM	[3, 21, 27, 30, 54, 75, 111, 130, 162, 186]	
	Goldcoast	EU	[59]	
	Guildford	AF	[5]	
	Hadar	SEA	[119]	
	Hartford	NAM	[3, 27]	
	Havana	AU, NAM, SEA	[30, 54, 111, 114, 119, 130, 215]	
	Heidelberg	AF, NAM, SEA	[8, 91, 112, 119, 130, 145]	
 	Hvittingsfoss	AF, SEA	[119, 145]	
	Indiana	EU	[59]	
	Infantis	AU, EA, EU, NAM, SEA	[3, 21, 26, 27, 54, 59, 69, 91, 114, 119, 130, 162, 215]	
	Jacksonville	AF	[46]	
	Java	NAM, SEA, WA	[119, 154, 161]	
	Javiana	SEA	[119]	
 	Jedburgh	AF	[145, 216]	
	Johannesburg	AF	[46]	
	Kampala	AF	[131]	
	Karamoja	AF	[131]	
	Kentucky	NAM, SEA	[3, 4, 16, 21, 27, 30, 54, 74, 111, 119, 130, 214]	
	Kedougou	EU	[59]	
 	Kiambu	NAM	[27, 130, 161]	
	Kingshasa	NAM	[26]	
	Kikoma	AF	[131]	
	Klapmuts	AF	[131]	
	Kokomlemle	AF	[131]	
	Kottbus	NAM	[112]	
	Krefeld	NAM	[112]	
	Kumasi	SEA	[119]	
	Kuru	AF	[131]	
	Lexington	SEA	[217]	
	Lille	AF, NAM	[4, 21, 26, 30, 216]	
	Litchfield	NAM, SEA	[30, 119]	
	Livingston	NAM, SEA	[21, 27, 119]	
	Llandoff	EU	[131]	
	London	SEA	[119, 217]	
	Louwbester	AF	[131]	
	Manila	NAM	[54]	
	Mathura	SA	[131]	
	Mbandaka	EA, NAM, SEA	[4, 11, 16, 21, 27, 30, 53, 54, 86, 111, 119, 130, 161, 162]	
	Meleagridis	NAM	[4, 21, 27, 30, 54, 111, 130]	
	Minnesota	NAM	[21, 130]	
	Mishmarhaemek	AF	[5]	
	Monschaui	AF	[46]	
	Montevideo	EA, EU, NAM	[3, 4, 16, 21, 26, 27, 30, 54, 69, 74, 86, 91, 130]	
	Moroto	AF	[131]	
	Muenchen	AF, AU, NA	[3, 54, 75, 114, 130, 161, 216]	
	Muenster	AF, NAM	[3, 21, 27, 30, 54, 74, 86, 91, 130, 161, 162, 177]	
	Newbrunswick	NAM	[49]	
	Newington	AF, NAM	[21, 26, 27, 54, 74, 145]	
	Newport	AF, AU, EU, NAM, SEA	[3, 21, 26, 27, 42, 44, 54, 59, 111, 112, 114, 119, 130, 145, 161, 162, 217]	
	Nigeria	AF	[46]	
	Nienstedten	NAM	[91]	
	Ohio	NAM	[27]	
	Ona	AF	[46, 131]	
	Oranienburg	AF, AU, NAM	[3, 27, 46, 53, 114, 214]	
	Orientalis	SEA	[119]	
	Orion	AU, NAM	[4, 75, 215]	
	Oslo	SEA	[119]	
	Othmarschen	NAM	[54]	
	Paratyphi B	AF, EU, NAM, SEA	[46, 71, 99, 119, 130]	
	Panama	NAM	[130]	
 	Parow	AF	[145]	
	Raus	SEA	[119]	
	Reading	EU, NAM	[4, 21, 27, 49, 54, 130, 161]	
	Rissen	SEA, NAM	[161, 165, 217]	
 	Rubislaw	AF, AU, SEA	[46, 215, 217]	
	Saintpaul	AF, AU, NAM, SEA, WA	[112, 114, 119, 145, 154]	
	San Diego	NAM	[27]	
	Schwarzengrund	AF, EU, NAM, SEA	[21, 54, 59, 119, 216]	
	Senftenberg	AU, EU, NAM, SEA	[3, 4, 16, 27, 54, 75, 111, 119, 130, 215]	
	Stanleyville	AF	[46]	
 	Sofia	SEA	[119]	
 	Stanley	SEA	[119, 165]	
	Sternschanze	AF	[216]	
	Stourbridge	EU	[59]	
	Subsp. II	AF	[216]	
	Taksony	NAM	[54]	
 	Tamale	AF	[145]	
	Tennessee	NAM	[3, 27, 54, 86, 111, 112]	
 	Thompson	EU, NAM	[3, 27, 30, 54, 59, 112, 161, 205]	
 	Tinda	AF	[145]	
	Typhimurium	AF, AU, EA, EU,NAM, SA, SEA, WA	[3-5, 11, 21, 27, 30, 42, 46, 49, 54, 59, 69, 70, 75, 86, 90, 91, 112, 114, 118, 119, 142, 145, 151, 152, 154, 161, 162, 212, 214-217]	
 	Uganda	NAM	[3, 27, 161]	
	Urbana	SEA	[119]	
	Virchow	SEA	[119]	
 	Vleuten	WA	[154]	
	Wangata	AF	[46]	
	Waycross	AF	[145]	
	Weltevreden	SEA	[119, 165, 217]	
	Worthington	NAM	[162]	
				
pigs	04,12:d-	EA, SEA	[125, 217]	
	4,12:i:-	NAM	[73]	
	4,12:-:-	NAM	[73]	
	4,5,12:i:-	NAM	[73]	
	4,5,12:b:-	SEA	[217]	
	4,5,12:-:-	NAM	[133]	
	O:13,22,23 Hi	EU	[19]	
	O:i:1,2	NAM	[133]	
	6,7,14:-	NAM	[73]	
	6,7:-:-	NAM	[133]	
	1:9,12:-	AF	[147]	
	28:y:-	NAM	[73]	
	Aberdeen	SEA	[160]	
	Adelaide	AU	[114]	
	Agama	AF	[46]	
	Agona	AU, CAM, EA, SEA, NAM	[11, 19, 55, 64, 73, 85, 91, 112, 119, 125, 161, 185]	
	Ajiobo	CAM	[185]	
	Alachua	EA, NAM	[84, 125]	
	Anatum	AF, AU, EA, EU, NAM, SEA	[11, 19, 55, 84, 85, 91, 112, 114, 125, 133, 147, 161, 165, 217]	
	Arizonae	NAM, SEA	[112, 119]	
	Arkansas	NAM	[55]	
	Bareilley	SA	[152]	
	Binza	NAM	[55]	
	Blockley	AF, SEA, 	[119, 147]	
	Bovismorbificans	EU, AU	[42, 114]	
	Brandenburg	EA, EU, NAM	[11, 19, 58, 73, 85, 125, 143, 212]	
	Bredeney	EU, NAM	[19, 48, 112]	
	Brunei	SEA	[165]	
	Chester	AU	[114]	
	Choleraesuis	EA, EU, NAM, SEA	[42, 58, 69, 112, 119, 143]	
	Cerro	EA	[85]	
	Corvallis	EA	[125]	
	Derby	AU, EU, EA, NAM, SEA
	[11, 19, 55, 58, 69, 73, 79, 84, 85, 91, 106, 112, 114, 119, 125, 133, 143, 159-162, 165, 212, 217]	
	Dublin	EU	[42]	
	Emek	SEA	[165]	
	Enteritidis	AF, AU, EA, EU, NAM, SA	[42, 91, 112, 114, 125, 138, 147, 152]	
	Gaminara	AF	[131, 147]	
	Garba	AF	[131]	
	Give	EA, EU, NAM	[42, 112, 125]	
	Goldcoast	EU	[58, 159]	
	Gokul	AF	[131]	
	Hadar	NAM,AF	[91, 147]	
	Haifa	EA	[85]	
	Havana	AF, AU, CAM, EA, NAM	[73, 114, 125, 147, 185]	
	Heidelberg	EU, NAM	[8, 19, 55, 84, 91, 112, 133, 185]	
	Heilbron	SEA	[160]	
	Houten	SEA	[119]	
	Infantis 	AU, EA, EU, NAM	[11, 19, 55, 73, 85, 91, 106, 112, 114, 125, 133, 159, 207, 212]	
	Javiana	EA	[160]	
	Johannesburg	NAM	[55]	
	Kedougou	EU	[19, 58]	
	Kentucky	AF,NAM	[55, 84, 147]	
	Kiambu	AF	[147]	
	Krefeld	SEA	[119]	
	Leoben	AF	[147]	
	Livingstone	AF, EA, EU, NAM	[46, 125, 147, 159, 161, 212]	
	Litchfield	NAM	[55]	
	Lomita	SEA	[119]	
	London	EA, EU, NAM, SEA	[73, 85, 125, 160, 212, 217]	
	Mbandaka	EU, NAM	[19, 55, 83, 91]	
	Meleagridis	CAM, EA	[85, 125, 185]	
	Minesota	EA	[11]	
	Montevideo	EA, EU, NAM	[19, 55, 69, 112, 143]	
	Mowanjum	EA	[85]	
	Muenchen	AU	[114]	
	Muenster	EA, NAM	[55, 85]	
	Napoli	EU	[18]	
	New Brunswick	NAM	[55]	
	Newington	EU	[42]	
	Newport	AU, EA, EU, SEA	[19, 42, 114, 125, 165, 217]	
	Nigeria	EA	[85]	
	Nienstedten	NAM	[112]	
	Norwich	EU	[131]	
	Ohio	NAM	[73, 133, 161]	
	Oranienburg	AU	[114]	
	Pakistan	EA	[11]	
	Panama	EA, EU, SEA	[19, 58, 125, 165, 212, 217]	
	Paratyphi B	EU, SA,SEA	[42, 119, 152]	
	Poona	AF	[46]	
	Putten	NAM	[73]	
	Rissen	SA, SEA	[165, 217]	
	Romanby	CAM	[185]	
	Salinatis	EA	[85]	
	Saintpaul	AU	[114]	
	Schwarzengrund	NAM	[55, 133]	
	Senftenberg	AU, EA, EU, NAM, SEA	[19, 73, 85, 114, 161, 165]	
	Stanley	EA, SEA	[125, 160, 165]	
	Stanleyville 	EA	[85]	
	Taksony	EA	[125]	
	Thompson	EU, NAM	[91, 143]	
	Tennessee	AU, SEA	[114, 160]	
	Tshiongwe	EA	[85]	
	Typhimurium	AU, CAM, EA, EU, NAM, SA, SEA	[11, 19, 42, 55, 58, 69, 70, 73, 85, 91, 106, 112, 114, 119, 125, 133, 143, 152, 159-162, 165, 185, 207, 212, 217]	
	Typhisuis	NAM	[112]	
	Uganda	EA	[125]	
	Umbilo	EU	[143]	
	Vrindaban	SA	[131]	
	Virchow	EA, SEA	[125, 160]	
	Weltevreden	EA, SA, SEA	[85, 119, 160, 165, 217]	
	Wien	EU	[19]	
	Worthington	EU, NAM	[55, 58, 73, 207]	
	Zanzibar	EA	[11]	
				
  sheep & goats	4,12,i:-B	NAM	[28]	
 	Abortusovis	EU	[93]	
	Adelaide	AU	[65, 114]	
 	Agona	AU, NAM	[65, 112]	
 	Anatum	AU, NAM, WA	[28, 65, 114, 120, 154]	
 	Arizonae	NAM	[112, 136, 161]	
 	Bovismorbificans	AU, EU,WA	[42, 65, 114, 120, 154, 215]	
	Braederup	WA	[154]	
 	Brandenburg	AU, WA	[43, 154]	
	Butantan	WA	[154]	
 	Cerro	WA	[154]	
 	Chester	AU, WA	[65, 114, 154]	
 	Concord	WA	[154]	
 	Derby	AU	[114, 120, 215]	
 	Diarizonae	EU	[6, 188]	
 	Dublin	AU, EU, NAM, SEA	[42, 112, 119, 215]	
 	Eastbourne	WA	[154]	
 	Enteritidis	AU	[120]	
	Friedrichsfelde	NAM	[112]	
	Give	AF	[148]	
 	Guilford	WA	[154]	
 	Haifa	WA	[154]	
 	Havana	AU, WA	[114, 154]	
 	Heidelberg	AF, SEA	[119, 148] 	
 	Indiana	EU	[139]	
 	Infantis	AU	[114, 215]	
	Javiana	SEA	[119]	
	Kisii	SEA	[119]	
 	Kottbus	WA	[154]	
	Krefeld	NAM	[112]	
	Litchfield	SEA	[119]	
 	Montevideo	EU, NAM	[105, 112, 195]	
 	Muenchen	AU, NAM	[28, 65, 114]	
 	Newington	AU	[120]	
 	Newport	AU, SEA, WA	[114, 119, 154]	
 	Oranienburg	AU	[187]	
	Paratyphi B	SEA,WA	[119, 154]	
	Poona	AF, WA	[148, 154]	
 	Reading	AF, WA	[148, 154]	
 	Saintpaul	AU	[65, 120]	
 	Sofia	SEA, WA	[119, 154]	
	Subsp. II	AU	[215]	
	Tennessee	AU	[114]	
 	Thompson	EU, NAM	[105, 112]	
	Typhimurium	AF, AU, EU, NAM, SEA, WA	[24, 60, 65, 90, 112, 114, 119, 120, 148, 154, 161, 215]	
 	Uganda	WA	[154]	
 	Urban	NAM	[28]	
 	Urbana	NAM	[28]	
	Virchow	SEA	[119]	
	Weltevreden	SEA	[119]	
				
Companion animals			
  horses	4,12,24:i:-	NAM	[200]	
	9,12:-:-	EU	[225]	
	Abortus equi	EU, NAM	[42, 150, 176]	
 	Aertrycke	NAM	[67]	
	Agama	EU	[225]	
 	Agona	AU, EU, NAM	[7, 17, 62, 104, 108, 112, 166, 200, 225]	
 	Anatum	AU, EU, NAM, SEA	[7, 17, 68, 104, 108, 112, 119, 150, 184, 200, 225]	
 	Arizona	EU, NAM	[112, 210]	
 	Bardo	NAM	[161]	
 	Bareilly	EU,NAM	[150, 210]	
	Barranquilla	EU	[225]	
	Birkenhead	AU	[184]	
 	Blockley	EU, NAM	[161, 210]	
	Bornum	AU, NAM	[112, 184]	
 	Bovismorbificans	AU, EU, SEA	[17, 119, 184, 211, 225]	
 	Braenderup	EU,NAM	[161, 225]	
	Brandenburg	AU	[43]	
	Bredeney	AU	[184]	
 	Cerro	NAM	[112]	
	Charity	AU	[17]	
	Chester	AU	[17, 184]	
	Choleraesuis	EU	[225]	
	Cubana	NAM	[112]	
 	Derby	AU, EU,NAM	[7, 17, 225]	
 	Drypool	EA, NAM	[200]	
 	Dublin	EU	[42, 176, 225]	
	Eastbourne	AU	[17]	
 	Enteritidis	EU, NAM	[108, 150, 210, 211, 225]	
	Give	AU, EU, NAM	[112, 184, 225]	
	Goldcoast	EU	[211]	
 	Good	NAM	[7, 150]	
	Haessarek	AU	[17]	
 	Hadar	EU	[210, 211, 225]	
	Havana	AU	[17]	
	Heidelberg	EU, NAM	[108, 112, 150, 161, 225]	
	Indiana	EU	[225]	
 	Infantis	AU,NAM	[112, 184, 200, 211]	
	Inganda	SEA	[119]	
	Johannesburg	NAM	[112]	
	Kentucky	NAM	[112]	
	Kingston	EU	[225]	
	Kottbus	AU, EU, NAM	[17, 112, 225]	
 	Krefeld	NAM	[107, 112]	
	Leatherhead	EU	[225]	
	Lexington	EU	[225]	
	Livingston	EU	[211, 225]	
	London	NAM	[150, 166]	
 	Manhattan	EU, NAM	[63, 211, 225]	
 	Meleagridis	AU, NAM	[17, 200]	
 	Montevideo	EU, NAM	[7, 200, 225]	
	Muenchen	AU, EU	[184, 225]	
 	Muenster	EU, NAM	[108, 225]	
	Nejle	EU	[225]	
	New Brunswick	AU	[184]	
 	Newington	AU, NAM	[104, 108, 150, 184]	
 	Newport	AU, EU, NAM	[7, 17, 68, 112, 150, 161, 184, 225]	
 	Ohio	AU	[184]	
	Ohlstedt	AU	[184]	
	Onderstepoort	AU	[184]	
	Oranienburg	EU, NAM	[108, 112, 150, 225]	
 	Oregon	EU	[42]	
 	Panama	EU, NAM	[108, 225]	
 	Paratyphi B	NAM	[7, 68]	
	Poona	NAM	[108]	
	Potsdam	AU	[184]	
	Reading	EU	[225]	
	Rubislaw	NAM	[112]	
 	Saintpaul	AU, EU, NAM	[17, 68, 112, 150, 184, 225]	
	San Diego	NAM	[112]	
 	Senftenberg	NAM	[166]	
 	Siegburg	NAM	[7]	
	Singapore	AU	[17]	
	Stanley	EU	[225]	
	Stanleyville	EU	[225]	
	Taksony	NAM	[112]	
 	Tennessee	EU, NAM
	[108, 112, 184]	
	Thompson	AU, EU, NAM	[112, 184, 211]	
 	Typhimurium	AU, EU, NAM	[17, 24, 42, 63, 90, 107, 108, 112, 141, 150, 161, 166, 176, 184, 200, 210, 211, 219, 225]	
 	Virchow	AU, EU	[17, 184, 210, 225]	
	Wandsworth	AU	[184]	
	Warragui	AU	[17]	
	Waycross	AU	[184]	
	Worthington	AU, NAM	[112, 184]	
				
  dogs & cats	4:i:-	EU	[213]	
	Adelaide	NAM	[112]	
 	Agona	NAM	[31, 112, 132, 161]	
 	Anatum	NAM	[32, 112]	
	Arizonae	NAM	[112]	
 	Bareilly	EU, NAM, SEA	[32, 42, 119]	
 	Bovismorbificans	EU	[42, 213]	
 	Braenderup	NAM	[117]	
 	Brandenburg	AU, NAM	[43, 132]	
 	Corvallis	WA	[127]	
 	Derby	NAM, SEA	[32, 119]	
 	Dublin	AF, EU	[42, 46]	
 	Enteritidis	EA, EU, NAM, SEA, WA	[13, 42, 82, 112, 119, 132, 197, 213]	
 	Give	NAM, SEA	[32, 119]	
 	Hadar	NAM	[31, 117, 132]	
 	Heidelberg	NAM	[31, 112, 132]	
 	Indiana	NAM	[132]	
 	Infantis	NAM	[31, 112, 132]	
 	Johannesburg	NAM	[31]	
	Jos	AF	[46, 131]	
 	Kentucky	NAM	[132]	
	Kraaifontein	AF	[131]	
	Krefeld	NAM	[112]	
 	Meleagridis	NAM	[31, 32]	
 	Minnesota	NAM	[32]	
 	Montevideo	NAM	[32, 132]	
	Muenchen	NAM	[112]	
 	Muenster	NAM	[31, 112, 177]	
	Newington	NAM	[32]	
 	Newport	EU, NAM	[31, 42, 112, 161]	
 	Oranienburg	NAM	[32]	
 	Ouakam	NAM	[132]	
 	Paratyphi B	EU, NAM,SEA	[32, 42, 119]	
 	Reading	NAM	[31]	
	Rubislaw	NAM	[112]	
 	Schwarzengrund	NAM	[117, 132]	
 	Senftenberg	EU	[42]	
	Stanley	SEA	[119]	
 	Tennessee	NAM	[32]	
 	Thompson	NAM	[112, 132]	
	Typhimurium	EA, EU, NAM, SA, WA	[13, 24, 31, 82, 90, 112, 132, 137, 161, 194, 213]	
 	Uganda	NAM	[31]	
	Worthington	NAM	[112]	
  feed/treats	4,12:-:-	NAM	[77]	
	Aberdeen	EA	[224]	
 	Agona	NAM	[76, 77, 220]	
	Albert	NAM	[77]	
 	Anatum	NAM	[220]	
 	Borreze	AU	[224]	
 	Bovismorbificans	NAM	[76]	
 	Brandenburg	AU, NAM	[77, 220, 224]	
	Bredeney	NAM	[220]	
 	Derby	NAM	[76, 220]	
 	Enteritidis	AU	[224]	
	Freetown	NAM	[220]	
	Gaminara	NAM	[220]	
 	Give	NAM	[76]	
	Grampian	NAM	[220]	
	Hadar	NAM	[77]	
 	Havana	AU, SEA	[224 ]	
	Heidelberg	NAM	[77, 220]	
 	II:ROUGH-O:-:-	NAM	[76]	
	I:ROUGH-O:r:1,2	NAM	[77]	
	I:ROUGH-O:z10:enx	NAM	[77]	
 	Infantis	EA, NAM	[77, 220, 224]	
	Jerusalem	NAM	[220]	
	Johannesburg	NAM	[220]	
 	Kentucky	AU, NAM	[77, 224]	
 	London	AU, NAM	[76, 224]	
 	Manhattan	NAM	[76]	
 	Mbandaka	AU, NAM	[77, 220, 224]	
	Meleagridis	NAM	[220]	
 	Montevideo	AU, NAM, SEA	[220, 224]	
	Muenchen	NAM	[220]	
 	Newport	NAM	[220]	
 	Ohio	AU, NAM	[220, 224]	
 	Orion	AU, NAM, SEA	[220, 224]	
	Schwarzengrund	NAM	[77]	
 	Senftenberg	NAM, SEA	[220, 224]	
	Thompon	NAM	[77]	
	Typhimurium	AU, EU, NAM	[76, 77, 220, 224]	
	Uganda	NAM	[220]	
 	Worthington	NAM	[76, 220]	
				
Rodents	Agama	AF	[46]	
 	Agona	NAM	[124]	
	Ajiobo	AF	[46]	
 	Amsterdam	NAM	[204]	
 	Anatum	NAM	[124]	
	Augustenborg	SEA	[119]	
	Bareilly	EU	[42]	
	Bovismorbificans	EU	[42]	
	Bukuru	AF	[46, 131]	
	California	NAM	[134]	
	Chester	AF	[46]	
	Dublin	EU	[42]	
 	Enteritidis	EU, NAM, SA	[35, 42, 57, 102, 134, 194, 203]	
	Gaminara	NAM	[134]	
	Garba	AF	[46]	
	Heidelberg	NAM, SEA	[119, 134]	
	Hessarek	AF	[46]	
	Kentucky	SEA	[119]	
	Mgulani	SEA	[119]	
	Okefoko	AF	[46, 131]	
 	Oranienburg	AF, NAM	[46, 124]	
	Oxford	EU	[131]	
	Paratyphi B	EU	[42]	
	Stanley	EU, SEA	[42, 119]	
	Teko	AF	[131]	
	Teshie	AF	[131]	
 	Typhimurium	EA, EU, NAM, SAM	[24, 42, 72, 102, 134, 226]	
	Virchow	AF	[46]	
	Weltevreden	SEA	[119]	
				
Non-traditonal pets and wildlife			
badgers	Agama	EU	[222]	
	Binza	EU	[222]	
	Enteritidis	EU	[144]	
	Give	EU	[144]	
	Lomita	EU	[222]	
	Newport	EU	[144]	
	Ried	EU	[222]	
	Umbilo	EU	[144]	
bisons	Typhimurium	NAM	[161]	
chipmunks	Typhimurium	NAM	[24]	
coyote	Typhimurium	NAM	[24]	
elephants	Enteritidis	AF	[223]	
elks	Muenchen	NAM	 [162]	
	Typhimurium	NAM	[24, 80]	
foxes	Bovismorbificans	EU	[42]	
	Dublin	EU	[42, 61]	
 	Enteritidis	EU	[144]	
 	Hessarek	EU	[98]	
 	Kottbus	EU	[98]	
	Subsp. IIIb	EU	[98]	
	Typhimurium	EU	[98]	
hedgehogs	Enteritidis	AU, EU	[20, 122, 157]	
	Sofia	EU	[131]	
	Tilene	NAM	[36]	
	Typhimurium	AU, EU	[97, 122, 201]	
hippopotami	Aberdeen	AF	[92, 223]	
	Bareilly	AF	[92]	
	Miami	AF	[92, 223]	
	Typhimurium	AF	[92, 223]	
	Uganda	AF	[92, 223]	
marine mammals	Adelaide	AU	[114]	
(e.g., sea lions,	Derby	AU	[114]	
seals, whales etc)	Enteritidis	AN	[167]	
	Havana	AN	[167]	
	Montevideo	NAM	[202]	
 	Muenchen	AU	[114]	
	Newport	AN, NAM	[167, 202]	
 	Panama	AU	[114]	
	Saintpaul	AU, NAM	[114, 202]	
 	Senftenberg	AU	[114]	
 	Tennessee	AU	[114]	
 	Typhimurium	AN, AU	[114, 167]	
mink	Dublin	EU	[61]	
mongoose 	Agona	SAM	[72]	
 	Corvallis	SAM	[72]	
	Johannesburg	SAM	[72]	
 	Panama	SAM	[72]	
	Wernigerode	SAM	[72]	
mole	Vredelust	AF	[131]	
non-human 	Hindmarsh	SEA	[119]	
primates	Lishabi	AF	[46, 131]	
	Paratyphi B	SEA	[119]	
opossums	Glostrup	SAM	[72]	
 	Manhattan	SAM	[72]	
 	Miami	SAM	[72]	
 	Montevideo	EU	[221]	
 	Oranienburg	NAM	[116]	
 	Parera	SAM	[72]	
raccoons	Bardo	NAM	[47]	
	Berta	NAM	[47]	
	Hartford	NAM	[47]	
	Infantis	NAM	[47]	
	Newport	NAM	[47]	
	Oranienburg	NAM	[47]	
	Paratyphi B	NAM	[47]	
	Thompson	NAM	[47]	
	Typhimurium	NAM	[24, 47]	
rhinoceros	Typhimurium	AF	[223]	
sea otter	Enteritidis	NAM	[202]	
squirrels	Kentucky	NAM	[116]	
	Oranienburg	NAM	[116]	
	Typhimurium	NAM	[24]	
various ungulates	Paratyphi B	SEA	[119]	
(e.g. mouse deer)	Typhimurium	SEA	[119]	
white-tailed deer	Bareilly	EU	[42]	
	Brandenburg	AU	[43]	
	Bredbey	EU	[48]	
	Enteritidis	NAM	[180]	
	Infantis	NAM	[180]	
	Litchfield	NAM	[180]	
	Oranienburg	NAM	[28]	
	Dessau	NAM	[180]	
wild boar	Arizonae	EU	[144]	
	Worthington	EU	[144]	
wild felids 	Blockley	SEA	[119]	
(e.g., leopards, cougars, tigers, lions etc.)	Bredney	NAM	[45]	
	Cerro	NAM	[45]	
	Dublin	NAM	[45]	
	Enteritidis 	NAM	[45]	
	Hadar	NAM	[45]	
	Johannesburg	NAM	[45]	
	Kentucky	NAM	[45]	
	Meleagridis	NAM	[45]	
	Muenster	NAM	[45]	
	Panama	NAM	[45]	
	Poona	NAM	[45]	
	Typhimurium	NAM, SA	[45, 162, 194]	
woodchucks	Oranienburg	NAM	[116]	
				
Birds				
chicken	4,5,12:b:-	EU	[34]	
	4,5,12:i:-	NAM	[37, 110]	
	4,12:b:-	EU	[34, 38]	
	6,7,:-:1,5	NAM	[94]	
	Agona	AF, EA, EU, NAM, SEA, WA	[11, 33, 40, 64, 69, 91, 119, 172, 174, 209, 212, 218]	
	Anatum	EA, EU, NAM, SEA	[42, 91, 119, 135, 160, 174]	
	Arizona	SAM	[196]	
	Alachua	NAM, WA	[154, 181]	
	Alamo	AF	[209]	
 	Albany	CAM, EA, WA	[11, 154]	
	Bareilly	EA, EU, NAM, SEA	[11, 42, 94, 119]	
	Berta	NAM	[40, 174]	
	Binza	EU	[218]	
 	Blockley	EA, EU, NAM, SEA	[11, 34, 119, 123, 135, 162, 165]	
	Bovismorbificans	EU, SEA	[119, 172]	
	Braenderup	EU, SEA	[119, 218]	
	Bredney	EA	[135]	
	Chester	SEA	[119]	
	Coleypark	AF	[131]	
 	Concord	WA	[154]	
	Corvallis	EA, EU	[11, 218]	
	Cubana	EU	[218]	
	Derby	NAM, SEA	[94, 119]	
	Djugu	EU	[172]	
	Dublin	AF	[209]	
	Duivenhoks	AF	[131]	
 	Emek	SEA, WA	[119, 154, 160, 165]	
 	Enteritidis	AF,CAM, EA, EU, NAM, SA, SEA, WA	 [2, 11, 15, 22, 33, 34, 42, 69, 88, 91, 94, 119, 123, 134, 135, 160, 165, 172, 174, 197, 212, 218]	
	Gallinarum	AF, EA, EU	[123, 172, 209]	
	Gaminarum	EU	[134]	
	Haardt	NAM	[94]	
	Haifa	AF, EA, SEA	[11, 119, 209]	
 	Hadar	EA, EU, NAM, SEA	[40, 91, 119, 135, 161, 165, 172, 173, 175, 212]	
	Hato	EU	[172]	
	Havana	EA, SEA	[11, 119]	
 	Heidelberg	EA, EU, NAM, SEA, WA	[8, 40, 91, 119, 123, 134, 154, 161, 173, 175, 182, 212]	
	Houten	SEA	[119]	
	Indiana	EU	[212]	
	Infantis	AF, EA, EU, NAM, SEA, WA	 [2, 11, 25, 69, 91, 94, 119, 172, 173, 207, 209, 212, 218]	
	Isangi	EA	[11]	
	Istanbul	EA	[11]	
 	Java	ME	[154]	
	Javiana	SEA	[119, 160]	
	Jerusalem	EU	[134]	
	Kedougou	NAM	[218]	
 	Kentucky	NAM, SEA, WA	[25, 40, 91, 119, 134, 154, 162, 174]	
 	Livingston	EA,EU, SEA, WA	[119, 135, 154, 218]	
	London	EU, SEA	[119, 172]	
 	Mbandaka	EA. EU, NAM, SEA, WA	[40, 91, 119, 123, 134, 154, 172, 175, 207, 212, 218]	
	Meleagridis	EU,SEA	[42, 119]	
	Minnesota	WA	[154]	
	Mons	EA	[11]	
 	Montevideo	EA, EU, NAM, SEA, WA	[11, 14, 69, 91, 94, 119, 154, 172]	
	Muenster	NAM	[94]	
	Muenchen	NAM	[134]	
 	Newport	EA, EU, NAM	[11, 207, 218]	
	Nienstedten	NAM	[91]	
	Ohio	EA, EU, NAM, SEA	[119, 134, 135, 174]	
	Oranienburg	EU, NAM	[91, 218]	
	Othmarschen	EA	[11]	
	Ouakam	NAM	[218]	
	Paratyphi B	EU, WA	[2, 154, 207, 212]	
	Pullorum	EU	[42]	
	Rissen	EU, SEA,	[165, 218]	
	Roan	WA	[154]	
 	Saintpaul	NAM, WA	[25, 154]	
	Sarajane	WA	[33]	
	Schwarzengrund	EA, NAM	[40, 94, 135, 173]	
	Senftenberg	EA, EU, NAM, SEA	[14, 42, 94, 119, 123, 134, 174, 218]	
	Singapore	SEA	[160]	
	Sofia	SEA, WA	[119, 154]	
	Southampton	SEA	[160]	
	Stanley	SEA	[119]	
	Tennessee	EA,EU	[11, 88, 134, 218]	
	Thomasville	NAM	[94]	
	Thompson	EA, NAM, SEA, WA	[11, 14, 33, 40, 91, 94, 119, 174]	
 	Typhimurium	CAM, EA, EU, NAM, SEA	[34, 40, 69, 91, 94, 119, 134, 151, 160, 161, 164, 172, 174, 175, 182, 207, 212, 218]	
	Virchow	EA, EU, SEA, WA	[2, 11, 34, 119, 165, 172, 212]	
	Virginia	AF	[209]	
 	Weltevreden	SEA	 [119, 160, 165]	
	Worthington	EU, NAM	[94, 207]	
turkey & ducks	4,5,12:i:-	NAM	[37]	
	4,12:b:-	EU	[169]	
	6,7:-:-	EU	[169]	
	Agona	EU, NAM	 [64, 91, 113, 162, 169]	
	Albany	NAM	[113]	
	Anatum	EU, NAM	[42, 91, 113, 162, 169]	
	Arizonae	NAM	[129]	
	Assinine	EA	[208]	
	Berta	EU	[169]	
	Braenderup	SEA	[160]	
	Brandenburg	EU	[169]	
 	Bredney	NAM	[113, 161]	
	Broughton	NAM	[113]	
	Bovismorbificans	EU, SEA	[160, 169]	
	Derby	EA, EU, SEA	[160, 169, 208]	
	Dublin	SEA	[160]	
	Duiseburg	EU	[169]	
	Dusseldorf	EA	[208]	
	Emek	EU	[146]	
 	Enteritidis	EU, NAM	[42, 81, 138, 156, 162, 169]	
	Hadar	EA, EU, NAM	[9, 11, 91, 113, 162, 169, 208]	
	Havana	EU, NAM	[113, 169]	
 	Heidelberg	EU, NAM	[8, 91, 113, 158, 162, 169]	
	Indiana	EA, EU, NAM	[96, 113, 169, 208]	
	Infantis	EU	[169]	
	Javiana	SEA	[160]	
 	Kentucky	NAM	 [91, 113]	
	Lexington	SEA,EU	[160, 169]	
	Livington	EU	[169]	
	Lome	SEA	[160]	
	Mbandaka	NAM	[91, 113]	
	Meleagridis	EU	[42]	
	Montevideo	EA, EU, NAM	[10, 169, 208]	
 	Muenster	EU, NAM	 [91, 113, 158, 169]	
	New-haw	EU	[131]	
 	Newport	EA, EU, NAM, SEA	[42, 160, 162, 169, 208]	
	Nienstedten	NAM	[91]	
	Ohio	NAM	[10]	
	Paratyphi B	EU	[146]	
	Potsdam	EA	[208]	
	Reading	NAM	[113]	
	Rissen	EU, SEA	[103, 169]	
	Rough	EU	[169]	
	Rubislaw	AF	[46]	
 	Saintpaul	EU, NAM	[42, 113, 162, 169]	
 	Schwarzengrund	EA, NAM	[113, 161, 208]	
 	Senftenberg	EU, NAM, SEA	[42, 113, 160, 162, 169]	
	Tennessee	EU	[169]	
 	Typhimurium	EA, EU, NAM, SEA	[42, 78, 91, 109, 113, 146, 160, 162, 169, 178, 208]	
	Thompson	NAM	[91]	
	Uganda	EU	[169]	
	Virchow	AF	[46]	
	Wagenia	SEA	[160]	
	Weltevreden	SEA	[160] 	
	Weybridge	EU	[131]	
	Worthington	NAM	[158]	
				
non-domestic birds				
 	4,12:d:-	AU	[114]	
	6,14:Z4,Z23:	EU	[144]	
 	Aberdeen	SAM	[192]	
 	Adelaide	AU, EU	[114, 178]	
 	Agona	AU	[114]	
 	Alabama	NAM	[110]	
 	Anatum	AU, NAM	[110, 114]	
 	Bahrenfeld	AU	[114]	
	Blockley	SEA	[119]	
 	Bovismorbificans	AU	[114]	
 	Braenderup	NAM	[116]	
 	Brandenburg	AU, EU	[43, 178]	
 	Chester	AU	[114]	
 	Coleypark	AU	[114]	
 	Derby	AU	[114]	
 	Enteritidis	AN, EU	[42, 144, 167, 178]	
 	Give	AU	[114]	
 	Havana	AF, AN, AU, EU	[114, 167, 178]	
 	Houtnae	EU	[144]	
	Hvittingfoss	SEA	[119]	
 	II Wandsbek	AU	[114]	
 	Indiana	EU	[139]	
 	Infantis	AU, EU, NAM	[110, 114, 168]	
 	Javiana	AU	[114]	
 	Johannesberg	NAM	[202]	
	Kedougou	EU	[168]	
	Lexington	SEA	[119]	
 	Litchfield	AU	[114]	
	Liverpool	EU	[171]	
 	Livingston	AU	[114]	
	Matopeni	SEA	[119]	
	Mision	AF	[46]	
 	Montevideo	NAM	[202]	
 	Muenchen	EU, AU	[114, 144]	
	Muenster	NAM	[177]	
 	Newington	AU	[114]	
 	Newport	AU, AN, EU, SAM	[114,, 167, 168, 178, 192]	
 	Ohio	AU, NAM	[114, 202]	
 	Oranienburg	AU	[114]	
 	Orion	AU	[114]	
 	Panama	AU, SAM	[114, 192]	
 	Paratyphi B 	NAM, SEA	[110, 119]	
 	Rubislaw	AF, SAM	[46, 192]	
 	Saintpaul	AU, EU	[114, 178]	
 	Senftenberg	AU, NAM	[114, 116]	
 	Singapore	AU	[114]	
	Stanley	SEA	[119]	
 	Tennessee	AU	[114]	
 	Thompson	SAM	[192]	
	Toricada	SEA	[119]	
 	Typhimurium	AF, AN, AU, EU, NAM, SAM, SEA	[24, 42, 72, 110, 114, 119, 140, 144, 168, 171, 179]	
 	Virchow	EU	[178]	
	Weltevreden	SEA	[119]	
				
Reptiles & Amphibians				
	45ab:(g)Z5a:-	CAM	[128]	
	-:Z4,Z32:-	CAM	[128]	
	ll:Z4,Z32:-	CAM	[128]	
 	50,1,2,3:Z4,Z24: -	CAM	[128]	
 	Aberdeen	EU	[170]	
 	Abony	EU	[29, 170]	
 	Adelaide	EU	[169]	
 	Agama	AF 	 [46, 131]	
	Agona	EU, NAM, SAM	[39, 66, 72]	
 	Akuafo	AF	[131]	
	Alachua	AF, EA	[140, 155]	
 	Amherstiana	SAM	[72]	
 	Amsterdam	EA	[155]	
 	Anatum	EU, NAM, SAM, SEA	[29, 39, 66, 72, 206]	
 	Apapa	EU	[66]	
 	Augustenborg	EU	[89]	
 	Bahrenfeld	EU	[170]	
 	Baildon	EU	[170]	
 	Ball	EU	[170]	
 	Bardo	EA	[155]	
 	Bareilly	EA, EU,  NAM	[121, 155, 170]	
 	Beaudesert	EA	[155]	
 	Bellville	NAM	[39]	
 	Bern	CAM	[128]	
	Berta	EU, NAM	[39, 66]	
 	Bleadon	AF	[131]	
 	Blockley	NAM	[39]	
	Blukwa	EU	[66, 170]	
 	Bokanjac	AF	[131]	
 	Bonaire	CAM	[128]	
 	Bovismorbificans	EU	[170]	
 	Braenderup	EA	[155]	
	Bredeney	AF, EU, NAM	[39, 140]	
 	Calvinia	AF	[131]	
 	Caracas	EU, SAM	[66, 72]	
 	Carrau	CAM, EU	[89, 128]	
 	Ceyco	EU	[66]	
 	Chameleon	CAM	[128]	
	Chandans	EU	[29]	
	Charity	AF	[131]	
	Chersina	AF	[131]	
	Chichiri	EU	[66]	
 	Clifton	AF	[131]	
 	Degania	CAM	[128]	
 	Denver	CAM	[128]	
	Durban	EU	[66]	
 	Durham	AF	[46]	
	Duval	EU	[66]	
 	Eastbourne	AF, NAM	[46, 121]	
	Ebrie	EU	[66]	
 	Elisabethville	AF	[46]	
	Enteritidis	EA, EU, NAM, SAM	[39, 89, 155, 170, 192]	
 	Epicrates	AF	[131]	
 	Florida	NAM, EA	[39, 155]	
 	Fluntern	EU	[66]	
 	Fresno	EU	[170]	
 	Friedrichsfelde	EU	[66]	
	Galiema	NAM	[39]	
 	Ghana	AF	[131]	
 	Give	NAM	[39]	
	Glostrup	CAM	[128]	
 	Gwoza	AF	[46, 131]	
 	Hadar	NAM, SEA	[39, 206]	
 	Haifa	EA	[155]	
	Heidelberg	EU	[89]	
 	Heron	AF	[131]	
	Herzliya	EU	[29]	
 	Honelis	AF	[131]	
 	Horsham	EA	[155]	
 	Houten	CAM	[128]	
 	Hull	NAM	[39]	
 	Hvittingfoss	EA, SEA	[155, 206]	
 	I 4,[5],12,i:-	NAM	[128]	
 	Infantis	EU, NAM	[39, 169]	
 	Isangi	AF	[140]	
 	Israel	EU	[66]	
 	Istanbul	EU	[170]	
 	Jacksonville	NAM	[39]	
 	Jodhpur	EU	[89]	
	Johannesburg	AF	[140]	
 	Kaapstadt	AF	[46]	
 	Kapemba	EU	[66]	
 	Kentucky	EA	[155]	
	Kibi	EU	[29, 169]	
 	Kingston	AF	[46]	
 	Kisarawe	EA, EU	[89, 155]	
 	Kottbus	EU	[66]	
	Kuntair	EU	[66]	
 	Langford	EU	[131]	
	Lawra	EU	[89]	
	Legon	AF	[131]	
	Lethon	EU	[131]	
	Lindrick	AF	[131]	
 	Litchfield	SAM	[72]	
 	Lohbruegge	CAM, SEA	[119, 128]	
 	London	EU,SAM	[72, 170]	
 	Luciana	NAM	[39]	
 	Macclesfield	EU	[29]	
	Madelia	CAM	[128]	
 	Magumeri	AF	[46, 131]	
 	Manhattan	EU, NAM	[39, 89]	
	Manila	NAM	[39]	
 	Marylebone	EU	[131]	
 	Mbandaka	EU	[170]	
	Memphis	EU	[66]	
 	Mendoza	SAM	[72]	
 	Miami	CAM, NAM	[39, 128]	
 	Midway	EU	[66]	
 	Millesi	AF	[140]	
	Minnesota	EA	[155]	
 	Moero	AF	[46]	
 	Moers	NAM	[170]	
	Montevideo	CAM, EA, EU, NAM, SAM	[39, 66, 89, 121, 128, 155, 170, 192]	
 	Mosselbay	AF	[131]	
 	Muenchen	EA, EU, NAM, SEA	[39, 66, 155, 170, 206]	
 	Muenster	NAM	[39]	
 	Mutade	AF	[140]	
	Nagoya	CAM	[128]	
	Namib	AF	[131]	
 	Nashua	EU	[131]	
 	Newport	CAM, EA, EU, NAM, SAM, SEA	[39, 72, 89, 128, 155, 169, 192]	
	Nima	EU, NAM	[121, 170]	
 	Nottingham	EU	[89]	
 	Oakland	EU	[89]	
 	Ohio	NAM	[39]	
 	Oranienburg	AF, NAM	[39, 121, 140]	
 	Oslo	EU	[169]	
	Othmarschen	EA, EU	[66, 89, 155]	
	Oxford	EU	[131]	
 	Oysterbeds	AF	[131]	
 	Panama	CAM, EA, EU, SAM, SEA	[72, 89, 128, 155, 192]	
 	Paratyphi B 	CAM	[128]	
 	Parera	CAM	[128]	
 	Pomona	AF	[140]	
 	Poona	EA	[155]	
 	Potengi	EA	[155]	
 	Potsdam	EA, EU, NAM	[29, 39, 155]	
 	Quinhon	EU	[170]	
 	Ramatgan	EU	[89]	
 	Reading	EU	[66]	
 	Rechovot	EU	[29]	
 	Redlands	EU	[170]	
	Rissen	EA,  NAM, SAM	[39, 72, 155]	
	Roggeveld	AF	[131]	
 	Rowbarton	AF	[131]	
 	Rubislaw	CAM, NAM, SAM	[39, 72, 128]	
	Saintpaul	NAM	[39]	
 	Sarajane	EU	[131]	
 	San Diego	CAM	[128]	
 	Schwarzengrund	EA	[155]	
 	Senftenberg	EU, NAM	[39, 66]	
 	Simi	EU	[89]	
	Singapore	EU	[170]	
 	Sofia	EU	[131]	
 	Sokode	EU	[29]	
 	Solt	AF	[140]	
 	Spartel	EU	[29]	
	Stanley	EA, NAM	[39, 155]	
	Subsp. II	AF, EA, EU	[29, 66, 140, 155]	
 	Subsp. IIIa	EA, EU	[29, 66, 89, 155]	
	Subsp. IIIb	AF, EA, EU	[66, 89, 140, 155, 170, 190]	
 	Subsp. IV	EA, EU	[66, 89, 155]	
 	Sundsvall	EA, EU	[89, 155]	
	Szentes	EU	[29]	
	Takoradi	EU	[46, 131]	
	Tamale	AF	[131]	
 	Tauton	EU	[131]	
 	Tennessee	NAM	[39]	
 	Thompson	CAM, NAM	[39, 128]	
	Trimdon	EU	[66]	
	Typhimurium	AF, NAM, SAM	[39, 72, 140]	
 	Uphill	EU	[131]	
	Veneziana	EU	[66]	
 	Verity	EU	[131]	
 	Virchow	AF, SEA	[192, 206]	
 	Virginia	AF	[140]	
 	Wassenaar	CAM	[128]	
	Weltevreden	EU	[170]	
	Weston	EU	[131]	
	Zadar	EU	[131]	
  Fish tanks				
 	Newport	AF	[192]	
	Panama	AF	[192]	
	Paratyphi B	AU, EU,NAM	[23, 50, 87, 153, 183, 193]	
	Virchow	AF	[192]	
Invertebrates2				
	Eimsbuettel	EU	[52]	
	Enteritidis	EU	[56, 100, 149, 163, 199]	
	Indiana	EU	[199]	
	Infantis	EU	[95]	
	Montevideo	NAM	[51]	
	Paratyphi B	EU, SA	[101, 198]	
	Typhimurium	EU, NAM	[12, 115, 126, 191]	
				
	Typhi*	NAM	[41]	
				
				
				

1Countries/Geographic Regions: EU=Europe; NAM=North America; CAM=Central America; SAM= South America; EA=East Asia; SEA= South East Asia; WA= West Asia; SA=South Asia; ME=Middle East; AF=Africa; AU=Australia; AN=Antarctica;

2 Some of these papers report experimental infections, but articles  are listed for completeness
* Salmonella Typhi reference added for completeness
REFERENCES
[1]	Akiba M., Nakaoka Y., Kida M., Ishioka Y., Sameshima T., Yoshii N., Nakazawa M., Uchida I., Terakado N., Changes in antimicrobial susceptibility in a population of Salmonella enterica serovar Dublin isolated from cattle in Japan from 1976 to 2005, J. Antimicrob. Chemother. (2007) 60:1235-1242.
[2]	al-Nakhli H.M., al-Ogaily Z.H., Nassar T.J., Representative Salmonella serovars isolated from poultry and poultry environments in Saudi Arabia, Rev. Sci. Tech. (1999) 18:700-709.
[3]	Alam M.J., Renter D., Taylor E., Mina D., Moxley R., Smith D., Antimicrobial susceptibility profiles of Salmonella enterica serotypes recovered from pens of commercial feedlot cattle using different types of composite samples, Curr. Microbiol. (2009) 58:354-359.
[4]	Alam M.J., Renter D.G., Ives S.E., Thomson D.U., Sanderson M.W., Hollis L.C., Nagaraja T.G., Potential associations between fecal shedding of Salmonella in feedlot cattle treated for apparent respiratory disease and subsequent adverse health outcomes, Vet. Res. (2009) 40:2.
[5]	Alemayehu D., Molla B., Muckle A., Prevalence and antimicrobial resistance pattern of Salmonella isolates from apparently healthy slaughtered cattle in Ethiopia, Trop. Anim. Health Prod. (2003) 35:309-319.
[6]	Alvseike O., Skjerve E., Prevalence of a Salmonella subspecies diarizonae in Norwegian sheep herds, Prev .Vet. Med. (2002) 52:277-285.
[7]	Anderson G.D., Lee D.R., Salmonella in horses: a source of contamination of horsemeat in a packing plant under federal inspection, Appl. Environ. Microbiol. (1976) 31:661-663.
[8]	Andrysiak A.K., Olson A.B., Tracz D.M., Dore K., Irwin R., Ng L.K., Gilmour M.W., Genetic characterization of clinical and agri-food isolates of multi drug resistant Salmonella enterica serovar Heidelberg from Canada, BMC Microbiol. (2008) 8:89.
[9]	Anonymous, From the Centers for Disease Control. Salmonella hadar associated with pet ducklings--Connecticut, Maryland, and Pennsylvania, 1991, J. Am. Vet. Med. Assoc (1992) 267:2011.
[10]	Anonymous, Three outbreaks of salmonellosis associated with baby poultry from three hatcheries--United States, 2006, MMWR Morb. Mortal. Wkly Rep. (2007) 56:273-276.
[11]	Asai T., Esaki H., Kojima A., Ishihara K., Tamura Y., Takahashi T., Antimicrobial resistance in Salmonella isolates from apparently healthy food-producing animal from 2000 to 2003: the first stage of Japanese veterinary antimicrobial resistance monitoring (JVARM), J. Vet. Med. Sci. (2006) 68:881-884.
[12]	Ash N., Greenberg B., Vector potential of the German cockroach (Dictyoptera: Blattellidae) in dissemination of Salmonella enteritidis serotype Typhimurium, J. Med. Entomol. (1980) 17:417-423.
[13]	Bagcigil A.F., Ikiz S., Dokuzeylu B., Basaran B., Or E., Ozgur N.Y., Fecal shedding of Salmonella spp. in dogs, J. Vet. Med. Sci. (2007) 69:775-777.
[14]	Bailey J.S., Stern N.J., Fedorka-Cray P., Craven S.E., Cox N.A., Cosby D.E., Ladely S., Musgrove M.T., Sources and movement of Salmonella through integrated poultry operations: a multistate epidemiological investigation, J. Food Prot. (2001) 64:1690-1697.
[15]	Barbour E.K., Jurdi L.H., Talhouk R., Qatanani M., Eid A., Sakr W., Bouljihad M., Spasojevic R., Emergence of Salmonella enteritidis outbreaks in broiler chickens in the Lebanon: epidemiological markers and competitive exclusion control, Rev. Sci .Tech. (1999) 18:710-718.
[16]	Beach J.C., Murano E.A., Acuff G.R., Serotyping and antibiotic resistance profiling of Salmonella in feedlot and nonfeedlot beef cattle, J. Food Prot. (2002) 65:1694-1699.
[17]	Begg A.P., Johnston K.G., Hutchins D.R., Edwards D.J., Some aspects of the epidemiology of equine salmonellosis, Aust. Vet. J. (1988) 65:221-223.
[18]	Beloeil P.A., Chauvin C., Proux K., Madec F., Fravalo P., Alioum A., Impact of the Salmonella status of market-age pigs and the pre-slaughter process on Salmonella caecal contamination at slaughter, Vet. Res. (2004) 35:513-530.
[19]	Beloeil P.A., Fravalo P., Fablet C., Jolly J.P., Eveno E., Hascoet Y., Chauvin C., Salvat G., Madec F., Risk factors for Salmonella enterica subsp. enterica shedding by market-age pigs in French farrow-to-finish herds, Prev. Vet. Med. (2004) 63:103-120.
[20]	Belton D., Gorton R., Ragg J. , Carriage of salmonellae and yersiniae by New Zealand hedgehogs. , Surveill (1997) 24:9-10.
[21]	Berge A.C., Moore D.A., Sischo W.M., Prevalence and antimicrobial resistance patterns of Salmonella enterica in preweaned calves from dairies and calf ranches, Am. J. Vet. Res. (2006) 67:1580-1588.
[22]	Berghold C., Kornschober C., Weber S., A regional outbreak of S. Enteritidis phage type 5, traced back to the flocks of an egg producer, Austria, Euro Surveill. (2003) 8:195-198.
[23]	Bertrand S., Rimhanen-Finne R., Weill F.X., Rabsch W., Thornton L., Perevoscikovs J., van Pelt W., Heck M., Salmonella infections associated with reptiles: the current situation in Europe, Euro Surveill. (2008) 13:pii=1890
[24]	Besser T.E., Gay C.C., Gay J.M., Hancock D.D., Rice D., Pritchett L.C., Erickson E.D., Salmonellosis associated with S typhimurium DT104 in the USA, Vet. Rec. (1997) 140:75.
[25]	Bhargava K.K., O'Neil J.B., Prior M.G., Dunkelgod K.E., Incidence of Salmonella contamination in broiler chickens in Saskatchewan, Can. J. Comp. Med. (1983) 47:27-32.
[26]	Bischoff K.M., Edrington T.S., Callaway T.R., Genovese K.J., Nisbet D.J., Characterization of antimicrobial resistant Salmonella Kinshasa from dairy calves in Texas, Lett. Appl. Microbiol. (2004) 38:140-145.
[27]	Blau D.M., McCluskey B.J., Ladely S.R., Dargatz D.A., Fedorka-Cray P.J., Ferris K.E., Headrick M.L., Salmonella in dairy operations in the United States: prevalence and antimicrobial drug susceptibility, J. Food Prot. (2005) 68:696-702.
[28]	Branham L.A., Carr M.A., Scott C.B., Callaway T.R., E. coli O157 and Salmonella spp. in white-tailed deer and livestock, Curr. Issues Intest. Microbiol. (2005) 6:25-29.
[29]	Briones V., Tellez S., Goyache J., Ballesteros C., del Pilar Lanzarot M., Dominguez L., Fernandez-Garayzabal J.F., Salmonella diversity associated with wild reptiles and amphibians in Spain, Environ. Microbiol. (2004) 6:868-871.
[30]	Callaway T.R., Keen J.E., Edrington T.S., Baumgard L.H., Spicer L., Fonda E.S., Griswold K.E., Overton T.R., VanAmburgh M.E., Anderson R.C., Genovese K.J., Poole T.L., Harvey R.B., Nisbet D.J., Fecal prevalence and diversity of Salmonella species in lactating dairy cattle in four states, J. Dairy Sci. (2005) 88:3603-3608.
[31]	Cantor G.H., Nelson S., Jr., Vanek J.A., Evermann J.F., Eriks I.S., Basaraba R.J., Besser T.E., Salmonella shedding in racing sled dogs, J. Vet. Diagn. Invest. (1997) 9:447-448.
[32]	Caraway C.T., Scott A.E., Roberts N.C., Hauser G.H., Salmonellosis in sentry dogs, J. Am. Vet. Med. Assoc. (1959) 135:599-602.
[33]	Carli K.T., Eyigor A., Caner V., Prevalence of Salmonella serovars in chickens in Turkey, J. Food Prot. (2001) 64:1832-1835.
[34]	Carraminana J.J., Rota C., Agustin I., Herrera A., High prevalence of multiple resistance to antibiotics in Salmonella serovars isolated from a poultry slaughterhouse in Spain, Vet. Microbiol. (2004) 104:133-139.
[35]	Casebolt D.B., Schoeb T.R., An outbreak in mice of salmonellosis caused by Salmonella enteritidis serotype enteritidis, Lab Anim. Sci. (1988) 38:190-192.
[36]	CDC, African pygmy hedgehog-associated salmonellosis--Washington, 1994, MMWR Morb. Mortal. Wkly Rep. (1995) 44:462-463.
[37]	CDC, Three outbreaks of salmonellosis associated with baby poultry from three hatcheries--United States, 2006, MMWR Morb. Mortal. Wkly Rep. (2007) 56:273-276.
[38]	Chadfield M., Skov M., Christensen J., Madsen M., Bisgaard M., An epidemiological study of Salmonella enterica serovar 4, 12:b:- in broiler chickens in Denmark, Vet Microbiol. (2001) 82:233-247.
[39]	Chambers D.L., Hulse A.C., Salmonella serovars in the herpetofauna of Indiana County, Pennsylvania, Appl. Environ. Microbiol. (2006) 72:3771-3773.
[40]	Chambers J.R., Bisaillon J.R., Labbe Y., Poppe C., Langford C.F., Salmonella prevalence in crops of Ontario and Quebec broiler chickens at slaughter, Poult. Sci. (1998) 77:1497-1501.
[41]	Cirillo V.J., "Winged sponges": houseflies as carriers of typhoid fever in 19th- and early 20th-century military camps, Perspect. Biol .Med. (2006) 49:52-63.
[42]	Clarenburg A., Salmonella types isolated during 1946-1952 from man and animals in the Netherlands (National Salmonella Centre), J. Appl. Microbiol. (1953) 16:10-15.
[43]	Clark R.G., Fenwick S.G., Nicol C.M., Marchant R.M., Swanney S., Gill J.M., Holmes J.D., Leyland M., Davies P.R., Salmonella Brandenburg - emergence of a new strain affecting stock and humans in the South Island of New Zealand, N. Z. Vet. J. (2004) 52:26-36.
[44]	Clegg F.G., Chiejina S.N., Duncan A.L., Kay R.N., Wray C., Outbreaks of Salmonella newport infection in dairy herds and their relationship to management and contamination of the environment, Vet. Rec. (1983) 112:580-584.
[45]	Clyde V.L., Ramsay E.C., Bemis D.A., Fecal shedding of Salmonella in exotic felids, J. Zoo Wildl. Med. (1997) 28:148-152.
[46]	Collard P.a.S., R., Serotypes of Salmonella at Ibadan, Nigeria, with Special Note of the New Serotypes Isolated in Nigeria J. Infect. Dis. (1960) 106: 270-275 
[47]	Compton J.A., Baney J.A., Donaldson S.C., Houser B.A., San Julian G.J., Yahner R.H., Chmielecki W., Reynolds S., Jayarao B.M., Salmonella Infections in the common raccoon (Procyon lotor) in Western Pennsylvania, J. Clin. Microbiol. (2008) 46:3084-3086.
[48]	Cormican M., DeLappe N., O'Hare C., Doran G., Morris D., Corbett-Feeney G., Fanning S., Daly M., Fitzgerald M., Moore J., Salmonella enterica serotype Bredeney: antimicrobial susceptibility and molecular diversity of isolates from Ireland and Northern Ireland, Appl. Environ. Microbiol. (2002) 68:181-186.
[49]	Corrier D.E., Purdy C.W., DeLoach J.R., Effects of marketing stress on fecal excretion of Salmonella spp in feeder calves, Am. J. Vet. Res. (1990) 51:866-869.
[50]	Cox RA R.J., Salmonella java from tropical fish tanks, Commun.Dis. Report. (1981) 31:4.
[51]	Crumrine M.H., Foltz V.D., Harris J.O., Transmission of Salmonella montevideo in wheat by stored-product insects, Appl. Microbiol. (1971) 22:578-580.
[52]	Cuturic S., Topolnik E., [Bread beetle (Stegobium paniceum L.) as Salmonella vector in fodder and fodder mixture (author's transl)], Zentralbl. Bakteriol. Orig. A. (1975) 232:545-548.
[53]	Dargatz D.A., Fedorka-Cray P.J., Ladely S.R., Ferris K.E., Survey of Salmonella serotypes shed in feces of beef cows and their antimicrobial susceptibility patterns, J. Food Prot. (2000) 63:1648-1653.
[54]	Dargatz D.A., Fedorka-Cray P.J., Ladely S.R., Kopral C.A., Ferris K.E., Headrick M.L., Prevalence and antimicrobial susceptibility of Salmonella spp. isolates from US cattle in feedlots in 1999 and 2000, J. Appl. Microbiol. (2003) 95:753-761.
[55]	Davies P.R., Morrow W.E., Jones F.T., Deen J., Fedorka-Cray P.J., Harris I.T., Prevalence of Salmonella in finishing swine raised in different production systems in North Carolina, USA, Epidemiol. Infect. (1997) 119:237-244.
[56]	Davies R.H., Wray C., Contribution of the lesser mealworm beetle (Alphitobius diaperinus) to carriage of Salmonella enteritidis in poultry, Vet. Rec. (1995) 137:407-408.
[57]	Davies R.H., Wray C., Mice as carriers of Salmonella enteritidis on persistently infected poultry units, Vet. Rec. (1995) 137:337-341.
[58]	Davies R.H., Dalziel R., Gibbens J.C., Wilesmith J.W., Ryan J.M., Evans S.J., Byrne C., Paiba G.A., Pascoe S.J., Teale C.J., National survey for Salmonella in pigs, cattle and sheep at slaughter in Great Britain (1999-2000), J. Appl. Microbiol. (2004) 96:750-760.
[59]	Davison H.C., Smith R.P., Pascoe S.J., Sayers A.R., Davies R.H., Weaver J.P., Kidd S.A., Dalziel R.W., Evans S.J., Prevalence, incidence and geographical distribution of serovars of Salmonella on dairy farms in England and Wales, Vet. Rec. (2005) 157:703-711.
[60]	Dennis S.M., Armstrong J.M., Ovine Abortion Due to Salmonella Typhimurium in Western Australia, Aust. Vet. J. (1965) 41:178-181.
[61]	Dietz H.H., Chriel M., Andersen T.H., Jorgensen J.C., Torpdahl M., Pedersen H., Pedersen K., Outbreak of Salmonella Dublin-associated abortion in Danish fur farms, Can. Vet. J. (2006) 47:1201-1205.
[62]	Donahue J.M., Emergence of antibiotic-resistant Salmonella agona in horses in Kentucky, J. Am. Vet. Med. Assoc. (1986) 188:592-594.
[63]	Dorn C.R., Coffman J.R., Schmidt D.A., Garner H.E., Addison J.B., McCune E.L., Neutropenia and salmonellosis in hospitalized horses, J. Am. Vet. Med. Assoc. (1975) 166:65-67.
[64]	Douris A., Fedorka-Cray P.J., Jackson C.R., Characterization of Salmonella enterica serovar Agona slaughter isolates from the animal arm of the National Antimicrobial Resistance Monitoring System-Enteric Bacteria (NARMS): 1997 through 2003, Microb. Drug. Resist. (2008) 14:55-63.
[65]	Duffy L., Barlow R., Fegan N., Vanderlinde P., Prevalence and serotypes of Salmonella associated with goats at two Australian abattoirs, Lett. Appl. Microbiol. (2009) 48:193-197.
[66]	Ebani V.V., Cerri D., Fratini F., Meille N., Valentini P., Andreani E., Salmonella enterica isolates from faeces of domestic reptiles and a study of their antimicrobial in vitro sensitivity, Res. Vet. Sci. (2005) 78:117-121.
[67]	Edwards P.R., Salmonella aertrycke in Colitis of Foals, J. Infect. Dis. (1934) 54:85-90.
[68]	Ernst N.S., Hernandez J.A., MacKay R.J., Brown M.P., Gaskin J.M., Nguyen A.D., Giguere S., Colahan P.T., Troedsson M.R., Haines G.R., Addison I.R., Miller B.J., Risk factors associated with fecal Salmonella shedding among hospitalized horses with signs of gastrointestinal tract disease, J. Am.Vet. Med. Assoc. (2004) 225:275-281.
[69]	Esaki H., Morioka A., Ishihara K., Kojima A., Shiroki S., Tamura Y., Takahashi T., Antimicrobial susceptibility of Salmonella isolated from cattle, swine and poultry (2001-2002): report from the Japanese Veterinary Antimicrobial Resistance Monitoring Program, J. Antimicrob. Chemother. (2004) 53:266-270.
[70]	Esaki H., Morioka A., Kojima A., Ishihara K., Asai T., Tamura Y., Izumiya H., Terajima J., Watanabe H., Takahashi T., Epidemiological characterization of Salmonella Typhimurium DT104 prevalent among food-producing animals in the Japanese veterinary antimicrobial resistance monitoring program (1999-2001), Microbiol.Immunol. (2004) 48:553-556.
[71]	Evans S.J., Davies R.H., Binns S.H., Liebana E., Jones T.W., Millar M.F., Threlfall E.J., Ward L.R., Hopkins K.L., Mackay P.H., Gayford P.J., Multiple antimicrobial resistant Salmonella enterica serovar Paratyphi B variant Java in cattle: a case report, Vet. Rec. (2005) 156:343-346.
[72]	Everard C.O., Tota B., Bassett D., Ali C., Salmonella in wildlife from Trinidad and Grenada, W.I, J. Wildl. Dis. (1979) 15:213-219.
[73]	Farzan A., Friendship R.M., Dewey C.E., Muckle A.C., Gray J.T., Funk J., Distribution of Salmonella serovars and phage types on 80 Ontario swine farms in 2004, Can J Vet Res. (2008) 72:1-6.
[74]	Fedorka-Cray P.J., Dargatz D.A., Thomas L.A., Gray J.T., Survey of Salmonella serotypes in feedlot cattle, J. Food Prot. (1998) 61:525-530.
[75]	Fegan N., Vanderlinde P., Higgs G., Desmarchelier P., Quantification and prevalence of Salmonella in beef cattle presenting at slaughter, J. Appl. Microbiol. (2004) 97:892-898.
[76]	Finley R., Reid-Smith R., Ribble C., Popa M., Vandermeer M., Aramini J., The occurrence and anti-microbial susceptibility of Salmonellae isolated from commercially available pig ear pet treats, Zoonoses Public Health. (2008) 55:455-461.
[77]	Finley R., Reid-Smith R., Ribble C., Popa M., Vandermeer M., Aramini J., The occurrence and antimicrobial susceptibility of salmonellae isolated from commercially available canine raw food diets in three Canadian cities, Zoonoses Public Health. (2008) 55:462-469.
[78]	Foley S.L., White D.G., McDermott P.F., Walker R.D., Rhodes B., Fedorka-Cray P.J., Simjee S., Zhao S., Comparison of subtyping methods for differentiating Salmonella enterica serovar Typhimurium isolates obtained from food animal sources, J. Clin. Microbiol. (2006) 44:3569-3577.
[79]	Foley S.L., Lynne A.M., Nayak R., Salmonella challenges: prevalence in swine and poultry and potential pathogenicity of such isolates, J. Anim. Sci. (2008) 86:E149-162.
[80]	Foreyt W.J., Besser T.E., Lonning S.M., Mortality in captive elk from salmonellosis, J .Wildl. Dis. (2001) 37:399-402.
[81]	Frye J.G., Fedorka-Cray P.J., Prevalence, distribution and characterisation of ceftiofur resistance in Salmonella enterica isolated from animals in the USA from 1999 to 2003, Int. J. Antimicrob. Agents. (2007) 30:134-142.
[82]	Fukata T., Naito F., Yoshida N., Yamaguchi T., Mizumura Y., Hirai K., Incidence of Salmonella infection in healthy dogs in Gifu Prefecture, Japan, J. Vet. Med .Sci. (2002) 64:1079-1080.
[83]	Funk J.A., Davies P.R., Gebreyes W., Risk factors associated with Salmonella enterica prevalence in three-site swine production systems in North Carolina, USA, Berl. Munch. Tierarztl. Wochenschr. (2001) 114:335-338.
[84]	Funk J.A., Davies P.R., Nichols M.A., Longitudinal study of Salmonella enterica in growing pigs reared in multiple-site swine production systems, Vet. Microbiol. (2001) 83:45-60.
[85]	Futagawa-Saito K., Hiratsuka S., Kamibeppu M., Hirosawa T., Oyabu K., Fukuyasu T., Salmonella in healthy pigs: prevalence, serotype diversity and antimicrobial resistance observed during 1998-1999 and 2004-2005 in Japan, Epidemiol. Infect. (2008) 136:1118-1123.
[86]	Galland J.C., House J.K., Hyatt D.R., Hawkins L.L., Anderson N.V., Irwin C.K., Smith B.P., Prevalence of Salmonella in beef feeder steers as determined by bacterial culture and ELISA serology, Vet. Microbiol. (2000) 76:143-151.
[87]	Gaulin C., Vincent C., Alain L., Ismail J., Outbreak of Salmonella paratyphi B linked to aquariums in the province of Quebec, 2000, Can. Commun. Dis. Rep. (2002) 28:89-93, 96.
[88]	Geue L., Schluter H., A Salmonella monitoring programme in egg production farms in Germany, Zentralbl. Veterinarmed .B. (1998) 45:95-103.
[89]	Geue L., Loschner U., Salmonella enterica in reptiles of German and Austrian origin, Vet. Microbiol. (2002) 84:79-91.
[90]	Gudmundsdottir S., Hardardottir H., Gunnarsson E., Subtyping of Salmonella enterica serovar typhimurium outbreak strains isolated from humans and animals in Iceland, J. Clin. Microbiol. (2003) 41:4833-4835.
[91]	Guerin M.T., Martin S.W., Darlington G.A., Rajic A., A temporal study of Salmonella serovars in animals in Alberta between 1990 and 2001, Can. J. Vet. Res. (2005) 69:88-99.
[92]	Guilbride P.D., Coyle T.J., Mc A.E., Barber L., Lomax G.D., Some pathogenic agents found in hippopotamus in Uganda, J. Comp. Pathol. (1962) 72:137-141.
[93]	Habrun B., Listes E., Spicic S., Cvetnic Z., Lukacevic D., Jemersic L., Lojkic M., Kompes G., An outbreak of Salmonella Abortusovis abortions in sheep in south Croatia, J. Vet. Med .B. Infect. Dis. Vet. Public Health. (2006) 53:286-290.
[94]	Hacking W.C., Mitchell W.R., Carlson H.C., Sources of Salmonellae in broiler chickens in Ontario, Can. J. Comp. Med. (1978) 42:392-399.
[95]	Hald B., Olsen A., Madsen M., Typhaea stercorea (Coleoptera: Mycetophagidae), a carrier of Salmonella enterica serovar Infantis in a Danish broiler house, J. Econ. Entomol. (1998) 91:660-664.
[96]	Hall M.L., Threlfall E.J., Rowe B., Pinegar J.A., Gibson G.L., Lactose-fermenting Salmonella indiana from turkeys in Britain, Lancet. (1978) 2:1197-1198.
[97]	Handeland K., Refsum T., Johansen B.S., Holstad G., Knutsen G., Solberg I., Schulze J., Kapperud G., Prevalence of Salmonella typhimurium infection in Norwegian hedgehog populations associated with two human disease outbreaks, Epidemiol. Infect. (2002) 128:523-527.
[98]	Handeland K., Nesse L.L., Lillehaug A., Vikoren T., Djonne B., Bergsjo B., Natural and experimental Salmonella Typhimurium infections in foxes (Vulpes vulpes), Vet. Microbiol. (2008) 132:129-134.
[99]	Harbourne J.F., Randall C.J., Luery K.W., Wallace J.G., Salmonella paratyphi B infection in dairy cows. I, Vet. Rec. (1972) 91:112-114.
[100]	Harein P.K., de las Casas E., Pomeroy B.S., York M.D., Salmonella spp. and serotypes of Escherichia coli isolated from the lesser mealworm collected in poultry brooder houses, J. Econ. Entomol. (1970) 63:80-82.
[101]	Hazeleger W.C., Bolder N.M., Beumer R.R., Jacobs-Reitsma W.F., Darkling beetles (Alphitobius diaperinus) and their larvae as potential vectors for the transfer of Campylobacter jejuni and Salmonella enterica serovar paratyphi B variant Java between successive broiler flocks, Appl .Environ .Microbiol. (2008) 74:6887-6891.
[102]	Healing T.D., Salmonella in rodents: a risk to man?, CDR (Lond Engl Rev). (1991) 1:R114-116.
[103]	Hendriksen R.S., Bangtrakulnonth A., Pulsrikarn C., Pornreongwong S., Hasman H., Song S.W., Aarestrup F.M., Antimicrobial resistance and molecular epidemiology of Salmonella Rissen from animals, food products, and patients in Thailand and Denmark, Foodborne Pathog. Dis. (2008) 5:605-619.
[104]	Hirsh D.C., Smith B.P., Pleasure horses as a possible source of Salmonella agona, Am. J. Vet. Res. (1979) 40:1301-1302.
[105]	Hjartardottir S., Gunnarsson E., Sigvaldadottir J., Salmonella in sheep in Iceland, Acta Vet. Scand. (2002) 43:43-48.
[106]	Holzel C., Bauer J., Salmonella spp. in Bavarian liquid pig manure: occurrence and relevance for the distribution of antibiotic resistance, Zoonoses Public Health. (2008) 55:133-138.
[107]	House J.K., Mainar-Jaime R.C., Smith B.P., House A.M., Kamiya D.Y., Risk factors for nosocomial Salmonella infection among hospitalized horses, J. Am. Vet. Med. Assoc. (1999) 214:1511-1516.
[108]	House J.K., Smith B.P., Wildman T.R., Carrigan M.J., Kamiya D.Y., Isolation of Salmonella organisms from the mesenteric lymph nodes of horses at necropsy, J. Am. Vet. Med. Assoc. (1999) 215:507-510.
[109]	Howerth E.W., Salmonellosis in a wild turkey, J. Wildl. Dis. (1985) 21:433-434.
[110]	Hudson C.R., Quist C., Lee M.D., Keyes K., Dodson S.V., Morales C., Sanchez S., White D.G., Maurer J.J., Genetic relatedness of Salmonella isolates from nondomestic birds in Southeastern United States, J. Clin. Microbiol. (2000) 38:1860-1865.
[111]	Hume M.E., Edrington T.S., Looper M.L., Callaway T.R., Genovese K.J., Nisbet D.J., Salmonella genotype diversity in nonlactating and lactating dairy cows, J. Food Prot. (2004) 67:2280-2283.
[112]	Ikeda J.S., Hirsh D.C., Jang S.S., Biberstein E.L., Characteristics of Salmonella isolated from animals at a veterinary medical teaching hospital, Am. J. Vet.Res. (1986) 47:232-235.
[113]	Irwin R.J., Poppe C., Messier S., Finley G.G., Oggel J., A national survey to estimate the prevalence of Salmonella species among Canadian registered commercial turkey flocks, Can. J .Vet. Res. (1994) 58:263-267.
[114]	Iveson J.B., Shellam G.R., Bradshaw S.D., Smith D.W., Mackenzie J.S., Mofflin R.G., Salmonella infections in Antarctic fauna and island populations of wildlife exposed to human activities in coastal areas of Australia, Epidemiol. Infect. (2009) 137:858-870.
[115]	Janssen W.A., Wedberg S.E., The common house roach, Blattella germanica Linn., as a potential vector of Salmonella typhimurium and Salmonella typhosa, Am. J. Trop. Med. Hyg. (1952) 1:337-343.
[116]	Jijon S., Wetzel A., LeJeune J., Salmonella enterica isolated from wildlife at two Ohio rehabilitation centers, J. Zoo Wildl. Med. (2007) 38:409-413.
[117]	Joffe D.J., Schlesinger D.P., Preliminary assessment of the risk of Salmonella infection in dogs fed raw chicken diets, Can. Vet. J. (2002) 43:441-442.
[118]	Jorgensen S.T., Prevalence and molecular epidemiology of antibiotic-resistant Salmonella typhimurium and Salmonella dublin in Danish cattle, Acta Pathol. Microbiol. Immunol. Scand .B. (1983) 91:163-168.
[119]	Joseph P.G., Sivanandan S.P., Yee H.T., Animal Salmonella surveillance in Peninsular Malaysia, 1981-1985, Epidemiol. Infect. (1988) 100:351-359.
[120]	Kane D.W., The prevalence of salmonella infection in sheep at slaughter N. Z. Vet.J. (1979) 27:110-113.
[121]	Kennedy M.E., Salmonella isolations from snakes and other reptiles, Can. J. Comp. Med. (1973) 37:325-326.
[122]	Keymer I.F., Gibson E.A., Reynolds D.J., Zoonoses and other findings in hedgehogs (Erinaceus europaeus): a survey of mortality and review of the literature, Vet. Rec. (1991) 128:245-249.
[123]	Kim A., Lee Y.J., Kang M.S., Kwag S.I., Cho J.K., Dissemination and tracking of Salmonella spp. in integrated broiler operation, J. Vet. Sci. (2007) 8:155-161.
[124]	Kirchner B.K., Dixon L.W., Lentsch R.H., Wagner J.E., Recovery and pathogenicity of several Salmonella species isolated from mice, Lab. Anim. Sci. (1982) 32:506-508.
[125]	Kishima M., Uchida I., Namimatsu T., Osumi T., Takahashi S., Tanaka K., Aoki H., Matsuura K., Yamamoto K., Nationwide surveillance of salmonella in the faeces of pigs in Japan, Zoonoses Public Health. (2008) 55:139-144.
[126]	Klowden M.J., Greenberg B., Salmonella in the American cockroach: evaluation of vector potential through dosed feeding experiments, J. Hyg. (Lond). (1976) 77:105-111.
[127]	Kocabiyik A.L., Cetin C., Dedicova D., Detection of Salmonella spp. in stray dogs in Bursa Province, Turkey: first isolation of Salmonella Corvallis from dogs, J. Vet. Med. B Infect. Dis. Vet. Public Health. (2006) 53:194-196.
[128]	Kourany M., Telford S.R., Lizards in the ecology of salmonellosis in Panama, Appl Environ Microbiol. (1981) 41:1248-1253.
[129]	Kumar M.C., Nivas S.C., Bahl A.K., York M.D., Pomeroy B.S., Studies on natural infection and egg transmission of Arizona hinshawii 7:1,7,8 in turkeys, Avian Dis. (1974) 18:416-426.
[130]	Kunze D.J., Loneragan G.H., Platt T.M., Miller M.F., Besser T.E., Koohmaraie M., Stephens T., Brashears M.M., Salmonella enterica burden in harvest-ready cattle populations from the southern high plains of the United States, Appl. Environ .Microbiol. (2008) 74:345-351.
[131]	Lapage S.P., Taylor, J. Nicewonger, C.R. and Phillips, A.G., New Serotypes of Salmonella identified before 1964 at the Salmonella Reference Laboratory, Colindale, International Journal of Systematic Biology.(1966) 16:253-297.
[132]	Lefebvre S.L., Reid-Smith R., Boerlin P., Weese J.S., Evaluation of the risks of shedding Salmonellae and other potential pathogens by therapy dogs fed raw diets in Ontario and Alberta, Zoonoses Public Health. (2008) 55:470-480.
[133]	Letellier A., Messier S., Pare J., Menard J., Quessy S., Distribution of Salmonella in swine herds in Quebec, Vet. Microbiol. (1999) 67:299-306.
[134]	Liljebjelke K.A., Hofacre C.L., Liu T., White D.G., Ayers S., Young S., Maurer J.J., Vertical and horizontal transmission of salmonella within integrated broiler production system, Foodborne Pathog. Dis. (2005) 2:90-102.
[135]	Limawongpranee S., Hayashidani H., Okatani A.T., Ono K., Hirota C., Kaneko K., Ogawa M., Prevalence and persistence of Salmonella in broiler chicken flocks, J Vet Med Sci. (1999) 61:255-259.
[136]	Long J.R., Finley G.G., Clark M.H., Rehmtulla A.J., Ovine fetal infection due to Salmonella arizonae, Can. Vet. J. (1978) 19:260-263.
[137]	Low J.C., Tennant B., Munro D., Multiple-resistant Salmonella typhimurium DT104 in cats, Lancet. (1996) 348:1391.
[138]	Lukinmaa S., Schildt R., Rinttila T., Siitonen A., Salmonella enteritidis phage types 1 and 4: pheno- and genotypic epidemiology of recent outbreaks in Finland, J. Clin. Microbiol. (1999) 37:2176-2182.
[139]	Luque I., Echeita A., León J., Herrera-León S., Tarradas C., González-Sanz R., Huerta B., Astorga R.J., Salmonella Indiana as a cause of abortion in ewes: Genetic diversity and resistance patterns, Vet. Microbiol. (2009) 134:396-399.
[140]	Madsen M., Prevalence and serovar distribution of Salmonella in fresh and frozen meat from captive Nile crocodiles (Crocodylus niloticus), Int. J. Food Microbiol. (1996) 29:111-118.
[141]	Mair T.S., de Westerlaken L.V., Cripps P.J., Love S., Diarrhoea in adult horses: a survey of clinical cases and an assessment of some prognostic indices, Vet. Rec. (1990) 126:479-481.
[142]	McEvoy J.M., Doherty A.M., Sheridan J.J., Blair I.S., McDowell D.A., The prevalence of Salmonella spp. in bovine faecal, rumen and carcass samples at a commercial abattoir, J. Appl. Microbiol. (2003) 94:693-700.
[143]	Merialdi G., Barigazzi G., Bonilauri P., Tittarelli C., Bonci M., D'Incau M., Dottori M., Longitudinal study of Salmonella infection in Italian farrow-to-finish swine herds, Zoonoses Public Health. (2008) 55:222-226.
[144]	Millan J., Aduriz G., Moreno B., Juste R.A., Barral M., Salmonella isolates from wild birds and mammals in the Basque Country (Spain), Rev. Sci .Tech. (2004) 23:905-911.
[145]	Miller A.S., Salmonellosis in Botswana. I. Incidence in cattle, J Hyg (Lond). (1971) 69:491-496.
[146]	Mitchell T.R., Ridgwell T., The frequency of salmonellae in wild ducks, J. Med. Microbiol. (1971) 4:359-361.
[147]	Molla B., Berhanu A., Muckle A., Cole L., Wilkie E., Kleer J., Hildebrandt G., Multidrug resistance and distribution of Salmonella serovars in slaughtered pigs, J. Vet. Med.B Infect. Dis. Vet. Public Health. (2006) 53:28-33.
[148]	Molla W., Molla B., Alemayehu D., Muckle A., Cole L., Wilkie E., Occurrence and antimicrobial resistance of Salmonella serovars in apparently healthy slaughtered sheep and goats of central Ethiopia, Trop. Anim. Health Prod. (2006) 38:455-462.
[149]	Moro C.V., Fravalo P., Amelot M., Chauve C., Zenner L., Salvat G., Colonization and organ invasion in chicks experimentally infected with Dermanyssus gallinae contaminated by Salmonella Enteritidis, Avian Pathol. (2007) 36:307-311.
[150]	Morse E.V., Duncan M.A., Page E.A., Fessler J.F., Salmonellosis in Equidae: a study of 23 cases, Cornell Vet. (1976) 66:198-213.
[151]	Murphy B.P., Buckley J.F., O'Connor E.M., Gilroy D., Fanning S., Comparison of Salmonella species recovered from Irish liquid milk production holdings with temporal clinical veterinary isolates, Int. J. Hyg. Environ. Health. (2008) 211:283-291.
[152]	Murugkar H.V., Rahman H., Kumar A., Bhattacharyya D., Isolation, phage typing and antibiogram of Salmonella from man and animals in northeastern India, Indian J. Med. Res. (2005) 122:237-242.
[153]	Musto J., Kirk M., Lightfoot D., Combs B.G., Mwanri L., Multi-drug resistant Salmonella Java infections acquired from tropical fish aquariums, Australia, 2003-04, Commun. Dis. Intell. (2006) 30:222-227.
[154]	Nabbut N.H., Barbour E.K., Al-Nakhli H.M., Salmonella species and serotypes isolated from farm animals, animal feed, sewage, and sludge in Saudi Arabia, Bull .World Health Organ. (1982) 60:803-807.
[155]	Nakadai A., Kuroki T., Kato Y., Suzuki R., Yamai S., Yaginuma C., Shiotani R., Yamanouchi A., Hayashidani H., Prevalence of Salmonella spp. in pet reptiles in Japan, J. Vet. Med. Sci. (2005) 67:97-101.
[156]	Nastasi A., Mammina C., Piersante G.P., Robertazzo M., Caruso P., A foodborne outbreak of Salmonella enteritidis vehicled by duck and hen eggs in southern Italy, New Microbiol. (1998) 21:93-96.
[157]	Nauerby B., Pedersen K., Dietz H.H., Madsen M., Comparison of Danish isolates of Salmonella enterica serovar enteritidis PT9a and PT11 from hedgehogs (Erinaceus europaeus) and humans by plasmid profiling and pulsed-field gel electrophoresis, J. Clin. Microbiol. (2000) 38:3631-3635.
[158]	Nayak R., Stewart T., Wang R.F., Lin J., Cerniglia C.E., Kenney P.B., Genetic diversity and virulence gene determinants of antibiotic-resistant Salmonella isolated from preharvest turkey production sources, Int. J. Food Microbiol. (2004) 91:51-62.
[159]	Nollet N., Houf K., Dewulf J., De Kruif A., De Zutter L., Maes D., Salmonella in sows: a longitudinal study in farrow-to-finish pig herds, Vet. Res. (2005) 36:645-656.
[160]	Ogasawara N., Tran T.P., Ly T.L., Nguyen T.T., Iwata T., Okatani A.T., Watanabe M., Taniguchi T., Hirota Y., Hayashidani H., Antimicrobial susceptibilities of Salmonella from domestic animals, food and human in the Mekong Delta, Vietnam, J. Vet. Med. Sci. (2008) 70:1159-1164.
[161]	Oloya J., Theis M., Doetkott D., Dyer N., Gibbs P., Khaitsa M.L., Evaluation of Salmonella occurrence in domestic animals and humans in North Dakota (2000-2005), Foodborne Pathog. Dis. (2007) 4:551-563.
[162]	Oloya J., Doetkott D., Khaitsa M.L., Antimicrobial drug resistance and molecular characterization of salmonella isolated from domestic animals, humans, and meat products, Foodborne Pathog. Dis. (2009) 6:273-284.
[163]	Olsen A.R., Hammack T.S., Isolation of Salmonella spp. from the housefly, Musca domestica L., and the dump fly, Hydrotaea aenescens (Wiedemann) (Diptera: Muscidae), at caged-layer houses, J. Food. Prot. (2000) 63:958-960.
[164]	Padron M., Salmonella typhimurium outbreak in broiler chicken flocks in Mexico, Avian Dis. (1990) 34:221-223.
[165]	Padungtod P., Kaneene J.B., Salmonella in food animals and humans in northern Thailand, Int. J. Food Microbiol. (2006) 108:346-354.
[166]	Palmer J.E., Benson C.E., Whitlock R.H., Salmonella shed by horses with colic, J. Am. Vet .Med. Assoc. (1985) 187:256-257.
[167]	Palmgren H., McCafferty D., Aspan A., Broman T., Sellin M., Wollin R., Bergstrom S., Olsen B., Salmonella in sub-Antarctica: low heterogeneity in Salmonella serotypes in South Georgian seals and birds, Epidemiol. Infect. (2000) 125:257-262.
[168]	Palmgren H., Aspan A., Broman T., Bengtsson K., Blomquist L., Bergstrom S., Sellin M., Wollin R., Olsen B., Salmonella in Black-headed gulls ( Larus ridibundus); prevalence, genotypes and influence on Salmonella epidemiology, Epidemiol. Infect. (2006) 134:635-644.
[169]	Pedersen K., Hansen H.C., Jorgensen J.C., Borck B., Serovars of Salmonella isolated from Danish turkeys between 1995 and 2000 and their antimicrobial resistance, Vet .Rec. (2002) 150:471-474.
[170]	Pedersen K., Lassen-Nielsen A.M., Nordentoft S., Hammer A.S., Serovars of Salmonella from captive reptiles, Zoonoses Public Health. (2009) 56:238-242.
[171]	Pennycott T.W., Park A., Mather H.A., Isolation of different serovars of Salmonella enterica from wild birds in Great Britain between 1995 and 2003, Vet. Rec. (2006) 158:817-820.
[172]	Pieskus J., Milius J., Michalskiene I., Zagrebneviene G., The distribution of Salmonella serovars in chicken and humans in Lithuania, J. Vet.Med. A Physiol. Pathol. Clin. Med. (2006) 53:12-16.
[173]	Poppe C., Irwin R.J., Forsberg C.M., Clarke R.C., Oggel J., The prevalence of Salmonella enteritidis and other Salmonella spp. among Canadian registered commercial layer flocks, Epidemiol. Infect. (1991) 106:259-270.
[174]	Poppe C., Irwin R.J., Messier S., Finley G.G., Oggel J., The prevalence of Salmonella enteritidis and other Salmonella sp. among Canadian registered commercial chicken broiler flocks, Epidemiol. Infect. (1991) 107:201-211.
[175]	Poppe C., Johnson R.P., Forsberg C.M., Irwin R.J., Salmonella enteritidis and other Salmonella in laying hens and eggs from flocks with Salmonella in their environment, Can. J. Vet. Res. (1992) 56:226-232.
[176]	Prost E., Riemann H., Food-borne salmonellosis, Annu Rev Microbiol. (1967) 21:495-528.
[177]	Radke B.R., McFall M., Radostits S.M., Salmonella Muenster infection in a dairy herd, Can. Vet. J. (2002) 43:443-453.
[178]	Reche M.P., Jimenez P.A., Alvarez F., Garcia de los Rios J.E., Rojas A.M., de Pedro P., Incidence of salmonellae in captive and wild free-living raptorial birds in central Spain, J. Vet. Med. B Infect. Dis. Vet. Public Health. (2003) 50:42-44.
[179]	Refsum T., Vikoren T., Handeland K., Kapperud G., Holstad G., Epidemiologic and pathologic aspects of Salmonella typhimurium infection in passerine birds in Norway, J. Wildl .Dis. (2003) 39:64-72.
[180]	Renter D.G., Gnad D.P., Sargeant J.M., Hygnstrom S.E., Prevalence and serovars of Salmonella in the feces of free-ranging white-tailed deer (Odocoileus virginianus) in Nebraska, J. Wildl. Dis. (2006) 42:699-703.
[181]	Rigby C.E., Pettit J.R., Changes in the Salmonella status of broiler chickens subjected to simulated shipping conditions, Can. J. Comp. Med. (1980) 44:374-381.
[182]	Rigby C.E., Pettit J.R., Baker M.F., Bentley A.H., Salomons M.O., Lior H., Sources of salmonellae in an uninfected commercially-processed broiler flock, Can. J. Comp. Med. (1980) 44:267-274.
[183]	Riley A H.M., Ramsey C, Tropical fish as a source of Salmonella java infection., Comm .Dis. Env. Health Scotland. (1992) 26:4-5.
[184]	Roberts M.C., O'Boyle D.A., The prevalence and epizootiology of salmonellosis among groups of horses in south east Queensland, Aust. Vet. J. (1981) 57:27-35.
[185]	Rodriguez-Buenfil J.C., Alvarez-Fleites M., Segura-Correa J.C., Incidence of salmonellosis and identification of serogroups and serotypes in a pig commercial farm in Yucatan, Rev. Latinoam. Microbiol. (2006) 48:10-13.
[186]	Roy R., Higgins R., Fortin M., Tardif S., Salmonella Give infection in 2 dairy herds, Can. Vet. J. (2001) 42:468-470.
[187]	Russel R.R., Tannock, G.W., The isolation of Salmonella Oranienburg from an ovine foetus in New Zealand, N.Z. Vet. J. (1964) 12:25-25.
[188]	Sandberg M., Alvseike O., Skjerve E., The prevalence and dynamics of Salmonella enterica IIIb 61:k:1,5,(7) in sheep flocks in Norway, Prev. Vet. Med. (2002) 52:267-275.
[189]	Sato Y., Schneebeli M., Matsukawa K., Chimana H., Sinsungwe H., Sato G., Outbreaks of Salmonella Dublin infection among calves on a dairy farm applying Salmonella bacterins in Zambia, J. Vet. Med .Sci. (1993) 55:511-513.
[190]	Schroter M., Speicher A., Hofmann J., Roggentin P., Analysis of the transmission of Salmonella spp. through generations of pet snakes, Environ. Microbiol. (2006) 8:556-559.
[191]	Schutze G.E., Sikes J.D., Stefanova R., Cave M.D., The home environment and salmonellosis in children, Pediatrics. (1999) 103:E1.
[192]	Seepersadsingh N., Adesiyun A.A., Prevalence and antimicrobial resistance of Salmonella spp. in pet mammals, reptiles, fish aquarium water, and birds in Trinidad, J. Vet. Med. B Infect. Dis. Vet. Public Health. (2003) 50:488-493.
[193]	Senanayake S.N., Ferson M.J., Botham S.J., Belinfante R.T., A child with Salmonella enterica serotype Paratyphi B infection acquired from a fish tank, Med .J. Aust. (2004) 180:250.
[194]	Sethi M.S., Sharma V.D., Singh S.P., The occurrence of salmonellae in zoo animals in Uttar Pradesh and Delhi (India), Int .J. Zoonoses. (1980) 7:15-18.
[195]	Sharp J.C., Reilly W.J., Linklater K.A., Inglis D.M., Johnston W.S., Miller J.K., Salmonella montevideo infection in sheep and cattle in Scotland, 1970-81, J. Hyg. (Lond). (1983) 90:225-232.
[196]	Silva E.N., Hipolito O., Grecchi R., Natural and experimental Salmonella arizonae 18:z4,z32 (Ar. 7:1,7,8) infection in broilers. Bacteriological and histopathological survey of eye and brain lesions, Avian Dis. (1980) 24:631-636.
[197]	Singer J.T., Opitz H.M., Gershman M., Hall M.M., Muniz I.G., Rao S.V., Molecular characterization of Salmonella enteritidis isolates from Maine poultry and poultry farm environments, Avian Dis. (1992) 36:324-333.
[198]	Singh B.R., Khurana S.K., Kulshreshtha S.B., Survivability of Salmonella paratyphi B var Java on experimentally infected cockroaches, Indian J. Exp. Biol. (1995) 33:392-393.
[199]	Skov M.N., Spencer A.G., Hald B., Petersen L., Nauerby B., Carstensen B., Madsen M., The role of litter beetles as potential reservoir for Salmonella enterica and thermophilic Campylobacter spp. between broiler flocks, Avian Dis. (2004) 48:9-18.
[200]	Smith B.P., Reina-Guerra M., Hardy A.J., Prevalence and epizootiology of equine salmonellosis, J. Am. Vet. Med. Assoc. (1978) 172:353-356.
[201]	Smith J.M., Robinson R.A., Salmonella typhimurium in New Zealand hedgehogs, N. Z. Vet. J. (1964) 12:111-112.
[202]	Smith W.A., Mazet J.A., Hirsh D.C., Salmonella in California wildlife species: prevalence in rehabilitation centers and characterization of isolates, J. Zoo Wildl. Med. (2002) 33:228-235.
[203]	Sparrow S., Diseases of pet rodents, J. Small Anim. Pract. (1980) 21:1-16.
[204]	Steffen E.K., Wagner J.E., Salmonella enteriditis serotype Amsterdam in a commercial rat colony, Lab. Anim. Sci. (1983) 33:454-456.
[205]	Sternberg S., Johnsson A., Aspan A., Bergstrom K., Kallay T.B., Szanto E., Outbreak of Salmonella Thompson infection in a Swedish dairy herd, Vet. Rec. (2008) 163:596-599.
[206]	Suwanrangsi S., Srimatyobhas, K.,  Keerativiryaporn, S., Incidence of Salmonella in Fishery Products:254-262.
[207]	Thorsteinsdottir T.R., Kristinsson K.G., Gunnarsson E., Antimicrobial resistance and serotype distribution among Salmonella spp. in pigs and poultry in Iceland, 2001-2005, Microb. Drug Resist. (2007) 13:295-300.
[208]	Tsai H.J., Hsiang P.H., The prevalence and antimicrobial susceptibilities of Salmonella and Campylobacter in ducks in Taiwan, J. Vet. Med.Sci. (2005) 67:7-12.
[209]	Tuchili L., Ulaya W., Kato Y., Kaneuchi C., Recent characterization of Salmonella strains isolated from chickens in Zambia, J. Vet. Med. Sci. (1996) 58:77-78.
[210]	van Duijkeren E., Sloet van Oldruitenborgh-Oosterbaan M.M., Houwers D.J., van Leeuwen W.J., Kalsbeek H.C., Equine salmonellosis in a Dutch veterinary teaching hospital, Vet. Rec. (1994) 135:248-250.
[211]	van Duijkeren E., Wannet W.J., Heck M.E., van Pelt W., Sloet van Oldruitenborgh-Oosterbaan M.M., Smit J.A., Houwers D.J., Sero types, phage types and antibiotic susceptibilities of Salmonella strains isolated from horses in The Netherlands from 1993 to 2000, Vet. Microbiol. (2002) 86:203-212.
[212]	van Duijkeren E., Wannet W.J., Houwers D.J., van Pelt W., Serotype and phage type distribution of salmonella strains isolated from humans, cattle, pigs, and chickens in the Netherlands from 1984 to 2001, J. Clin. Microbiol. (2002) 40:3980-3985.
[213]	Van Immerseel F., Pasmans F., De Buck J., Rychlik I., Hradecka H., Collard J.M., Wildemauwe C., Heyndrickx M., Ducatelle R., Haesebrouck F., Cats as a risk for transmission of antimicrobial drug-resistant Salmonella, Emerg. Infect.Dis. (2004) 10:2169-2174.
[214]	Van Kessel J.S., Karns J.S., Wolfgang D.R., Hovingh E., Schukken Y.H., Longitudinal study of a clonal, subclinical outbreak of Salmonella enterica subsp. enterica serovar Cerro in a U.S. dairy herd, Foodborne Pathog. Dis. (2007) 4:449-461.
[215]	Vanselow B.A., Hornitzky M.A., Walker K.H., Eamens G.J., Bailey G.D., Gill P.A., Coates K., Corney B., Cronin J.P., Renilson S., Salmonella and on-farm risk factors in healthy slaughter-age cattle and sheep in eastern Australia, Aust. Vet. J. (2007) 85:498-502.
[216]	Venter E.H., van Vuuren M., Carstens J., van der Walt M.L., Nieuwoudt B., Steyn H., Kriek N.P., A molecular epidemiologic investigation of Salmonella from a meat source to the feces of captive cheetah (Acinonyx jubatus), J. Zoo Wildl. Med. (2003) 34:76-81.
[217]	Vo A.T., van Duijkeren E., Fluit A.C., Heck M.E., Verbruggen A., Maas H.M., Gaastra W., Distribution of Salmonella enterica serovars from humans, livestock and meat in Vietnam and the dominance of Salmonella Typhimurium phage type 90, Vet. Microbiol. (2006) 113:153-158.
[218]	Wales A., Breslin M., Carter B., Sayers R., Davies R., A longitudinal study of environmental Salmonella contamination in caged and free-range layer flocks, Avian .Pathol. (2007) 36:187-197.
[219]	Weese J.S., Baird J.D., Poppe C., Archambault M., Emergence of Salmonella typhimurium definitive type 104 (DT104) as an important cause of salmonellosis in horses in Ontario, Can. Vet. J. (2001) 42:788-792.
[220]	White D.G., Datta A., McDermott P., Friedman S., Qaiyumi S., Ayers S., English L., McDermott S., Wagner D.D., Zhao S., Antimicrobial susceptibility and genetic relatedness of Salmonella serovars isolated from animal-derived dog treats in the USA, J. Antimicrob. Chemother. (2003) 52:860-863.
[221]	Wilkinson M.J., Taylor D.J., Laurie J., Brown C., Attempted eradication of salmonellosis from a colony of short-tail grey opossums (Monodelphis domestica), Lab. Anim. (2000) 34:217-222.
[222]	Wilson J.S., Hazel S.M., Williams N.J., Phiri A., French N.P., Hart C.A., Nontyphoidal salmonellae in United Kingdom badgers: prevalence and spatial distribution, Appl. Environ .Microbiol. (2003) 69:4312-4315.
[223]	Windsor R.S., Ashford W.A., Salmonella infection in the African elephant and the black rhinoceros, Trop. Anim. Health. Prod. (1972) 4:214-219.
[224]	Wong T.L., Thom K., Nicol C., Heffernan H., MacDiarmid S., Salmonella serotypes isolated from pet chews in New Zealand, J. Appl. Microbiol. (2007) 103:803-810.
[225]	Wray C., Sojka W.J., Bell J.C., Salmonella infection in horses in England and Wales, 1973 to 1979, Vet. Rec. (1981) 109:398-401.
[226]	Yokoyama E., Maruyama S., Kabeya H., Hara S., Sata S., Kuroki T., Yamamoto T., Prevalence and genetic properties of Salmonella enterica serovar typhimurium definitive phage type 104 isolated from Rattus norvegicus and Rattus rattus house rats in Yokohama City, Japan, Appl. Environ. Microbiol. (2007) 73:2624-2630.
