# Supplementary material for: Animal contact as a source of human non-typhoidal salmonellosis
Source: Vet Res. 2011 Feb 14;42(1):34. doi: 10.1186/1297-9716-42-34 (PMC3052180; doi:10.1186/1297-9716-42-34)
Supplement: Additional file 2 — Table S2. Documented reports of Salmonella transmission from birds to humans available in the peer-reviewed literature or otherwise published by public health agencies. Table S3. Documented reports of Salmonella transmission from reptiles, amphibians fish to humans available in the peer-reviewed literature or otherwise published by public health agencies. [file 1297-9716-42-34-S2.RTF]

Table S2: Documented reports of Salmonella transmissions from birds to humans available in the peer-reviewed literature or otherwise published by public health agencies. 
Outbreak source	Year	Salmonella serotype	Type of  contact	Additional information	Human cases	Country	Reference	
Pet birds								
chicken	2007	Montevideo	household, public setting (farm, feed store, fair)	chicken hatchlings	65	US	[19]	
chicken	1995/1996	Montevideo	household	multiple outbreaks	39	US	[12]	
chicken	1977	Arizonae^1^	household	Salmonella not isolated from chickens	1	US	[50]	
chicken	1962	Tennessee	household	chicken hatchlings	8	US	[43]	
chicken	1960	Enteritidis	household	chicken hatchlings	> 20	US	[43]	
chicken	1954	Typhimurium	household	chicken hatchlings	12	US	[2]	
duck	1991	Hadar	household	baby ducklings	22	US	[10]	
parakeet 	1975	Typhimurium	household	secondary cat infection	1	US	[39]	
poultry	2005-2007	Montevideo	household, public setting (farm, feed store, classroom, petting zoo)	baby poultry	64	US	[19]	
poultry	2006	4,5,12:i:-	household	baby poultry	21	US	[16]	
poultry	2006	Montevideo	household, public setting (daycare center)	baby poultry,	56	US	[16]	
poultry	2006	Ohio	household	baby poultry	4	US	[16]	
poultry	2002	Agona	public setting (daycare center) 	baby poultry in traveling animal show	7	Australia	[45]	
poultry	2000	Enteritidis	public setting (daycare center)	baby poultry	32	UK	 [13] 	
poultry	1999	Infantis	household	baby poultry	21	US	[13]	
poultry	1999	Typhimurium	household	baby poultry	40	US	[13]	
poultry	1972	Agona	occupational (farm)	animal feed potential source	n/a	US	[31]	
poultry	1971	Thompson	occupational (farm)	animal feed potential source	n/a	US	[31]	
Wild birds								
Owl pellets	2001	Typhimurium	public setting (school)	contaminated environment, 2 separate outbreaks	40	US	[55, 58]	
Wild birds	2000	Typhimurium	unclear	indirect contact, potentially contaminated water	n/a	New Zealand	[1]	
Wild birds	1999	Typhimurium	unclear	indirect contact with seagulls, potentially contaminated water	n/a	Norway	[37]	
Wild birds	1990-1992^a^	Typhimurium	unclear	contact to droppings or contaminated water	n/a	Norway	[35]	
Wild birds	1987	Typhimurium	unclear	Potentially contact to contaminated chocolate	349	Norway, Finland	[33, 34, 37]	

^a^ duration of observational study
^1^ Salmonella enterica subspecies arizonae 
n/a exact number of cases not specified 
Table S3: Documented reports of Salmonella transmissions from reptiles, amphibians or fish to humans available in the peer-reviewed literature or otherwise published by public health agencies. 

Outbreak Source	Year	Salmonella serotype	Type of  contact	Additional information	Human cases	Country	Reference	
Reptiles								
Lizard	2008	Ago	n/a 	antigenic formula: 30:z_38_:-	1	Germany	[6]	
Lizard	2008	Apapa	household	indirect contact	1	UK	[23]	
Lizard	2008	Ealing	n/a 	antigenic formula:  35:g,m,s:-)	1	Germany	[6]	
Lizard	2008	Gaminara	n/a 	antigenic formula: 16:d:1,7	1	Germany	[6]	
Lizard	2008	Subsp. II	n/a 	antigenic formula: 58:lz13,z_28_:z_6_	1	Germany	[6]	
Lizard	2008	Subsp.IV	n/a 	antigenic formula: 18:z_36_,z_38_:-	1	Germany	[6]	
Lizard	2007	Stanley	n/a	-	1	Ireland	[6]	
Lizard	2007	Subsp. II	n/a 	antigenic formula: 47:d:z_39_	1	Germany	[6]	
Lizard	2007	Subsp. II	n/a 	antigenic formula: 58:-:1,6	1	Germany	[6]	
Lizard	2007	Subsp. IV	n/a 	antigenic formula: 45:g,z_51_:-	1	Germany	[6]	
Lizard	2006	Apapa	household	-	1	Germany	[6]	
Lizard	2006	Marina	n/a 	antigenic formula: 48:g,z_51_:-	1	Germany	[6]	
Lizard	2006	Monschaui	household	previously also infected	1	Ireland	[6, 41]	
Lizard	2006	Pomona	household 	neighbor house	1	German	[6]	
Lizard	2006^b^	Telelkebir	household	-	1	Switzerland	[6]	
Lizard	2006	Subsp. II	n/a 	antigenic formula: 50:z:z_23_	1	Germany	[6]	
Lizard	2006	Subsp. IV	n/a 	antigenic formula: 45:g,z51:-)	1	Germany	[6]	
Lizard	2005	Kingabwa	household	some exposures unclear	6	US	[29]	
Lizard	2005	Minnesota	household	infected again in 2006  	1	Ireland	[6, 41]	
Lizard	2002	Poona	household	-	2	US	[14]	
Lizard	2002	Subsp. IV	household,	indirect contact ; antigenic formula: 44:z_4_:z_23_:-	2	US	[14]	
Lizard	2000	Arizonae	household	-	1	US	[47]	
Lizard	2000	Poona	household, 	indirect contact, 2 separate cases	2	UK	[52] [47]	
Lizard	2000 ^b^	Rubislaw	household	-	1	UK	[3]	
Lizard	2000	Tennessee	household	indirect contact	1	US	[14]	
Lizard	2000 ^b^	Subsp. IV	household 	antigenic formula: 44:22:z_23_	1	Italy	[3]	
Lizard	1999^b^	Chameleon	household	serotype not isolated from lizard	1	UK	[25]	
Lizard	1999^b^	Virchow	household	indirect contact	1	France	[48]	
Lizard	1998^b^	Arizonae	household	-	1	US	[49]	
Lizard	1998	Marina	household, 	indirect contact	4	US	[4] [14]	
Lizard	1997^b^	Poona	household,	-	1	US	[63]	
Lizard	1997	Saintpaul	household	-	1	US	[4]	
Lizard	1996	Enteritidis	public setting (zoo) 	indirect, environmental contamination	65	US	[5, 6, 27]	
Lizard	1996	Subsp. IV	household	indirect contact; antigenic formula: 44, _Z4_, _Z23_:-	1	US	[4]	
Lizard	1996 ^b^	Marina	household	-	1	US	[26]	
Lizard	1996 ^b^	Montevideo	household	-	2	US	[26]	
Lizard	1994-1996^c^	Abaetetuba	household	-	2	Canada	[62]	
Lizard	1994-1996^c^	Marina	household	multiple separate cases	4	Canada	[62]	
Lizard	1994-1996^c^	Montevideo	household	-	1	Canada	[62]	
Lizard	1994-1996^c^	Wassenaar	household	multiple separate cases	6	Canada	[62]	
Lizard	1995 ^b^	Durban	household	lizard not tested	1	UK	[42]	
Lizard	1995	Wassenaar	household	-	1	US	[11]	
Lizard	1994/1995	Marina	household	multiple cases	n/a	US	[44]	
Lizard	1994	Kintambo	household	secondary human-to-human	3	US	[11]	
Lizard	1994	Poona	household	indirect contact	1	US	[11]	
Lizard	1994	Rubislaw	household	babysitter home, indirect contact	3	US	[11]	
Lizard/Snake	2008	Subsp.IV	n/a 	antigenic formula: 44:z_4_,z_23_:-; 2 separate outbreaks	1	Germany	[6]	
Lizard/Snake	2005	Typhimurium	household	2 separate cases	2	France	[6]	
Lizard/Turtle	2000	Sandiego	household	-	7	US	[14]	
Lizard/Turtle	1994-1996^c^	Poona	househol	multiple separate cases	2	Canada	[62]	
Snake	2008	Arizonae^1^	household, 	indirect contact, 3 separate cases	3	Belgium	[6]	
Snake	2008	Arizonae	household	indirect contact	1	France	[53]	
Snake	2008	Diarizonae^2^	n/a 	antigenic formula: 53:z_10_:-	1	Germany	[6]	
Snake	2008	Diarizonae	n/a 	antigenic formula: 61:l,v:1,5,7	1	Germany	[6]	
Snake	2008	Diarizonae	n/a 	antigenic formula: 50:r:z_35_	1	Germany	[6]	
Snake	2008	Diarizonae	n/a 	antigenic formula: 61:z52:z_53_	1	Germany	[6]	
Snake	2008	Poona	n/a 	antigenic formula: 13,22:z:1,6	1	Germany	[6]	
Snake	2007	Arizonae	household or public setting (reptile farm)	-	1	Ireland	[6, 41]	
Snake	2007	Arizonae	n/a 	antigenic formula: 41:z_4_.z_23_:-)	1	Germany	[6]	
Snake	2007	Subsp.IV	n/a 	antigenic formula: 44,z_4_,z_23_:-	1	Germany	[6]	
Snake	2006	Arizonae	n/a 	antigenic formula: 41:z_4_,z_23_:-	1	German	[6]	
Snake	2006	Diarizonae	household	-	1	Ireland	[6, 41]	
Snake	2006	Diarizonae	n/a 	antigenic formula: 47:i:z_53_	1	German	[6]	
Snake	2006	Diarizonae	n/a 	antigenic formula: 50:z:z_23_	1	German	[6]	
Snake	2006	Subsp. IV	n/a 	antigenic formula: 44:z_4_:z_23_:-)	1	German	[6]	
Snake	2006	Subsp. IV	n/a 	antigenic formula: 50:g,z_51_:-	1	Germany	[6]	
Snake	2003	Arizonae	household 	father snake charmer	1	India	[40]	
Snake	2001	Enteritidis	household	secondary: platelet transfusion	3	US	[32]	
Snake	2001	Nima	household	transmission through clothes	1	US	[14]	
Snake	1998	Uzaramo	household	-	1	UK	[47]	
Snake	1997	Typhimurium	household	-	2	US	[4]	
Snake	1996 ^b^	Arizonae	household	snake not tested	1	UK	[8]	
Terrapin	2008	Pomona	household	-	1	Ireland	[6]	
Terrapin	2008	Thompson	household	-	1	Ireland	[6]	
Terrapin	2007	Pomona	household	indirect contact	1	Ireland	[6, 41]	
Terrapin	2006	Enteritidis	household	-	1	Ireland	[6, 41]	
Terrapin	1998^b^	Telelkebir	household	-	8	Ireland	[38]	
Tortoise 	2005	Michigan	household	wound infection	1	UK	[30]	
Turtle	2008	Abony	household	indirect contact	1	Belgium	[61]	
Turtle	2008	Infantis	household	-	1	Ireland	[6]	
Turtle	2008	Pomona	n/a 	antigenic formula: 28:y:1,7	1	Germany	[6]	
Turtle	2008	Typhimurium	household 	Secondary cases	135	US	[21]	
Turtle	2007	Paratyphi B	household	multiple separate cases, contaminated ground pool	103	US	[18]	
Turtle	2007	Pomona	household	-	20	US	[17]	
Turtle	2007	Pomona	household	-	1	Belgium	[7]	
Turtle	2006	4,5,12:i:-	household, 	2 separate cases	3	US	[17]	
Turtle	2005	Apapa	household	antigenic formula: 45:m,t,:-)	3	Germany	[6]	
Turtle	2005	Braenderup	household	-	6	Finland	[6]	
Turtle	2005	Paratyphi B	household	-	1	Japan	[47]	
Turtle	2004	Pomona	household	multiple separate cases	6	US	[15]	
Turtle	2004	Typhimurium	household	2 separate cases	2	US	[15]	
Turtle	2002^b^	Enteritidis	household	non-commercial breeder	1	Netherlands	[57]	
Turtle	1994-1996^c^	Braederup	household	-	1	Canada	[62]	
Turtle	1994-1996^c^	Jangwani	household	multiple separate cases	10	Canada	[62]	
Turtle	1994-1996^c^	Paratyphi B	household	-	1	Canada	[62]	
Turtle	1994	Stanley	household	indirect contact	1	US	[11]	
Turtle	1986	Typhimurium	household	-	2	US	[9]	
Turtle	1983/1984 ^c^	Pomona	household	-	n/a	Yugoslavia	[59]	
Turtle	1984	Pomona 	household	Salmonella serotype from human samples unknown	n/a	Puerto Rico	[59]	
Turtle	1962	Hartford	household	-	1	US	[22, 59]	
Reptiles	2008	Infantis	n/a^a^	-	1	Ireland	[6]	
Reptiles	2008	Jangwani	n/a 	antigenic formula: 17:a:1,5	1	Germany	[6]	
Reptiles	2006	Florida	household	-	1	Ireland	[6]	
Reptiles	2006	Subsp. II	n/a 	antigenic formula: 58:c:z_6_)	1	German	[6]	
Reptiles	2006	Subsp. IV	n/a 	antigenic formula: 44:-:-)	1	German	[6]	
Reptiles	1994	Arizonae	household or occupational (pet shop) 	-	1	US	[11]	
								
Amphibians								
frogs	2009	Typhimurium	household	pet African dwarf frog source	85	US	[20]	
frogs / toads 	2001	Javiana	unclear	wild frogs or toads suspected	>55	US	[56]	
frogs	2000	Saintpaul	public setting (construction site)	contaminated water, source unclear		Australia	[60]	
								
Fish								
fish tank	2007	Paratyphi B	household	potentially bird contact contributed	1	Ireland	[6]	
fish tank	2003/2004	Paratyphi B	household	-	28	Australia	[46, 54]	
fish tank	2000-2002	Paratyphi B	household	-	7	Canada	[28]	
fish tank	2000	Montevideo	household or public setting (pet shop)	Salmonella not isolated from fish	1	UK	[36]	
fish tank	1992	Paratyphi B	household	multiple separate cases	2	UK	[51]	
fish tank	1981	Paratyphi B	household	multiple separate cases	n/a	UK	[24]	

^a^ no information available; ^b^ exact time of outbreak not available; ^c^ only time range specified
^1^ Salmonella enterica subsp. arizonae (Salmonella subsp. IIIa); ^2^ Salmonella enterica subsp. diarizonae (Salmonella subsp. IIIb)

REFERENCES
[1] 	Alley M.R., Connolly J.H., Fenwick S.G., Mackereth G.F., Leyland M.J., Rogers L.E., Haycock M., Nicol C., Reed C.E., An epidemic of salmonellosis caused by Salmonella Typhimurium DT160 in wild birds and humans in New Zealand, N. Z. Vet. J. (2002) 50:170-176.	
 [2]	Anderson A., Bauer, H and Nelson, CB., Salmonellosis due to Salmonella Typhimurium with easter chicks as likely source., J. Am. Med. Assoc. (1955) 158:1153-1155.
[3]	Andreacchio A., Miller F., Salmonella osteomyelitis transmitted from an iguana, Orthopedics. (2000) 23:1201-1202.
[4]	Anonymous, Reptile-associated salmonellosis--selected states, 1996-1998, Can. Commun. Dis. Rep. (2000) 26:19-23..
[5]	Bender J.B., Shulman S.A., Reports of zoonotic disease outbreaks associated with animal exhibits and availability of recommendations for preventing zoonotic disease transmission from animals to people in such settings, J. Am. Vet. Med. Assoc. (2004) 224:1105-1109.
[6]	Bertrand S., Rimhanen-Finne R., Weill F.X., Rabsch W., Thornton L., Perevoscikovs J., van Pelt W., Heck M., Salmonella infections associated with reptiles: the current situation in Europe, Euro Surveill. (2008) 13:pii=18902
[7]	Bredart S., Wastelin M., Collard J.M., Coppee M., Bodart E., [Pet turtle and septicemia: what is the relationship?], Rev. Med. Liege. (2007) 62:496-497.
 [8]	Buck J.J., Nicholls S.W., Salmonella arizona enterocolitis acquired by an infant from a pet snake, J. Pediatr. Gastroenterol. Nutr. (1997) 25:248-249.
[9]	CDC (Centers for Disease Control and Prevention), Turtle-associated salmonellosis--Ohio, MMWR Morb. Mortal. Wkly Rep. (1986) 35:733-734, 739.
 [10]	CDC (Centers for Disease Control and Prevention), Salmonella Hadar associated with pet ducklings--Connecticut, Maryland, and Pennsylvania, 1991, MMWR Morb. Mortal. Wkly. Rep. (1992) 41:185-187
[11]	CDC (Centers for Disease Control and Prevention), Reptile-associated salmonellosis--selected states, 1994-1995, MMWR Morb. Mortal. Wkly. Rep. (1995) 44:347-350.
[12]	CDC (Centers for Disease Control and Prevention), Salmonella serotype Montevideo infections associated with chicks--Idaho, Washington, and Oregon, spring 1995 and 1996, MMWR Morb. Mortal. Wkly. Rep. (1997) 46:237-239.
 [13]	CDC (Centers for Disease Control and Prevention), Salmonellosis associated with chicks and ducklings--Michigan and Missouri, Spring 1999, MMWR Morb. Mortal. Wkly. Rep. (2000) 49:297-299.
 [14]	CDC (Centers for Disease Control and Prevention), Reptile-associated salmonellosis--selected states, 1998-2002, MMWR Morb. Mortal. Wkly. Rep. (2003) 52:1206-1209. [15]	CDC, Salmonellosis associated with pet turtles--Wisconsin and Wyoming, 2004, MMWR Morb Mortal Wkly Rep. (2005) 54:223-226.
[16]	CDC (Centers for Disease Control and Prevention), Three outbreaks of salmonellosis associated with baby poultry from three hatcheries--United States, 2006, MMWR Morb. Mortal. Wkly. Rep. (2007) 56:273-276. 
[17]	CDC (Centers for Disease Control and Prevention), Turtle-associated salmonellosis in humans--United States, 2006-2007, MMWR Morb. Mortal. Wkly. Rep. (2007) 56:649-652.
 [18]	Multistate outbreak of human Salmonella infections associated with exposure to turtles--United States, 2007-2008, MMWR Morb. Mortal. Wkly. Rep. (2008) 57:69-72.
[19]	Multistate outbreaks of Salmonella infections associated with live poultry--United States, 2007, MMWR Morb. Mortal. Wkly. Rep. (2009) 58:25-29.

[20]	Multistate outbreak of human Salmonella Typhimurium infections associated with aquatic frogs - United States, 2009, MMWR Morb. Mortal. Wkly. Rep. (2009) 58:1433-1436.
 [21]	CDC (Centers for Disease Control and Prevention), Multistate outbreak of human Salmonella Typhimurium infections associated with pet turtle exposure - United States, 2008, MMWR Morb. Mortal. Wkly. Rep. (2010) 59:191-196. 
[22]	CDC (Centers for Disease Control and Prevention)., Salmonella Surveillance Report No. 10. (1963):22.
 [23]	Cooke F.J., De Pinna E., Maguire C., Guha S., Pickard D.J., Farrington M., Threlfall E.J., First report of human infection with Salmonella enterica serovar Apapa resulting from exposure to a pet lizard, J. Clin. Microbiol. (2009) 47:2672-2674.
[24]	Cox R.A.,  Robinson J., Salmonella Java from tropical fish tanks, Communicable Disease Report. (1981) 31:4.
 [25]	Cyriac J., Wozniak E.R., Infantile Salmonella meningitis associated with gecko-keeping, Commun. Dis. Public Health. (2000) 3:66-67.
[26]	Embil J.M., Nicolle L.E., Salmonella urinary tract infections associated with exposure to pet iguanas, Clin. Infect. Dis. (1997) 25:172.
 [27]	Friedman C.R., Torigian C., Shillam P.J., Hoffman R.E., Heltzel D., Beebe J.L., Malcolm G., DeWitt W.E., Hutwagner L., Griffin P.M., An outbreak of salmonellosis among children attending a reptile exhibit at a zoo, J. Pediatr. (1998) 132:802-807.
[28]	Gaulin C., Vincent C., Alain L., Ismail J., Outbreak of Salmonella Paratyphi B linked to aquariums in the province of Quebec, 2000, Can. Commun. Dis. Rep. (2002) 28:89-93, 96.
 [29]	Greene S., Yartel A., Moriarty K., Nathan L., Salehi E., Tengelsen L., Patel N., Lynch M., Salmonella Kingabwa infections and lizard contact, United States, 2005, Emerg. Infect. Dis. (2007) 13:661-662.
 [30]	Hames A., Mumford J., Hale J., Galloway A., Salmonella Michigan soft tissue infection in an immunocompromised child, J. Clin. Pathol. (2008) 61:773-774.
[31]	Holmberg S.D., Wells J.G., Cohen M.L., Animal-to-man transmission of antimicrobial-resistant Salmonella: investigations of U.S. outbreaks, 1971-1983, Science. (1984) 225:833-835.
 [32]	Jafari M., Forsberg J., Gilcher R.O., Smith J.W., Crutcher J.M., McDermott M., Brown B.R., George J.N., Salmonella sepsis caused by a platelet transfusion from a donor with a pet snake, N. Engl. J. Med. (2002) 347:1075-1078.
[33]	Kapperud G., Lassen J., Dommarsnes K., Kristiansen B.E., Caugant D.A., Ask E., Jahkola M., Comparison of epidemiological marker methods for identification of Salmonella Typhimurium isolates from an outbreak caused by contaminated chocolate, J. Clin. Microbiol. (1989) 27:2019-2024.
[34]	Kapperud G., Gustavsen S., Hellesnes I., Hansen A.H., Lassen J., Hirn J., Jahkola M., Montenegro M.A., Helmuth R., Outbreak of Salmonella Typhimurium infection traced to contaminated chocolate and caused by a strain lacking the 60-megadalton virulence plasmid, J. Clin. Microbiol. (1990) 28:2597-2601.
 [35]	Kapperud G., Stenwig H., Lassen J., Epidemiology of Salmonella Typhimurium O:4-12 infection in Norway: evidence of transmission from an avian wildlife reservoir, Am. J. Epidemiol. (1998) 147:774-782.
 [36]	Katsoulis E., Pallett A., Bowyer G.W., Septic arthritis of the knee by Salmonella Montevideo, Ann. R. Coll. Surg. Engl. (2004) 86:272-274.
[37]	Kruse H.,Kirkemo A.-M., Handeland K., Wildlife as source of zoonotic infections., Emerg. Infect. Dis. (2004) 10.
[38]	Lynch M., Daly M., O'Brien B., Morrison F., Cryan B., Fanning S., Salmonella Tel-el-kebir and terrapins, J. Infect. (1999) 38:182-184.
 [39]	Madewell B.R., McChesney A.E., Salmonellosis in a human infant, a cat, and two parakeets in the same household, J. Am. Vet. Med. Assoc. (1975) 167:1089-1090.
 [40]	Mahajan R.K., Khan S.A., Chandel D.S., Kumar N., Hans C., Chaudhry R., Fatal case of Salmonella enterica subsp. arizonae gastroenteritis in an infant with microcephaly, J. Clin. Microbiol. (2003) 41:5830-5832.
 [41]	Mahon M., O'Byrne, A.M., Reptile-associated salmonellosis in residents in the south east of Ireland, 2005 - 07, Epi-Insight. (2008) April.
 [42]	Makin G., Abu-Harb M., Finn A., Partridge S., Salmonella Durban in an infant, Lancet. (1996) 348:200.
 [43]	McCroan J.E., McKinley T.W., Brim A., Ramsey C.H., Five Salmonellosis Outbreaks Related to Poultry Products, Public Health Rep. (1963) 78:1073-1080.
[44]	Mermin J., Hoar B., Angulo F.J., Iguanas and Salmonella Marina infection in children: a reflection of the increasing incidence of reptile-associated salmonellosis in the United States, Pediatrics. (1997) 99:399-402.
 [45]	Merritt T.D., Herlihy C., Salmonella outbreak associated with chicks and ducklings at childcare centres., Med. J. Aust. . (2003) 179:63-64.
 [46]	Musto J., Kirk M., Lightfoot D., Combs B.G., Mwanri L., Multi-drug resistant Salmonella Java infections acquired from tropical fish aquariums, Australia, 2003-04, Commun. Dis. Intell. (2006) 30:222-227.
 [47]	Nagano N., Oana S., Nagano Y., Arakawa Y., A severe Salmonella enterica serotype Paratyphi B infection in a child related to a pet turtle, Trachemys scripta elegans, Jpn. J. Infect. Dis. (2006) 59:132-134.
 [48]	Neuwirth C., Francois C., Laurent N., Pechinot A., Myocarditis due to Salmonella Virchow and sudden infant death, Lancet. (1999) 354:1004.
[49]	Nowinski R.J., Albert M.C., Salmonella osteomyelitis secondary to iguana exposure, Clin. Orthop. Relat. Res. (2000):250-253.
 [50]	Petru M.A., Richman D.D., Arizona hinshawii infection of an atherosclerotic abdominal aorta, Arch. Intern. Med. (1981) 141:537-538.
[51]	Riley A., Hanson M., Ramsey C., Tropical fish as a source of Salmonella Java infection., Comm. Dis. Env. Health Scotland. (1992) 26:4-5.
 [52]	Sam W.I., Mackay A.D., Salmonella meningitis and a green iguana, J. R. Soc. Med. (2000) 93:318-319. 
[53]	Schneider L., Ehlinger M., Stanchina C., Giacomelli M.C., Gicquel P., Karger C., Clavert J.M., Salmonella enterica subsp. arizonae bone and joints sepsis. A case report and literature review, Orthop. Traumatol. Surg. Res. (2009) 95:237-242.
[54]	Senanayake S.N., Ferson M.J., Botham S.J., Belinfante R.T., A child with Salmonella enterica serotype Paratyphi B infection acquired from a fish tank, Med. J. Aust. (2004) 180:250.
[55]	Smith K.E., Anderson F., Medus C., Leano F., Adams J., Outbreaks of salmonellosis at elementary schools associated with dissection of owl pellets, Vector Borne Zoonotic Dis. (2005) 5:133-136.
 [56]	Srikantiah P L.J., Crump JA, et al., An outbreak of Salmonella Javiana associated with amphibian contact---Mississippi, 2001. , Presented at the International Conference on Emerging Infectious Diseases, Atlanta, Georgia. (2002).
[57]	Stam F., Romkens T.E., Hekker T.A., Smulders Y.M., Turtle-associated human salmonellosis, Clin. Infect. Dis. (2003) 37:e167-169. 
[58]	Steinmuller N., Demma L., Bender J.B., Eidson M., Angulo F.J., Outbreaks of enteric disease associated with animal contact: not just a foodborne problem anymore, Clin. Infect. Dis. (2006) 43:1596-1602.
[59]	Tauxe R.V., Rigau-Perez J.G., Wells J.G., Blake P.A., Turtle-associated salmonellosis in Puerto Rico. Hazards of the global turtle trade, J. Am. Vet. Med. Assoc. (1985) 254:237-239.

[60]	Taylor R., Sloan D., Cooper T., Morton B., Hunter I., A waterborne outbreak of Salmonella Saintpaul, Commun. Dis. Intell. (2000) 24:336-340.
[61]	Van Meervenne E., Botteldoorn N., Lokietek S., Vatlet M., Cupa A., Naranjo M., Dierick K., Bertrand S., Turtle-associated Salmonella septicaemia and meningitis in a 2-month-old baby, J. Med. Microbiol. (2009) 58:1379-1381.
[62]	Woodward D.L., Khakhria R., Johnson W.M., Human salmonellosis associated with exotic pets, J. Clin. Microbiol. (1997) 35:2786-2790. 
[63]	Wu C.C., de Gortari M.J., Lin T.L., Barrett B., Ribotyping of Salmonella Poona in iguana-associated zoonotic salmonellosis, J. Vet. Diagn. Invest. (1998) 10:188-190.
